# Trapoxin A Analogue as a Selective Nanomolar Inhibitor of HDAC11

**DOI:** 10.1021/acschembio.2c00840

**Published:** 2023-03-28

**Authors:** Thanh Tu Ho, Changmin Peng, Edward Seto, Hening Lin

**Affiliations:** † Department of Chemistry and Chemical Biology, Cornell University, Ithaca, New York 14853, United States; ‡ Department of Biochemistry & Molecular Medicine, School of Medicine & Health Sciences, George Washington Cancer Center, George Washington University, Washington, District of Columbia 20037, United States; § Howard Hughes Medical Institute, Cornell University, Ithaca, New York 14853, United States

## Abstract

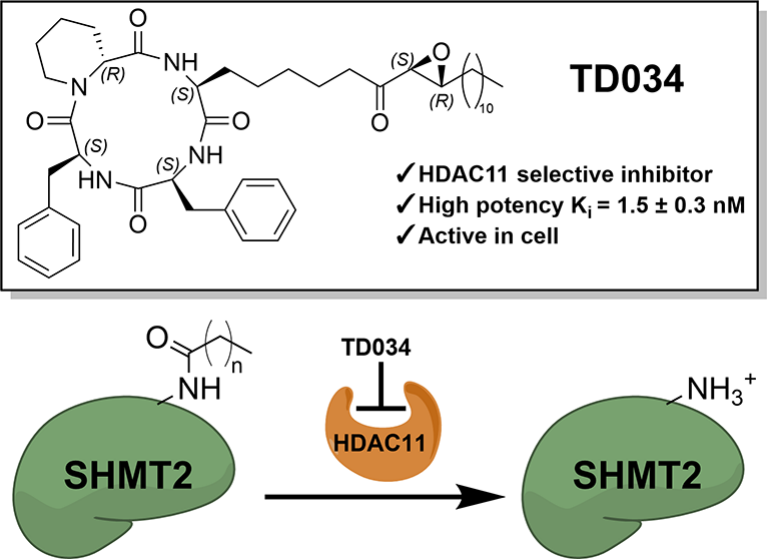

Histone deacetylases (HDACs) are enzymes that regulate many important biological pathways. There is a need for the development of isoform-selective HDAC inhibitors for further biological applications. Here, we report the development of trapoxin A analogues as potent and selective inhibitors of HDAC11, an enzyme that can efficiently remove long-chain fatty acyl groups from proteins. In particular, we show that one of the trapoxin A analogues, TD034, has nanomolar potency in enzymatic assays. We show that in cells, TD034 is active at low micromolar concentrations and inhibits the defatty acylation of SHMT2, a known HDAC11 substrate. The high potency and selectivity of TD034 would permit further development of HDAC11 inhibitors for biological and therapeutic applications.

## Introduction

Histone deacetylases (HDACs) were originally described as a class of enzymes that can remove the acetyl group from protein lysine residues.^1^ In humans, there are 11 HDACs that use a Zn^2+^-dependent mechanism for substrate deacetylation. HDACs can regulate chromatin structure and transcription through the deacetylation of histones but are also involved in other cellular processes through the regulation of nonhistone substrates.^2,3^ Histone deacetylase 11 (HDAC11) is the smallest and the last discovered HDAC, and a sole member of class IV HDAC.^4^ Its biological function is not yet fully elucidated. We and others showed that HDAC11 has a high defatty-acylase activity, while its deacetylase activity is essentially undetectable.^5−7^ We also found that serine hydroxymethyltransferase 2 (SHMT2) is a physiological substrate of HDAC11.^5^ The defatty acylation of SHMT2 by HDAC11 leads to increased type I interferon signaling in both cells and mouse models,^5^ which suggests that the inhibition of HDAC11 has the potential to treat diseases by modulating immune response. There have been other reports suggesting that the inhibition of HDAC11 could be beneficial for treating cancers,^8,9^ obesity,^10^ and multiple sclerosis.^11^ Therefore, there is a need for highly potent and specific HDAC11 inhibitorsto further study its biological function and explore the therapeutic potential of inhibiting HDAC11.

**Figure 1:**
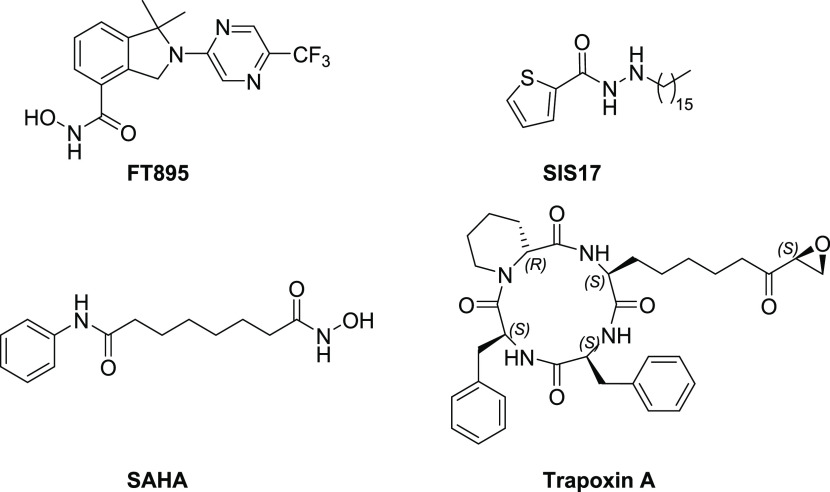
Structure of known inhibitors.

The earliest known selective HDAC11 inhibitor is FT895 (Figure 1), which was developed by Forma Therapeutics.^12^ Based on its efficient catalytic activity in removing long-chain fatty acyl groups, we surmised that HDAC11 contains a hydrophobic pocket close to its Zn^2+^ catalytic center. Thus, our laboratory developed another HDAC11 inhibitor, SIS17 (Figure 1), which can fit this hydrophobic pocket.^13^ Both FT895 and SIS17 display low micromolar inhibition of HDAC11 demyristoylation activity *in vitro.*(13) Surprisingly, SAHA (Figure 1), an FDA-approved HDAC inhibitor, cannot efficiently inhibit HDAC11’s demyristoylation activity.^13^ Meanwhile, trapoxin A (Figure 1), a class I HDAC inhibitor,^14^ can inhibit HDAC11 in the sub-micromolar range, although its nonselective HDAC inhibition activities limit its usefulness for studying HDAC11. We hypothesized that the modification of trapoxin A to exploit hydrophobic acyl pocket can yield potent, specific inhibitors for HDAC11.

## Results and Discussion

### Design and Synthesis of Trapoxin A Analogues

**Scheme 1:**
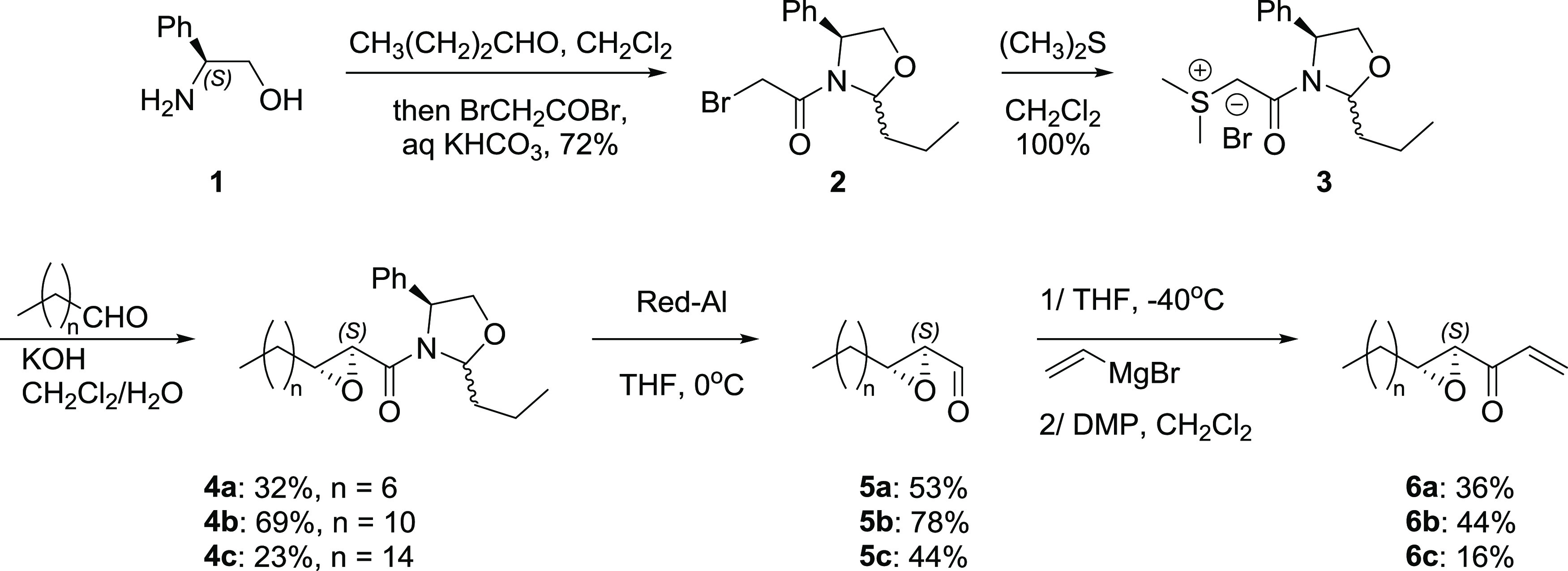
Synthesis of the Epoxyketone Motif

Schreiber^15^ and Kazmaier^16^ developed the only syntheses for trapoxin A and analogues. Despite its promising activity, very few synthetic derivatives of trapoxin A have been reported due to difficulties in modifying its structure. For our synthesis, we started by preparing various epoxyketone motifs containing long hydrocarbon chains at the β-position (Scheme 1) and α-position (Scheme S2, Supporting Information). Oxazolidine sulfur ylide **3**(17) was prepared from (S)-phenylglycinol, and subsequent reactions with aliphatic aldehydes of various lengths afforded glycidyl amides **4** with (S)-configuration. Careful reduction with Red-Al provided unstable epoxy aldehyde **5**, which was reacted with vinylmagnesium bromide at −40 °C, followed by oxidation with Dess-Martin periodinane to afford vinyl ketones **6**. The highest overall yield of **6** from **3** is obtained for **6b** (24%); other chain lengths lead to poor yield and lengthy purification (6% for **6a** and 1.6% for **6c**).

**Scheme 2:**
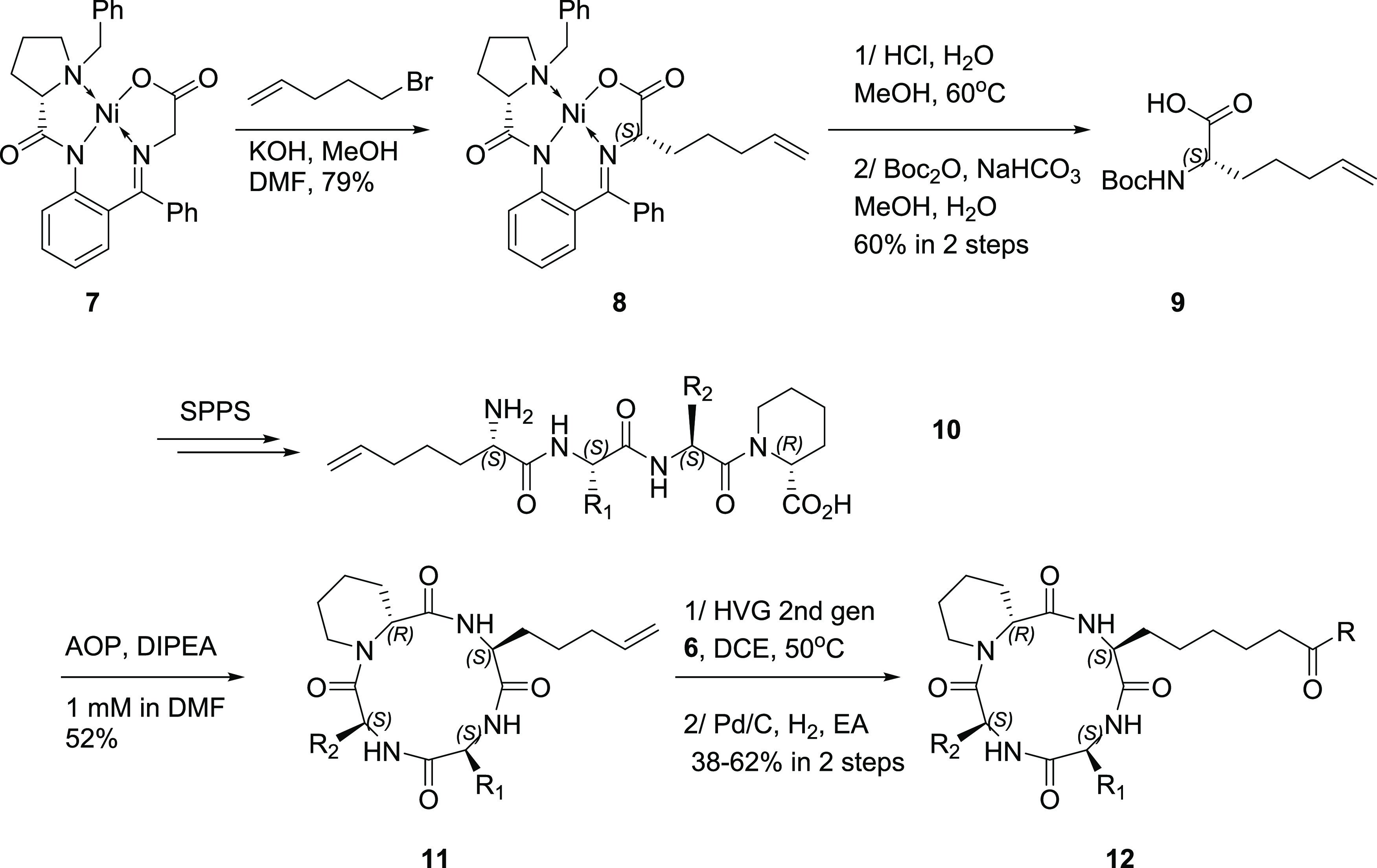
Synthesis of the Cyclic Peptides

For the cyclic peptide backbone, we synthesized the unnatural amino acid (Uaa, **9**), whose terminal alkene provided the anchor for subsequent olefin metathesis (Scheme 2). Alkylation of (S)-BPB-Ni-Gly complex **7** with 5-bromo-1-pentene under strictly air-free conditions afforded **8** stereoselectively.^18^ Acidic methanolysis of **8**, ion-exchange purification followed by Boc protection provided Boc-Uaa-OH **9**. Solid-phase peptide synthesis afforded linear tetrapeptide **10**, which was cyclized under high dilution conditions using AOP as the coupling reagent to yield cyclic peptide **11**. Olefin metathesis of **11** with **6** using Hoveyda Grubbs second-generation catalyst, followed by Pd/C-catalyzed hydrogenation afforded the final inhibitors **12**.

### *In Vitro* Testing of the Synthesized Trapoxin A Analogues

**Figure 2:**
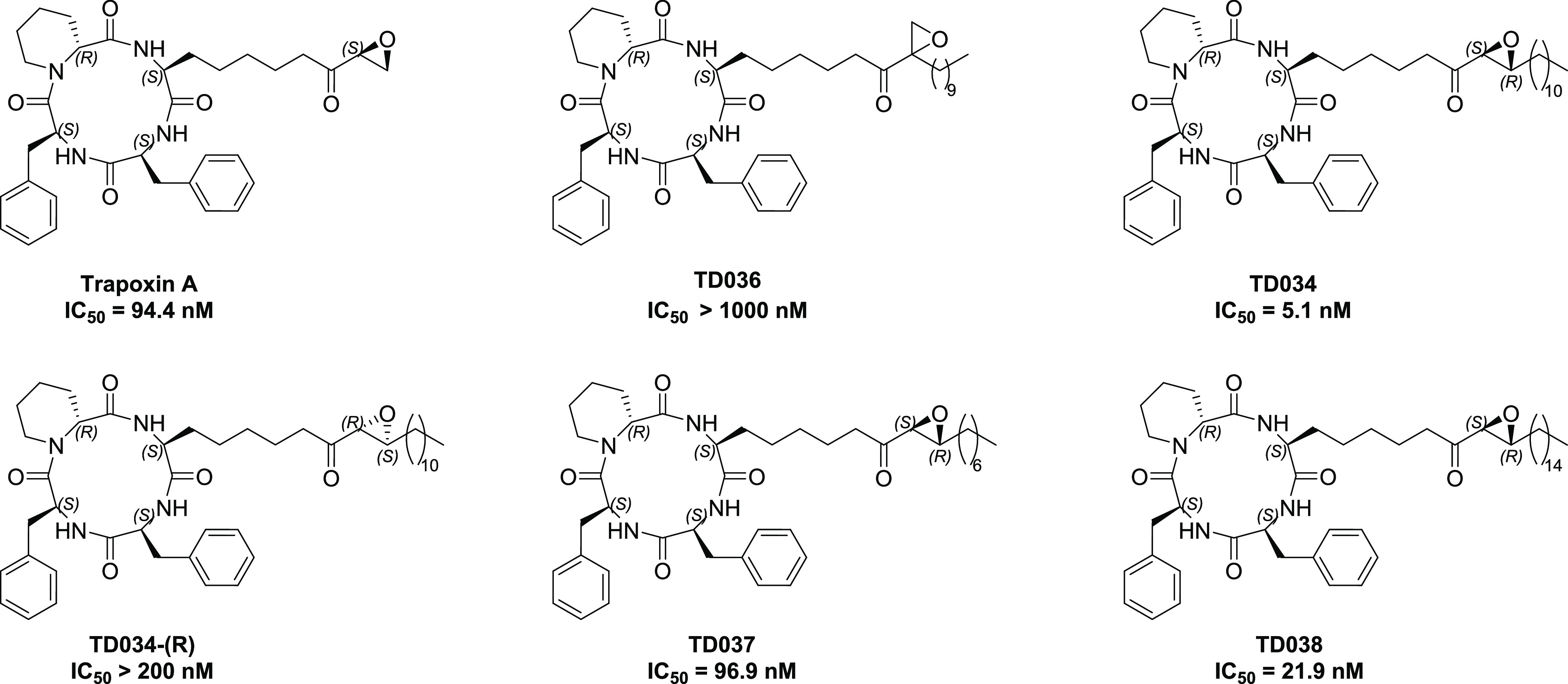
Trapoxin A analogues synthesized as HDAC11 inhibitors.

**Figure 3:**
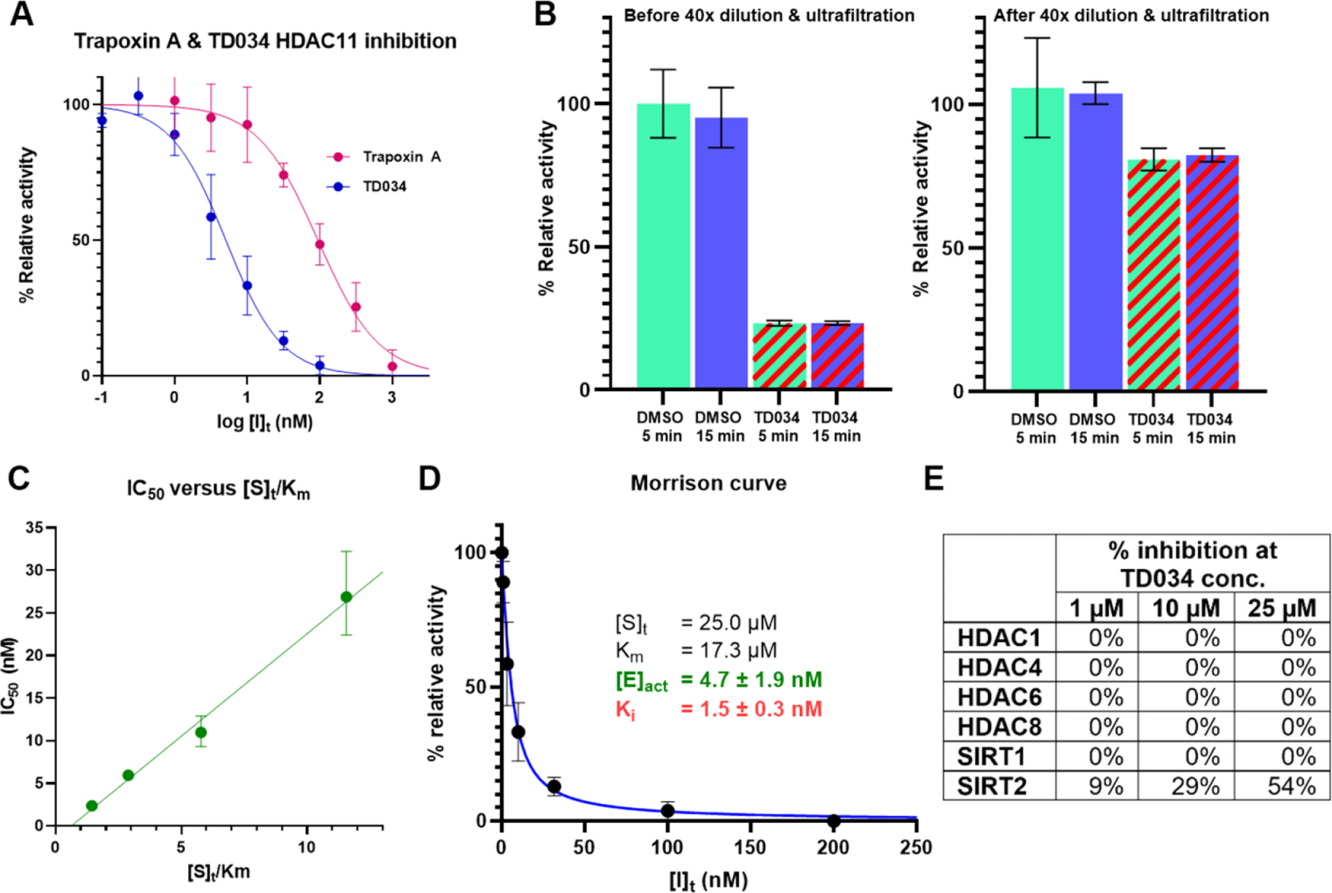
TD034 is a potent, selective, reversible HDAC11 inhibitor *in vitro*. Each measurement was performed in triplicate. (A) TD034 (IC_50_ = 5.1 ± 1.1 nM) is much more potent than trapoxin A (IC_50_ = 94.4 ± 22.4 nM). (B) TD034 inhibition of HDAC11 activity is reversible after 40× dilution and ultrafiltration. (C) TD034 is a competitive inhibitor. (D) Morrison curve for the TD034 inhibition of HDAC11: [E]_act_ and *K*_i_ were simultaneously determined by two-step nonlinear regression. (E) In enzymatic assays, TD034 does not significantly inhibit other HDACs/SIRTs.

The inhibitors synthesized are shown in Figure 2. Alkyl substitution at the α-position of epoxyketone (TD036) abolished HDAC11 inhibition, while substitution at the β-position with a C11-chain led to TD034, which has a nearly 20-fold increase in HDAC11 inhibition potency (IC_50_ = 5.1 ± 1.1 nM) compared to trapoxin A (IC_50_ = 94.4 ± 22.4 nM) (Figure 3A).

Changing the stereochemistry of the epoxide yielded the diastereomer TD034-(R) with diminished potency, indicating that the orientation of the epoxide is crucial for guiding the hydrophobic chain into the pocket. Varying the aliphatic chain length afforded inhibitors TD037 (C-7 chain) and TD038 (C-15 chain), which were less potent than TD034, perhaps due to a mismatch in chain length versus hydrophobic pocket depth. We also attempted to replace the Phe residues, but this led to a <5% yield of cyclic peptides due to the unfavorable entropy of head-to-tail tetrapeptide cyclization. These cyclizations were known to be very sensitive to residue interactions and stereochemistry.^19^ Thus, we decided to use TD034 for further investigation.

We next investigated the mode of inhibition of TD034. Previous studies indicated that trapoxin A is either a covalent^14^ or tight-binding reversible inhibitor of HDACs.^20^ First, we checked whether HDAC11 inhibition by TD034 is reversible. We incubated HDAC11 (15 nM) with either DMSO (as control) or TD034 (15 nM) for 5 or 15 min. Afterward, the samples were either used directly for activity assay, or diluted 40x with buffer, ultrafiltered with Amicon 30K to remove excess inhibitor, and then subjected to activity assay. The absolute activity of HDAC11 decreased 4-fold after ultrafiltration due to the instability of HDAC11 after prolonged dilution. Regardless, we found that the HDAC11 activity was recovered after dilution and ultrafiltration, and prolonged incubation time with TD034 did not affect the recovered activity of HDAC11 (Figure 3B), confirming that inhibition by TD034 is reversible. We then measured IC_50_ at different ratios of [S]_t_/*K*_m_ (Figure 3C). We found that the IC_50_ displayed a linear correlation with [S]_t_/*K*_m_, consistent with a competitive mechanism.^21^ Thus, we concluded that TD034 is a high-affinity, reversible, noncovalent inhibitor. Finally, we fitted dose–response data using a two-step nonlinear regression of the Morrison equation^22,23^ to simultaneously determine the active enzyme concentration ([E]_act_ = 4.7 ± 1.9 nM) and inhibition constant (*K*_i_ = 1.5 ± 0.3 nM) for TD034 (Figure 3D).

We screened TD034 against several other human HDACs and sirtuins. Interestingly, TD034 did not inhibit these HDACs or sirtuins, although it showed some potency against SIRT2 (IC_50_ ∼ 25 μM) (Figure 3E). SIRT2 is known to have efficient demyristoylase activity and possesses a large hydrophobic pocket, which could explain the inhibitory activity.^24^ Nonetheless, TD034 still exhibited >5000× selectivity for HDAC11 versus SIRT2.

### TD034 Inhibits HDAC11 Selectively in Cells

**Figure 4:**
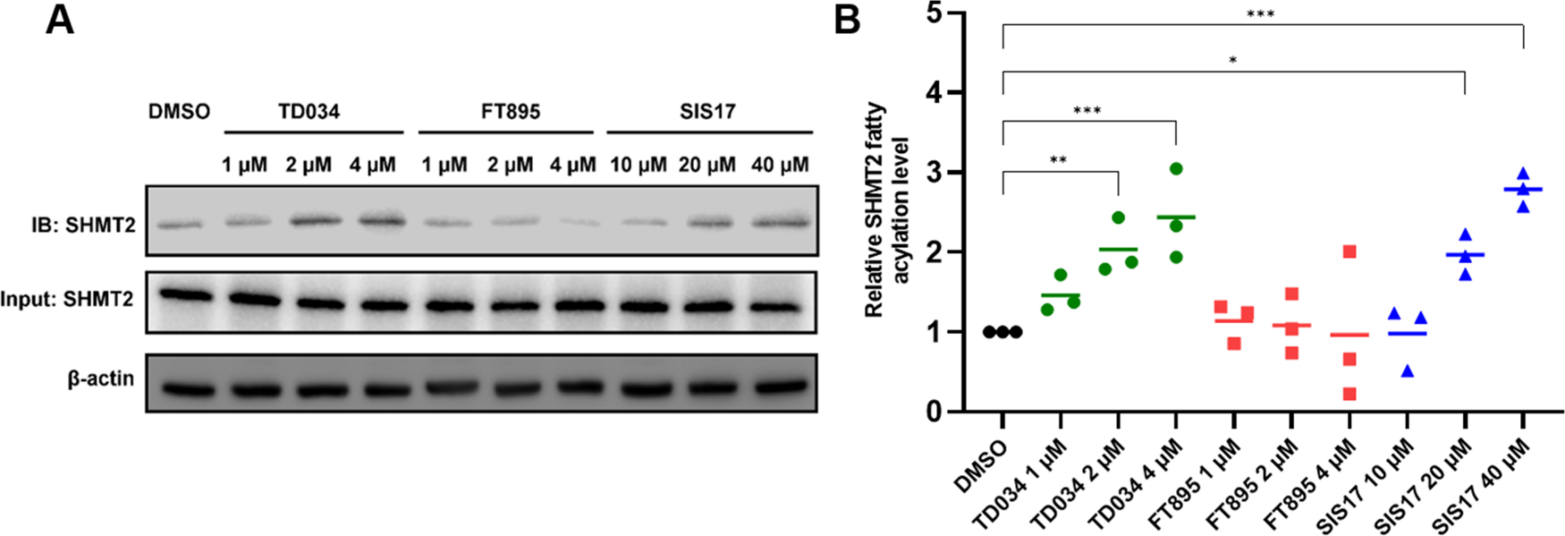
TD034 inhibits HDAC11 in HEK293T cells and leads to an elevated fatty acylation level of SHMT2. (A) Representative Western blot images showing the cellular SHMT2 acylation levels with different concentrations of TD034, FT895, and SIS17. (B) Quantification of SHMT2 fatty acylation levels. *P < 0.05, **P < 0.01, ***P < 0.001.

With this encouraging data, we then tested TD034 in HEK293T to check whether it could inhibit HDAC11 selectively in cells. SHMT2 was reported as a defatty-acylation substrate of HDAC11.^5^ Thus, we tested whether TD034 could inhibit HDAC11 and increase the fatty acylation level of endogenous SHMT2. We treated HEK293T cells with an alkyne-tagged myristic acid analog, Alk14, along with the inhibitors (TD034, SIS17, or FT895) for 3 h. Click chemistry was performed on cell lysate with Biotin-azide, followed by Streptavidin pulldown. The amount of labeled SHMT2 was then detected by Western blot (Figure 4A). TD034 significantly increased the fatty acylation level of SHMT2 at 2 μM (Figure 4B). The same effect was observed at 20 μM for SIS17, while FT895 at 4 μM had no statistically significant effect, consistent with a previous report.^13^ We noted that higher concentrations of TD034 are needed to inhibit HDAC11 in cells than in the *in vitro* enzymatic assay, likely due to unfavorable membrane partitioning of the alkyl chain.

**Figure 5:**
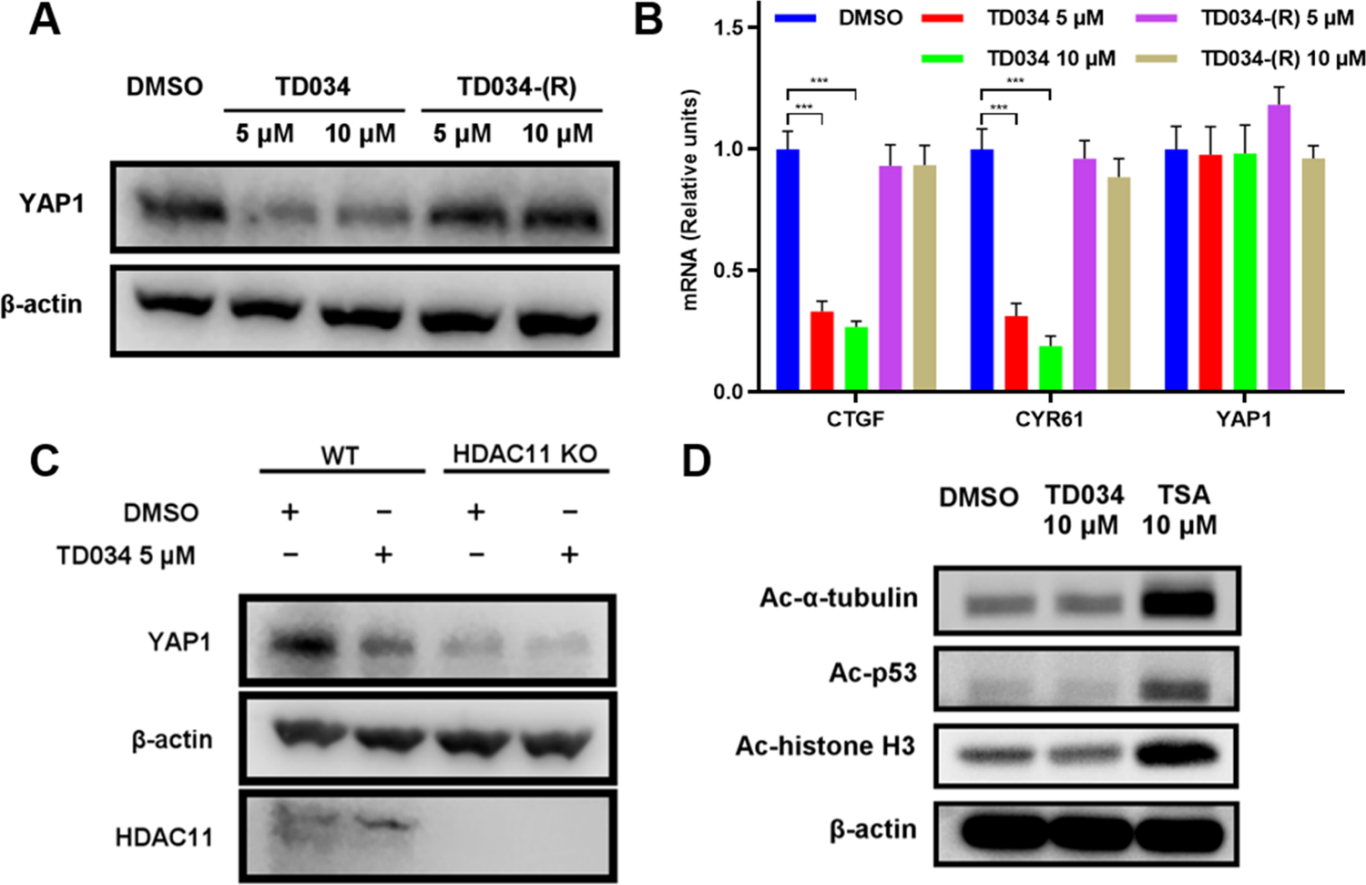
TD034 selectively inhibits HDAC11 and leads to YAP1 protein level decrease in A549 cells. (A) TD034, but not the less active TD034-(R), decreased the YAP1 protein level. (B) TD034, but not TD034-(R), decreased the mRNA level of YAP1 target genes. YAP1 mRNA level was not affected by TD034. (C) TD034 treatment decreased the YAP1 protein level in WT but not in HDAC11 KO cells. (D) TD034 does not inhibit class I HDACs, HDAC6, SIRT1/2 in cells, as measured by α-tubulin, p53, and histone H3 acetylation levels. *P < 0.05, **P < 0.01, ***P < 0.001.

HDAC11 expression is upregulated in lung cancer and is associated with poor prognosis in lung cancer patients. Consistent with the previous finding that depletion of HDAC11 downregulated YAP1 (yes-associated protein 1) protein expression in lung cancer cells,^8^ A549 cells treated with TD034 resulted in a significant reduction of YAP1 protein levels (Figure 5A) and a decrease in the mRNA levels of two YAP1 target genes, CTGF and CYR61 (Figure 5B). Using TD034-(R), a much less potent analogue, we did not observe such an effect.

To confirm whether the downregulation of YAP1 was due to the inhibition of HDAC11 by TD034, we tested TD034 on both wild-type (WT) and HDAC11 knockout (KO) A549 cells. First, we found that without the TD034 treatment, the endogenous protein level of YAP1 in HDAC11 KO cells was lower than that in WT cells. Second, treatment with TD034 led to a reduced YAP1 protein level in WT cells but not in HDAC11 KO cells (Figure 5C). These results confirmed that TD034 decreases the YAP1 level via HDAC11 inhibition and extended the potential of using TD034 to manipulate the HDAC11-mediated hippo-YAP signaling pathway.

To demonstrate that TD034 is selective toward HDAC11 in cells, we measured the acetylation levels of α-tubulin, p53, and histone H3 by Western blot. As a positive control, we used trichostatin A (TSA), a nonselective HDAC inhibitor against both class I and class IIb HDACs.^25^ After 3 h of treatment, the TSA-treated cell had an elevated level of acetylated α-tubulin (HDAC6 and SIRT2 target) and increased levels of acetylated histone H3 and acetylated p53 (class I HDACs and SIRT1 target).^26^ Meanwhile, the TD034-treated cells did not have such an effect (Figure 5D). Thus, TD034 is selective for HDAC11 and does not inhibit other HDACs at the concentration tested, consistent with the *in vitro* activity assay results.

## Conclusions

In summary, by modifying trapoxin A, we have developed TD034, a highly potent HDAC11 selective inhibitor. TD034 inhibits HDAC11 at low nanomolar concentrations in enzymatic assays *in vitro* and low micromolar concentrations in cells, making it more potent than previously discovered inhibitors. Furthermore, TD034 selectively inhibits HDAC11 in cells. TD034 is a great HDAC11 inhibitor candidate for further optimization and biological applications.

## Methods

### Chemical Syntheses

Detailed synthesis procedures and characterization of compounds **2–12** are provided in the Supporting Information.

### Data Processing

All quantified measurements were performed in triplicates. Data processing was performed using Graphpad Prism 9.5.0. Repeated measures of one-way ANOVA with Fisher’s LSD test were used to determine the *P* value. The dose–response data was fitted using a two-step nonlinear regression of the Morrison equation. In the first step, an estimated [E]_act_ = 7.6 nM (determined by linear extrapolation of Zone A)^22^ was held constant for regression to yield an estimated *K*_i_ = 0.85 nM. In the second step, both [E]_act_ and *K*_i_ were treated as variables, using previous estimates as initial values. The best-fit curve (*R*^2^ = 0.99) yields [E]_act_ = 4.7 ± 1.9 nM and *K*_i_ = 1.5 ± 0.3 nM for TD034.

### HDAC Enzyme Activity Assays

HDACs and SIRTs were expressed and purified as previously described.^13^ The HDAC11 concentration used in the experiments was estimated to be 74 nM by SDS-PAGE gel; active HDAC11 concentration was estimated by Morrison curve fitting to be 4.7 ± 1.9 nM. Not all HDAC11 enzyme was active due to post-translation modifications, as well as denaturation during purification, storage, and handling. For the HDAC11 activity assay, Myr-H3K9 peptide (25 μM), HDAC11 (4.7 nM), and inhibitors at various concentrations were incubated in 20 μL of assay buffer (50 mM Tris/Cl, pH 8.0, 137 mM NaCl, 2.7 mM KCl, 1 mM MgCl_2_) at 37 °C. For HDAC4, trifluoroacetyl-H3K9 was used as a substrate. For HDAC1, 6, 8, and SIRT1-2, Ac-H3K9 was used as a substrate. For SIRT1/2, the assay buffer includes 1 mM DTT and 1 mM NAD^+^.^13^ TD034 is relatively stable in Tris buffer and DTT (Figure S2, Supporting Information). The reaction was conducted for 15 min (HDAC11), 30 min (HDAC1, 4, 6, 8), and 5 min (SIRT1-2). Then, 20 μL of 0.2% TFA/acetonitrile was added to quench the reaction. The samples were analyzed by HPLC using a Chromolith HighResolution RP-18 end-capped 100 mm × 4.6 mm column (EMD Millipore). Mobile phase A was 0.1% TFA in water and mobile phase B was 0.1% TFA in acetonitrile. The total flow rate was 1 mL/min, and the gradient was 0% B (2 min), 0–60% B (7 min), 100% B (4 min), and 0% B (2 min). The relative ratio of product/substrate in each sample was compared to control sample (no inhibitor) to determine the inhibition level.

### HDAC11 Enzyme Kinetic Assays

For preincubation assay, TD034 (15 nM) or DMSO (control) was incubated with HDAC11 (15 nM) for 5 or 15 min in 10 μL of assay buffer (50 mM Tris–Cl, pH 8.0, 137 mM NaCl, 2.7 mM KCl, 1 mM MgCl_2_) at 37 °C. Afterward, either (i) 10 μL of the Myr-H3K9 peptide (50 μM) was added, or (ii) the solution was diluted with 390 μL of assay buffer, concentrated by Amicon 30K filters until ∼20 μL remained. Then, the Myr-H3K9 peptide (25 μM) was added. The samples were incubated at 37 °C for 15 min. Each reaction was quenched with 20 μL of 0.2% TFA/acetonitrile, and the samples were analyzed as described above. For IC_50_ versus [S]_t_/*K*_m_ assay, the Myr-H3K9 peptide (200, 100, 50, 25 μM), HDAC11 (4.7 nM), and inhibitors at various concentrations were incubated in 20 μL of assay buffer (50 mM Tris/Cl, pH 8.0, 137 mM NaCl, 2.7 mM KCl, 1 mM MgCl_2_) at 37 °C for 15 min. Each reaction was quenched with 20 μL of 0.2% TFA/acetonitrile, and the samples were analyzed as described above.

### HDAC11 in Cell Assay: Defatty Acylation of SHMT2

HEK293T in a six-well plate at 80% confluency was treated with 50 μM Alk14 and inhibitors at various concentrations. The cells were incubated for 3 h. The cells were harvested and lysed in 200 μL of 4% SDS lysis buffer (50 mM triethanolamine, 150 mM NaCl, 4% SDS, pH 7.4) with a 1:100 protease inhibitor cocktail and 1:1000 nuclease for 15 min. The cell lysates were then diluted with 3.8 mL of HEPES buffer (50 mM HEPES, 150 mM NaCl, 1% NP-40, pH 7.4), and then concentrated using Amicon Ultra-4 (30 kDa cutoff) for 45 min at 4000*g*. The retained samples were then diluted to 0.5 mL with HEPES buffer, followed by the addition of Biotin-N_3_ (5 μL, 5 mM in DMF), TBTA (5 μL, 2 mM in DMF), CuSO_4_ (5 μL, 50 mM in water), and TCEP (5 μL, 50 mM in water). The samples were shaken at 37 °C for 1 h, then diluted with 3 mL of HEPES buffer, and concentrated again using Amicon Ultra-4 (30 kDa cutoff) for 45 min at 4000*g*. The retained samples were then diluted to 0.5 mL with HEPES buffer, followed by the addition of 20 μg of magnetic streptavidin beads (prewashed with the HEPES buffer). The mixture was shaken for 1 h and the supernatant was removed. Hydroxylamine in the HEPES buffer (100 μL, 0.5 M) was added, and the mixture was then shaken for 30 min. The supernatant was removed, and the beads were washed with HEPES buffer (2 × 500 μL). The remaining beads were incubated at 95 °C with 40 μL of 4% SDS lysis buffer and 8 μL of 6× loading buffer for 10 min. The eluants were further analyzed by SDS-PAGE and Western blot for SHMT2.

### YAP1 Protein Level and Target Genes mRNA Level Determination

A549 cells were treated with TD034 at 5 and 10 μM for 24 hr. Western blot was used to check for YAP1 protein level. qRT-PCR was used to check for YAP1 downstream genes transcription (CTGF and CYR61). Total RNA was extracted using IBI Isolate Total Extraction Reagent Kit (IB47602). Two milligrams of RNA was reverse transcribed using OneScript Plus cDNA Synthesis Kit (ABM G236) according to the manufacturer’s protocol. Real-time PCR was performed using BlasTaq 2X qPCR MasterMix (ABM G892) on a QuantStudio 3 Real-Time PCR System. All qPCR reactions were performed in triplicates. The list of primers is included in the Supporting Information.

## Supporting Information Available

Reagents, instruments, primers for qRT-PCR, activity assay data for TD037, TD038, and TD034(R), synthetic methods, and NMR spectra of important compounds (PDF)
