# Supplementary material for: Trapoxin A Analogue as a Selective Nanomolar Inhibitor of HDAC11
Source: ACS Chem Biol. 2023 Mar 28;18(4):803–9. doi: 10.1021/acschembio.2c00840 (PMC10127203; doi:10.1021/acschembio.2c00840)
Supplement: Supplementary file 1 — cb2c00840_si_001.pdf [file cb2c00840_si_001.pdf]

SUPPORTING INFORMATION

A Trapoxin A analog as a selective nanomolar inhibitor of HDAC11

Thanh Tu Ho[a], Changmin Peng[b], Edward Seto[b], Hening Lin[a,c,*]

[a] Department of Chemistry and Chemical Biology, Cornell University, Ithaca, NY 14853, USA.

[b] Department of Biochemistry & Molecular Medicine, School of Medicine & Health Sciences, George Washington Cancer Center, George Washington University, Washington, DC, 20037, USA

[c] Howard Hughes Medical Institute, Cornell University, Ithaca, NY 14853, USA

* Corresponding author. Email: hl379@cornell.edu

REAGENTS. Chemical reagents and solvents were purchased from Sigma-Aldrich, Fisher Scientific, Combi-blocks, Chem-Impex, and were used as received unless otherwise noted. Anti-FLAG affinity gel (A2220), 3X FLAG peptide (F4799), Amicon Ultra-4 (30 kDa cutoff), antibody against β-actin (A2228) were purchased from Sigma Aldrich. Trapoxin A (sc-253730), antibodies against SHMT2 (sc-390641 HRP), acetyl-α-tubulin (sc-23950 HRP), GAPDH (sc-47724 HRP) were purchased from Santa Cruz Biotechnology. Antibodies against Acetyl-histone H3 (9675), YAP (4912) and HDAC11 (58442) were purchased from Cell Signaling Technologies. Streptavidin magnetic beads, universal nuclease for cell lysis were purchased from Thermo Scientific. Clarity MAX western blot visualizing reagent was purchased from BioRad.

Modified and unmodified H3K9 peptide were purchased from Biomatik. The sequences are as follows:

H3K9 unmodified:	KQTARKSTGGWW
Ac-H3K9:	KQTARK(Ac)STGGWW
Myr-H3K9:	KQTARK(Myr)STGGWW

INSTRUMENT. Analytical thin layer chromatography (TLC) was performed on precoated glass-backed silica gel 60 F254 plates (EMD Millipore), visualized by UV 254 nm, or by potassium permanganate ($KMnO_4$), phosphomolybdic acid (PMA) or 2,4-dinitrophenylhydrazine (DNP) staining. Silica gel flash chromatography was performed on a Teledyne ISCO CombiFlash Nextgen+.

Analytical high performance liquid chromatography (HPLC) was carried out on a Shimadzu LC-2020. Mobile phase A: 0.1% trifluoroacetic acid (TFA) in water. Mobile phase B: 0.1% trifluoroacetic acid (TFA) in acetonitrile.

^{1}H and ^{13}C nuclear magnetic resonance (NMR) spectra were recorded on a Bruker AVIII-500 (500 MHz). Chemical shifts (δ) were reported in parts per million (ppm) and referenced to TMS (δ = 0). Coupling constant (J) were reported in hertz (Hz), and multiplicities were listed as follows: br = broad, s = singlet, d = doublet, t = triplet, q = quartet, m = multiplet.

Accurate mass was recorded on a Thermo Fisher Q Exactive Orbitrap with Direct Analysis in Real Time (DART) ion source, using dioctyl adipate, calcd. for $C_{22}H_{43}O_4^+$ $[M+H]^+$ 371.3156 as the lockmass.

PRIMERS FOR qRT-PCR

ACTIN Forward:	ATCACCATTGGCAATGAGCG
ACTIN Reverse:	TTGAAGGTAGTTTCGTGGAT
CTGF Forward:	ACCGACTGGAAGACACGTTTG
CTGF Reverse:	CCAGGTCAGCTTCGCAAGG
CYR61 Forward:	GGTCAAAGTTACCGGGCAGT
CYR61 Reverse:	GGAGGCATCGAATCCCAGC
YAP Forward:	TAGCCCTGCGTAGCCAGTTA
YAP Reverse:	TCATGCTTAGTCCACTGTCTGT

HDAC11 CRISPR gRNA SEQUENCE

TGATGGACAGCCCCCCAAGG

ACTIVITY ASSAY DATA FOR OTHER INHIBITORS

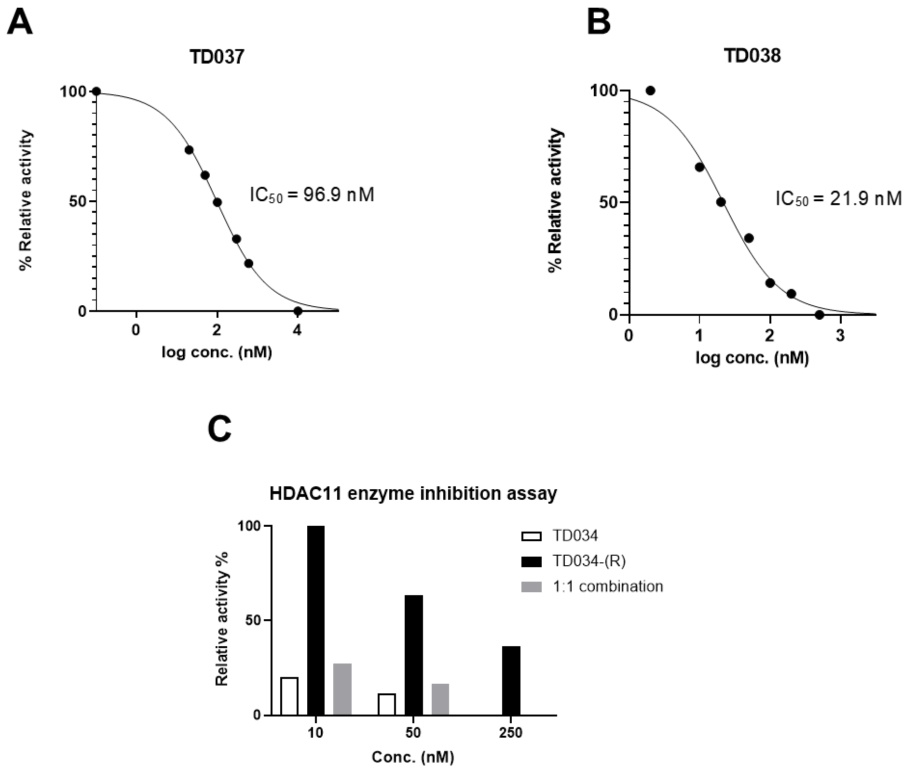

Figure S1: A,B) IC_{50} curve for TD037 & TD038; C) TD034-(R) is less potent than TD034

STABILITY OF TD034 UNDER ASSAY CONDITION

A

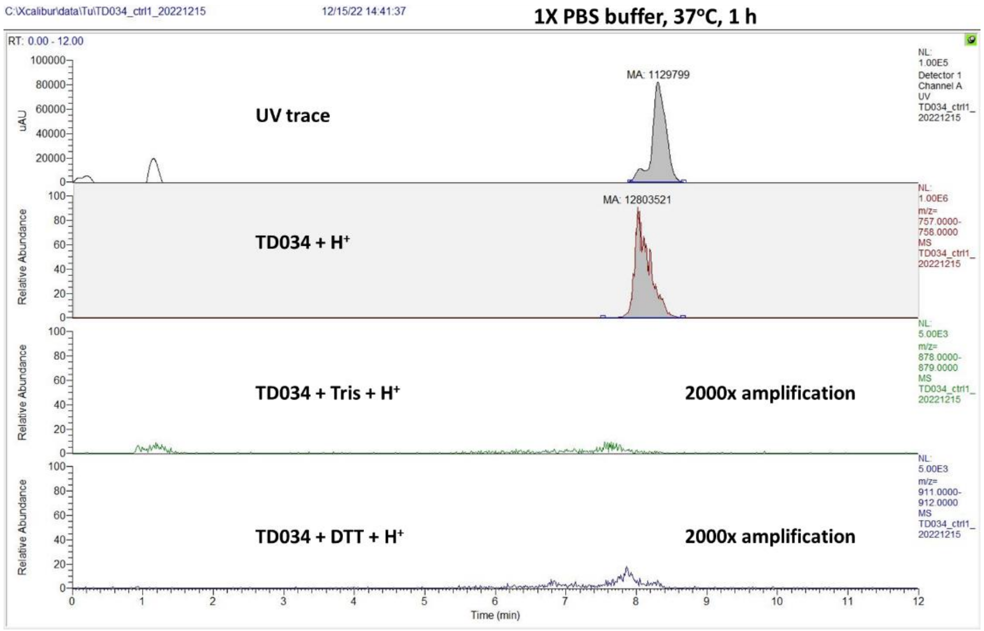

B

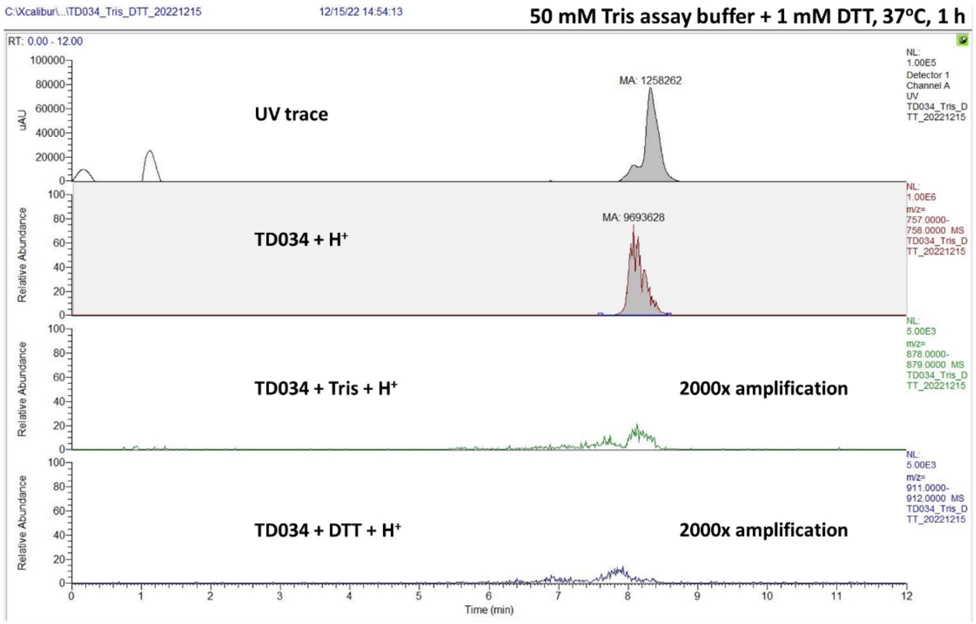

Figure S2: TD034 is relatively stable in the presence of Tris & DTT.

A) TD034 (100 µM) in PBS buffer (pH 7.4, 137 mM NaCl, 2.7 mM KCl, 4.3 mM Na2HPO4, 1.47 mM KH2PO4).
B) TD034 (100 µM) in assay buffer with DTT (pH 8.0, 50 mM Tris/Cl, 137 mM NaCl, 2.7 mM KCl, 1 mM MgCl2, 1 mM DTT).

GENERAL SYNTHESES OF EPOXYKETONE MOTIFS

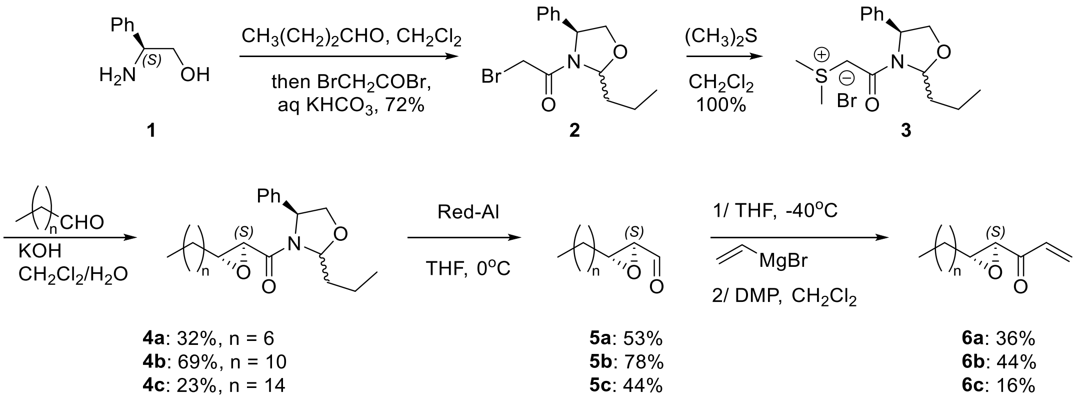

Scheme S1

Synthesis of 2: To a solution of (S)-phenylglycinol (6.86 g, 50 mmol) in CH_2Cl_2 (200 mL) was added butyraldehyde (9 mL, 100 mmol). The cloudy solution was stirred for 30 min, then 5% aqueous $KHCO_3$ was added (200 mL) with vigorous stirring. Bromoacetyl bromide (50 mmol, 4.36 mL) was added dropwise, then the 2-phase mixture was vigrorously stirred for 1 h. The phases were then separated, and the aqueous was extracted with CH_2Cl_2 (50 mL). The combined organic phase was dried with Na_2SO_4, filtered, and then evaporated. Flash chromatography (0 - 25% ethyl acetate/hexane) afforded the product **2** as a colourless oil (11.3 g, 72%). ^1H NMR (500 MHz, $CDCl_3$) δ 7.47 – 7.29 (m, 5H), 5.42 (dd, *J* = 9.1, 2.5 Hz, 1H), 5.08 (t, *J* = 6.0 Hz, 1H), 4.35 (dd, *J* = 9.0, 6.9 Hz, 1H), 4.00 (dd, *J* = 9.1, 5.2 Hz, 1H), 3.53 (d, *J* = 2.8 Hz, 2H), 2.26 – 2.14 (m, 1H), 1.73 (dtd, *J* = 14.4, 9.4, 5.4 Hz, 1H), 1.64 – 1.47 (m, 2H), 1.01 (t, *J* = 7.4 Hz, 3H). ^{13}C NMR (126 MHz, $CDCl_3$) δ 165.10, 139.05, 129.37, 128.57, 126.12, 92.02, 73.77, 60.79, 35.25, 27.96, 18.61, 13.90. ^{1}HRMS (DART) calcd. for $C_{14}H_{19}BrNO_2^+$ $[M+H]^+$ 312.0594, found 312.0596.

Synthesis of 3: To a solution of **2** (11.3 g, 36.2 mmol) in CH_2Cl_2 (20 mL) was added dimethylsulfide (5.36 mL, 72.4 mmol). The solution was vigorously stirred for 4 hours, then evaporated to afford product **3** as a white solid (13.6 g, 100%). ^1H NMR (500 MHz, $CDCl_3$) δ 7.43 (d, *J* = 22.3 Hz, 4H), 7.34 – 7.30 (m, 1H), 6.36 (d, *J* = 16.1 Hz, 1H), 5.60 (dd, *J* = 6.7, 4.2 Hz, 1H), 5.36 (dd, *J* = 9.1, 2.4 Hz, 1H), 4.39 (dd, *J* = 9.0, 6.8 Hz, 1H), 4.00 (dd, *J* = 9.1, 4.2 Hz, 1H), 3.73 (d, *J* = 16.0 Hz, 1H), 3.37 – 3.17 (m, 6H), 2.16 (dddd, *J* = 13.0, 9.9, 6.1, 2.2 Hz, 1H), 1.72 (dtd, *J* = 14.1, 9.5, 5.1 Hz, 1H), 1.62 – 1.49 (m, 2H), 1.01 (t, *J* = 7.4 Hz, 3H). ^{13}C NMR (126 MHz, $CDCl_3$) δ 162.23, 138.68, 129.42, 129.35, 128.86, 128.54, 126.70, 126.30, 126.09, 91.79, 73.95, 60.53, 48.17, 35.15, 25.44, 25.10, 18.50, 18.10, 13.93, 13.87. HRMS (DART) calcd. for $C_{15}H_{22}NO_2S^+$ $[M-CH_3Br+H]^+$ 280.1366, found 280.1363.

General synthesis of 4: To a mixture of **3** in 1:1 CH_2Cl_2:water (1.0 M) was added KOH (1.1 eq.), and the mixture was vigorously stirred for 1 min. Aliphatic aldehyde (1.5 eq) was added, and the mixture was vigorously stirred overnight. The phases were separated, and the aqueous phase was extracted with CH_2Cl_2 (5 mL/mmol). The combined organic phase was dried with Na_2SO_4, filtered, and then evaporated. Flash chromatography (0 - 25% ethyl acetate/hexane) afforded products **4a-c**.

4a: 3.2 g, 32%. ^1H NMR (500 MHz, $CDCl_3$) δ 7.45 – 7.29 (m, 5H), 5.51 (dd, *J* = 9.3, 2.7 Hz, 1H), 5.15 (t, *J* = 6.5 Hz, 1H), 4.43 – 4.35 (m, 1H), 3.97 (dd, *J* = 8.8, 5.7 Hz, 1H), 3.00 – 2.91 (m, 2H), 2.23 – 2.13 (m, 1H), 1.72 (ddd, *J* = 21.1, 10.9, 6.9 Hz, 1H), 1.54 (dq, *J* = 16.3, 8.7 Hz, 3H), 1.28 – 1.00 (m, 14H), 0.87 (dd, *J* = 8.2, 6.1 Hz, 3H). ^{13}C NMR (126 MHz, $CDCl_3$) δ 166.65, 139.83, 129.27, 129.24, 128.34, 126.05, 91.75, 73.76, 60.36, 58.65, 53.62, 35.14, 31.71, 31.66, 30.86, 29.10, 29.07, 28.99, 25.17, 22.61, 18.58, 14.08, 13.92. HRMS (DART) calcd. for $C_{22}H_{34}NO_3^+$ $[M+H]^+$ 360.2533, found 360.2531.

4b: 10.37 g, 69%. ^1H NMR (500 MHz, $CDCl_3$) δ 7.46 – 7.28 (m, 5H), 5.51 (dd, J = 9.2, 2.5 Hz, 1H), 5.15 (t, J = 6.4 Hz, 1H), 4.39 (dd, J = 9.0, 7.0 Hz, 1H), 3.97 (dd, J = 9.1, 5.9 Hz, 1H), 3.00 – 2.92 (m, 2H), 2.18 (dddd, J = 13.1, 9.6, 6.2, 2.5 Hz, 1H), 1.72 (dtd, J = 14.3, 9.5, 5.4 Hz, 1H), 1.61 – 1.48 (m, 3H), 1.30 – 1.00 (m, 22H), 0.88 (t, J = 6.9 Hz, 3H). ^{13}C NMR (126 MHz, $CDCl_3$) δ 166.65, 139.84, 129.27, 128.34, 126.05, 91.76, 73.76, 60.37, 58.65, 53.64, 35.15, 31.93, 30.88, 29.62, 29.47, 29.35, 29.13, 25.19, 22.70, 18.58, 14.14, 13.91. HRMS (DART) calcd. for $C_{26}H_{42}NO_3^+$ [M+H]$^+$ 416.3159, found 416.3154.

4c: 2.97 g, 23%. ^1H NMR (500 MHz, $CDCl_3$) δ 7.44 – 7.31 (m, 5H), 5.51 (d, J = 9.2 Hz, 1H), 5.15 (t, J = 6.6 Hz, 1H), 4.39 (td, J = 7.9, 1.8 Hz, 1H), 3.97 (t, J = 7.6 Hz, 1H), 3.00 – 2.92 (m, 2H), 2.18 (dtd, J = 13.6, 6.9, 3.7 Hz, 1H), 1.72 (dhept, J = 14.7, 4.9 Hz, 1H), 1.54 (td, J = 15.1, 7.5 Hz, 3H), 1.28 – 1.01 (m, 30H), 0.88 (td, J = 6.4, 3.6 Hz, 3H). ^{13}C NMR (126 MHz, $CDCl_3$) δ 166.65, 139.83, 129.28, 128.35, 126.05, 91.76, 73.76, 60.37, 58.66, 53.63, 35.15, 31.94, 30.88, 29.72, 29.71, 29.68, 29.63, 29.48, 29.38, 29.36, 29.13, 25.19, 22.71, 18.58, 14.15, 13.92. HRMS (DART) calcd. for $C_{30}H_{50}NO_3^+$ [M+H]$^+$ 472.3785, found 472.3783.

General synthesis of 5: To a solution of **4a-c** in THF (0.1 M) at 0 °C was added dropwise Red-Al (1.5 eq., 3.5 M in toluene), then the solution was stirred for 15 min. The reaction was quenched carefully with 1M aqueous HCl (5 eq.), then diluted with CH_2Cl_2 (20 mL/mmol). The phases were separated, and the aqueous phase was extracted with CH_2Cl_2 (5 mL/mmol). The combined organic phase was dried with Na_2SO_4, filtered then evaporated. Flash chromatography (0 - 10% ethyl acetate/hexane) afforded products **5a-c**.

5a: 810 mg, 53%. Highly unstable. ^1H NMR (500 MHz, $CDCl_3$) δ 9.02 (dd, J = 6.2, 1.7 Hz, 1H), 3.30 – 2.77 (m, 2H), 1.69 – 1.27 (m, 12H), 0.89 (t, J = 6.7 Hz, 3H). ^{13}C NMR (126 MHz, $CDCl_3$) δ 198.57, 59.19, 56.83, 31.70, 31.22, 29.20, 29.10, 25.79, 22.62, 14.08. HRMS (DART) calcd. for $C_{10}H_{19}O_2^+$ [M+H]$^+$ 171.1380, found 171.1381.

5b: 4.43 g, 78%. ^1H NMR (500 MHz, $CDCl_3$) δ 9.02 (d, J = 6.3 Hz, 1H), 3.26 – 3.20 (m, 1H), 3.13 (dd, J = 6.3, 1.9 Hz, 1H), 1.72 – 1.58 (m, 2H), 1.47 (qt, J = 12.8, 4.0 Hz, 2H), 1.27 (d, J = 10.0 Hz, 16H), 0.88 (t, J = 6.8 Hz, 3H). ^{13}C NMR (126 MHz, $CDCl_3$) δ 198.54, 59.19, 56.82, 31.92, 31.22, 29.61, 29.49, 29.44, 29.34, 29.24, 25.78, 22.69, 14.13. HRMS (DART) calcd. for $C_{14}H_{27}O_2^+$ [M+H]$^+$ 227.2006, found 227.2005.

5c: 786 mg, 44%. ^1H NMR (500 MHz, $CDCl_3$) δ 9.02 (dd, J = 6.5, 2.1 Hz, 1H), 3.22 (d, J = 5.8 Hz, 1H), 3.13 (dd, J = 6.3, 2.4 Hz, 1H), 1.66 (qt, J = 13.6, 6.9 Hz, 2H), 1.57 – 1.40 (m, 3H), 1.26 (s, 23H), 0.88 (td, J = 7.0, 2.3 Hz, 3H). ^{13}C NMR (126 MHz, $CDCl_3$) δ 198.56, 59.19, 56.83, 31.94, 31.23, 29.71, 29.69, 29.67, 29.66, 29.62, 29.50, 29.44, 29.38, 29.25, 25.79, 22.71, 14.15. HRMS (DART) calcd. for $C_{18}H_{35}O_2^+$ [M+H]$^+$ 283.2632, found 283.2631.

General synthesis of 6: To a solution of **5a-c** in THF (0.1 M) at -40 °C was added dropwise vinylmagnesium bromide (1.25 eq., 1.0 M in THF), then the solution was stirred for 1 h. The reaction was quenched with saturated NH_4Cl solution (5 eq.), warmed to room temperature, then diluted with CH_2Cl_2 (20 mL/mmol). The phases were separated, and the aqueous phase was extracted with CH_2Cl_2 (5 mL/mmol). The combined organic phase was dried with Na_2SO_4, filtered then evaporated. Flash chromatography (0 - 25% EA:Hexane) afforded a mixture of epimeric alcohols, which was used directly in the next step. The alcohols were dissolved in CH_2Cl_2 (0.5 M), then $NaHCO_3$ (6 eq.) and Dess-Martin periodinane (2 eq.) were added. The mixture was vigorously stirred for 15 min, then H_2O (2 eq.) was added. The mixture was vigorously stirred for another 15 min, then diluted with hexane (20 mL/mmol). The mixture was filtered through a pad silica gel, washing with 1:4 ethyl acetate/hexane (10 mL/mmol), and the combined organic phase was evaporated to afford product **6a-c**.

6a: 337 mg, 36%. ^1H NMR (500 MHz, $CDCl_3$) δ 6.55 – 6.41 (m, 2H), 5.84 (dd, J = 9.8, 2.6 Hz, 1H), 3.40 (d, J = 2.3 Hz, 1H), 3.08 (t, J = 5.7 Hz, 1H), 1.71 – 1.61 (m, 2H), 1.53 – 1.40 (m, J = 6.7 Hz, 2H), 1.31 (dtd, J = 25.1, 12.9, 8.3 Hz, 8H), 0.88 (t, J = 6.5 Hz, 3H). ^{13}C NMR (126 MHz, $CDCl_3$) δ 196.00, 130.58, 130.40, 58.91, 58.40, 31.80, 31.71, 29.23, 29.13, 25.81, 22.62, 14.09. HRMS (DART) calcd. for $C_{12}H_{21}O_2^+$ [M+H]$^+$ 197.1536, found 197.1538.

6b: 2.17 g, 44%. ^{1}H NMR (500 MHz, $CDCl_3$) δ 6.58 – 6.43 (m, 2H), 5.86 (dd, *J* = 9.7, 2.4 Hz, 1H), 3.42 (d, *J* = 1.9 Hz, 1H), 3.10 (ddd, *J* = 5.9, 5.0, 2.0 Hz, 1H), 1.75 – 1.60 (m, 2H), 1.57 – 1.42 (m, 2H), 1.38 – 1.27 (m, 16H), 0.90 (t, *J* = 6.9 Hz, 3H). ^{13}C NMR (126 MHz, $CDCl_3$) δ 195.98, 130.55, 130.41, 58.92, 58.39, 31.92, 31.80, 29.62, 29.50, 29.46, 29.34, 29.27, 25.80, 22.70, 14.13. HRMS (DART) calcd. for $C_{16}H_{29}O_2^+$ $[M+H]^+$ 253.2162, found 253.2161.

6c: 136 mg, 16%. ^{1}H NMR (500 MHz, $CDCl_3$) δ 6.56 – 6.42 (m, 2H), 5.85 (dd, *J* = 9.7, 2.8 Hz, 1H), 3.40 (s, 1H), 3.08 (dq, *J* = 6.3, 2.9 Hz, 1H), 1.72 – 1.59 (m, 2H), 1.55 – 1.41 (m, 3H), 1.26 (s, 23H), 0.88 (td, *J* = 7.1, 2.7 Hz, 3H). ^{13}C NMR (126 MHz, $CDCl_3$) δ 196.01, 130.58, 130.41, 58.92, 58.41, 31.94, 31.81, 29.71, 29.67, 29.63, 29.51, 29.47, 29.38, 29.28, 25.81, 22.71, 14.15. HRMS (DART) calcd. for $C_{20}H_{37}O_2^+$ $[M+H]^+$ 309.2788, found 309.2789.

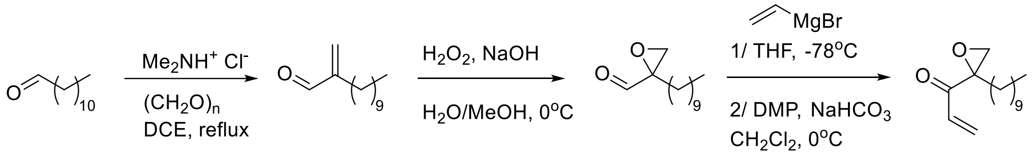

Scheme S2

Synthesis of S2-4: A solution of dodecanal (222 µL, 1 mmol), paraformaldehyde (300 mg, 10 mmol), dimethylamine hydrochloride (407 mg, 5 mmol) in DCE (5 mL) was refluxed for 3 h. The solution was diluted with hexane (20 mL) then filter through a silica plug. The eluant was evaporated to afford crude **S2-2** (80 mg), which was dissolved in MeOH (2 mL). Hydrogen peroxide 30% (82 µL, 0.8 mmol) and 5 M aqueous NaOH (16 µL) was premixed, then added to the solution at 0 °C. The solution was stirred for 30 min, then quenched with saturated aqueous $Na_2S_2O_5$ (5 mL). The mixture was extracted with CH_2Cl_2 (3 x 10 mL), and the combined organic phase was dried with Na_2SO_4, filtered then evaporated to afford crude **S2-3**, which was dissolved in THF (2 mL) at -78 °C. Vinylmagnesium bromide (0.5 mL, 1 M in THF) was added, then the solution was stirred for 1 h. The reaction was quenched with saturated aqueous NH_4Cl (5 mL), then warmed to room temperature. The mixture was extracted with CH_2Cl_2 (3 x 10 mL), and the combined organic phase was dried with Na_2SO_4, filtered then evaporated to afford crude epimeric alcohols (30 mg), which was dissolved in CH_2Cl_2 (2 mL). To the solution was added $NaHCO_3$ (55 mg, 0.65 mmol), Dess-Martin periodinane (110 mg, 0.26 mmol), then water (4.7 µL). The mixture was stirred for 1 h, diluted with hexane (8 mL), then filtered through a silica plug, washing with 1:4 ethyl acetate/hexane (10 mL). The eluant was evaporated to afford **S2-4** as an oil (22 mg, 9% in 4 steps). ^{1}H NMR (500 MHz, $CDCl_3$) δ 6.60 (dd, *J* = 17.3, 10.4 Hz, 1H), 6.44 (dd, *J* = 17.3, 2.0 Hz, 1H), 5.74 (dd, *J* = 10.4, 2.0 Hz, 1H), 2.95 – 2.88 (m, 2H), 2.21 (ddd, *J* = 14.2, 10.4, 5.1 Hz, 1H), 1.58 (ddd, *J* = 14.2, 10.4, 5.4 Hz, 1H), 1.39 (dddd, *J* = 11.8, 10.3, 5.1, 2.1 Hz, 2H), 1.26 (d, *J* = 12.0 Hz, 14H), 0.88 (t, *J* = 6.9 Hz, 3H). ^{13}C NMR (126 MHz, $CDCl_3$) δ 197.86, 130.52, 128.74, 62.38, 50.56, 31.90, 30.28, 29.67, 29.57, 29.51, 29.43, 29.31, 24.67, 22.68, 14.11.

GENERAL PROCEDURE FOR CYCLIC PEPTIDE SYNTHESES

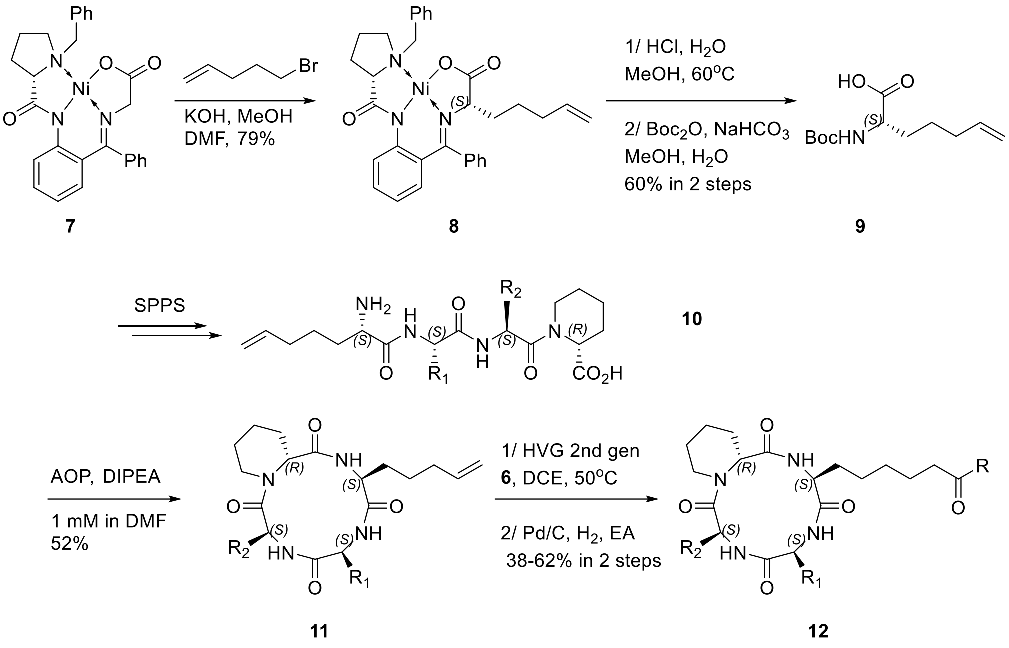

Scheme S3

Synthesis of 8: To a degased solution of complex **7** (8.41 g, 16.9 mmol) [2] and 5-bromo-1-pentene (2.1 mL, 17.8 mmol) in DMF (100 mL) was added dropwise KOH (8.9 mL, 2M in MeOH). The solution was stirred for 1 h, then quenched with AcOH (1 mL). The mixture was diluted with ethyl acetate (400 mL), then washed with water (5 x 100 mL). The organic phase was dried with Na_2SO_4, filtered, and then evaporated. Flash chromatography (0 – 15% acetone/CH_2Cl_2) afforded **8** as a red solid (7.6 g, 79%). ^{1}H NMR (500 MHz, $CDCl_3$) δ 8.16 (dd, *J* = 8.7, 1.2 Hz, 1H), 8.10 – 8.03 (m, 2H), 7.57 – 7.45 (m, 3H), 7.37 (t, *J* = 7.8 Hz, 2H), 7.25 – 7.14 (m, 2H), 6.94 (dt, *J* = 8.2, 1.4 Hz, 1H), 6.71 – 6.63 (m, 2H), 5.75 (ddt, *J* = 16.9, 10.2, 6.7 Hz, 1H), 5.05 – 4.94 (m, 2H), 4.47 (d, *J* = 12.7 Hz, 1H), 3.93 (dd, *J* = 8.1, 3.5 Hz, 1H), 3.68 – 3.46 (m, 4H), 2.79 (dt, *J* = 14.5, 7.2 Hz, 1H), 2.61 – 2.52 (m, 1H), 2.30 – 1.92 (m, 7H), 1.74 – 1.64 (m, 2H). ^{13}C NMR (126 MHz, $CDCl_3$) δ 180.39, 179.43, 170.40, 142.25, 137.78, 133.83, 133.26, 133.20, 132.16, 131.60, 129.73, 128.95, 128.94, 128.90, 127.63, 127.20, 126.54, 123.72, 120.76, 115.28, 70.37, 70.26, 63.10, 56.96, 34.84, 33.33, 30.76, 24.65, 23.65. HRMS (DART) calcd. for $C_{32}H_{34}N_3NiO_3^+$ $[M+H]^+$ 566.1948, found 566.1961.

Synthesis of 9: A solution of **8** (26.6 g, 47 mmol) in MeOH (200 mL) was added HCl (78 mL, 3 M in H_2O). The mixture was degassed, then refluxed for 1-2 h until the color turned green. The solution was evaporated, then CH_2Cl_2 (200 mL) and water (200 mL) were added. The phases were separated, then the organic phase was extracted with water (100 mL). The combined aqueous phase was neutralized with concentrated aqueous NH_4OH to pH = 8. The mixture was eluted through a column of DOWEX-50WX4 (40 mL), washing with ethanol (200 mL), water (200 mL), then eluted with 5% aqueous NH_4OH (500 mL). The ninhydrin-positive fractions were combined and evaporated to afforded the amino acid (4.2 g, 62%). To a solution of the amino acid (2.6 g, 18.2 mmol) in MeOH/H_2O (1:1, 100 mL) was added $NaHCO_3$ (2.73 g, 32.5 mmol) and Boc_2O (5.6 mL, 24.4 mmol). The solution was vigorously stirrred overnight, then quenched with HCl (40 mL, 1M in H_2O), and diluted with ethyl acetate (200 mL). The phases were separated, and the aqueous phase was extracted with EA (2 x 50 mL). The combined organic phase was dried with Na_2SO_4, filtered then evaporated. Flash chromatography (0 – 50% ethyl acetate/hexane) afforded **9** as a colourless oil (4.3 g, 97%). ^{1}H NMR (500 MHz, $CDCl_3$) δ 5.83 – 5.72 (m, 1H), 5.08 – 5.00 (m, 1H), 4.98 (d, *J* = 10.0 Hz, 2H), 4.23 (dd, *J* = 87.0, 7.0 Hz, 1H), 2.08 (p, *J* = 8.4 Hz, 2H), 1.88 (s, 1H), 1.67 (dt, *J* = 14.8, 7.5 Hz, 1H), 1.57 – 1.39 (m, 10H). ^{13}C NMR (126 MHz, $CDCl_3$) δ 177.28, 155.65, 137.90, 115.19, 80.28, 53.27, 33.17, 31.78, 31.16, 28.31, 24.55. HRMS (DART) calcd. for $C_{12}H_{22}NO_4^+$ $[M+H]^+$ 244.1543, found 244.1545.

General synthesis of 10: Linear tetrapeptides were prepared by standard solid phase peptide synthesis (SPPS) on 2-chlorotriyl (2-CTC resin). Briefly, 2-CTC resin (~ 1 mmol/g) was swollen in CH_2Cl_2 (10 mL/g), then Fmoc-(D)-Pip-OH (1 eq.) and DIPEA (1 eq.) was added, and the mixture was shaken for 1 h. The resin was filtered, then CH_2Cl_2/MeOH/DIPEA (10 mL/g, 17:2:1) was added, and the mixture was shaken for 15 min. The resin was then filtered and washed with DMF (3x, 10 mL/g). Deprotection was performed with 20% 4-Methylpiperidine/DMF (10 mL/g) for 5 min. The resin was then filtered and washed with DMF (3x, 10 mL/g). Coupling was performed with Fmoc-AA1-OH (2 eq.), PyAOP (2 eq.), DIPEA (6 eq.) in DMF (10 mL/g) for 5 min. The resin was then filtered and washed with DMF (3x, 10 mL/g). The coupling/deprotection/wash cycle as described was then performed for Fmoc-AA2-OH (2 eq.). The coupling as described was then performed for Boc-Uaa-OH (1 eq.), then the resin was filtered and washed with DMF (3x, 10 mL/g) followed by CH_2Cl_2 (3x, 10 mL/g). The protected linear tetrapeptide was eluted with hexafluoroisopropanol/CH_2Cl_2 (10 mL/g, 1:4), then the eluant was evaporated, redissolved in 4M HCl/dioxane (10 mL) and stirred for 30 min. The solution was then fully evaporated to give the crude linear tetrapeptide.

Synthesis of 11: To a solution of AOP (195 mg, 0.44 mmol) and DIPEA (383 µL, 2.2 mmol) in DMF (200 mL) was added using a syringe pump over 60 min, a solution of **10** (130 mg, 0.22 mmol) in DMF (22 mL). The solution was further stirred for 30 min, then fully evaporated. The residue was purified by flash chromatography (1 - 5% MeOH/CH_2Cl_2) to give the cyclic peptide **11** as a solid (61 mg, 52%). ^{1}H NMR (500 MHz, $CDCl_3$) δ 7.56 – 7.28 (m, 3H), 7.22 (q, *J* = 6.0 Hz, 5H), 7.10 (d, *J* = 7.0 Hz, 2H), 6.60 (d, *J* = 5.8 Hz, 1H), 6.49 (d, *J* = 10.1 Hz, 1H), 5.74 (ddt, *J* = 17.1, 10.5, 6.6 Hz, 1H), 5.35 (q, *J* = 8.3 Hz, 1H), 5.05 – 4.93 (m, 3H), 4.20 (q, *J* = 8.5 Hz, 1H), 3.94 (d, *J* = 13.3 Hz, 1H), 3.76 – 3.61 (m, 2H), 3.22 (ddd, *J* = 18.9, 13.7, 6.8 Hz, 2H), 3.12 – 2.94 (m, 3H), 2.09 – 1.95 (m, 4H), 1.84 – 1.69 (m, 2H), 1.60 – 1.48 (m, 3H), 1.37 – 1.26 (m, 3H). ^{13}C NMR (126 MHz, $CDCl_3$) δ 175.83, 173.62, 173.42, 171.42, 137.88, 136.99, 129.10, 128.89, 128.57, 128.56, 126.92, 126.72, 115.15, 62.81, 53.67, 50.93, 49.96, 43.94, 36.59, 35.21, 33.57, 33.25, 29.72, 28.76, 25.38, 25.12, 24.74, 23.98, 19.28. HRMS (DART) calcd. for $C_{31}H_{39}N_4O_4^+$ $[M+H]^+$ 531.2966, found 531.2968.

General synthesis of 12: To a solution of **11** (1 eq.) and **6a-c** (4 eq.) [or **S2-4** (4 eq.)] in dichloroethane (0.05 M), was added Hoveyda-Grubbs 2nd generation catalyst (20% w/w). The mixture was briefly flushed with nitrogen, then stirred at 50 °C overnight. Flash chromatography (0 – 60% ethyl acetylate/hexane) afforded the metathesized products, which were directly dissolved in ethyl acetate (0.05 M). Dry 5% Pd/C (20% w/w) was then added, and the solution was hydrogenated at 1 atm for 2 h. The mixture was filtered through a plug of silica gel, washing with ethyl acetate (20 mL/mol), then the combined organic phase was evaporated to afford **12**.

TD036: 10 mg, 32%. ^{1}H NMR (500 MHz, $CDCl_3$) δ 7.55 – 7.05 (m, 10H), 6.55 – 6.22 (m, 2H), 5.38 (dt, *J* = 9.9, 7.7 Hz, 1H), 5.04 (dd, *J* = 6.2, 2.6 Hz, 1H), 4.26 – 4.14 (m, 1H), 4.03 – 3.88 (m, 1H), 3.78 (ddt, *J* = 16.6, 11.5, 5.1 Hz, 1H), 3.71 – 3.61 (m, 1H), 3.38 – 2.93 (m, 4H), 2.87 (s, 1H), 2.66 – 2.47 (m, 1H), 2.38 (dddd, *J* = 16.7, 8.3, 6.0, 1.9 Hz, 1H), 2.32 – 1.89 (m, 4H), 1.83 – 0.99 (m, 30H), 0.90 (td, *J* = 7.0, 2.0 Hz, 3H). ^{13}C NMR (126 MHz, $CDCl_3$) δ 209.56, 175.70, 173.61, 173.34, 171.39, 136.98, 136.96, 129.10, 128.88, 128.57, 126.97, 126.71, 81.31, 62.91, 62.78, 53.60, 50.98, 50.94, 49.97, 43.97, 36.77, 36.60, 35.71, 35.66, 35.25, 31.91, 31.89, 30.51, 29.76, 29.71, 29.69, 29.67, 29.58, 29.55, 29.52, 29.43, 29.37, 29.32, 29.30, 29.14, 29.00, 28.70, 25.29, 25.10, 24.73, 23.99, 23.17, 23.08, 22.69, 22.27, 19.24, 14.12. HRMS (DART) calcd. for $C_{44}H_{63}N_4O_6^+$ $[M+H]^+$ 743.4742, found 743.4744.

TD034: 20 mg, 62%. ^{1}H NMR (500 MHz, $CDCl_3$) δ 7.44 (d, *J* = 10.3 Hz, 1H), 7.29 (t, *J* = 7.4 Hz, 2H), 7.23 (dd, *J* = 10.2, 7.2 Hz, 5H), 7.13 – 7.08 (m, 2H), 6.41 (d, *J* = 10.2 Hz, 1H), 6.29 (d, *J* = 6.0 Hz, 1H), 5.36 (q, *J* = 8.4 Hz, 1H), 5.01 (d, *J* = 5.8 Hz, 1H), 4.15 (q, *J* = 8.5 Hz, 1H), 3.95 (d, *J* = 13.3 Hz, 1H), 3.76 – 3.61 (m, 2H), 3.30 – 3.18 (m, 3H), 3.11 – 2.97 (m, 3H), 2.41 (dt, *J* = 17.3, 7.2 Hz, 1H), 2.24 (dt, *J* = 17.3, 7.4 Hz, 1H), 2.09 (d, *J* = 13.7 Hz, 1H), 2.02 – 1.93 (m, 1H), 1.84 – 1.04 (m, 33H), 0.88 (t, *J* = 6.8 Hz, 3H). ^{13}C NMR (126 MHz, $CDCl_3$) δ 207.77, 175.73, 173.62, 173.34, 171.41, 136.97, 136.95, 129.10, 128.88, 128.58, 128.57, 126.99, 126.72, 62.86, 59.70, 58.41, 53.51, 50.97, 49.99, 43.97, 36.80, 36.57, 35.22, 31.92, 31.87,

29.72, 29.63, 29.62, 29.55, 29.52, 29.47, 29.35, 29.30, 28.94, 28.65, 25.81, 25.22, 25.09, 23.99, 22.79, 22.70, 19.23, 14.14. HRMS (DART) calcd. for $C_{45}H_{65}N_4O_6^+$ $[M+H]^+$ 757.4899, found 757.4898.

TD034-R: 20 mg, 62%. ^{1}H NMR (500 MHz, $CDCl_3$) δ 7.47 (dd, *J* = 10.6, 6.4 Hz, 1H), 7.29 – 7.08 (m, 9H), 6.52 – 6.37 (m, 2H), 5.35 (dt, *J* = 10.2, 7.6 Hz, 1H), 5.02 (dd, *J* = 6.3, 2.3 Hz, 1H), 4.24 – 4.13 (m, 1H), 3.94 (dt, *J* = 13.4, 3.6 Hz, 1H), 3.82 – 3.70 (m, 1H), 3.70 – 3.57 (m, 1H), 3.31 – 3.19 (m, 2H), 3.03 (dddd, *J* = 24.2, 16.0, 13.5, 5.0 Hz, 2H), 2.48 – 2.33 (m, 1H), 2.31 – 2.18 (m, 1H), 2.08 (dt, *J* = 13.3, 4.2 Hz, 1H), 2.02 – 1.92 (m, 1H), 1.83 – 1.42 (m, 11H), 1.40 – 1.19 (m, 24H), 0.89 (d, *J* = 6.7 Hz, 3H). ^{13}C NMR (126 MHz, $CDCl_3$) δ 207.76, 175.67, 173.60, 173.34, 136.95, 129.10, 128.88, 128.58, 128.56, 126.97, 126.72, 62.69, 59.70, 58.42, 53.61, 50.97, 49.95, 43.98, 36.82, 36.76, 36.60, 35.25, 31.92, 31.87, 29.72, 29.63, 29.62, 29.52, 29.47, 29.37, 29.35, 29.30, 28.98, 28.65, 25.81, 25.23, 25.11, 23.99, 22.79, 22.70, 19.25, 14.14. HRMS (DART) calcd. for $C_{45}H_{65}N_4O_6^+$ $[M+H]^+$ 757.4899, found 757.4900.

TD037: 16 mg, 40%. ^{1}H NMR (500 MHz, $CDCl_3$) δ 7.48 (d, *J* = 10.2 Hz, 1H), 7.26 (ddt, *J* = 16.1, 12.2, 6.4 Hz, 7H), 7.10 (d, *J* = 7.2 Hz, 2H), 6.49 (dd, *J* = 34.5, 8.1 Hz, 2H), 5.35 (q, *J* = 8.5 Hz, 1H), 5.02 (d, *J* = 5.8 Hz, 1H), 4.17 (q, *J* = 8.5 Hz, 1H), 3.94 (d, *J* = 13.3 Hz, 1H), 3.74 (dt, *J* = 11.8, 6.2 Hz, 1H), 3.64 (t, *J* = 12.1 Hz, 1H), 3.30 – 3.19 (m, 2H), 3.10 – 2.90 (m, 3H), 2.39 (ddt, *J* = 22.6, 15.1, 7.1 Hz, 1H), 2.25 (dd, *J* = 16.3, 8.3 Hz, 1H), 2.06 (dd, *J* = 16.1, 11.3 Hz, 1H), 1.96 (d, *J* = 13.6 Hz, 1H), 1.86 – 1.73 (m, 2H), 1.73 – 1.36 (m, 10H), 1.36 – 1.10 (m, 14H), 0.88 (t, *J* = 6.6 Hz, 3H). ^{13}C NMR (126 MHz, $CDCl_3$) δ 207.79, 175.76, 173.62, 173.38, 171.45, 136.96, 129.10, 128.89, 128.58, 128.56, 126.97, 126.73, 62.72, 59.69, 58.43, 53.60, 50.93, 49.95, 43.96, 36.84, 36.59, 35.23, 31.87, 31.71, 29.45, 29.30, 29.24, 29.13, 29.01, 28.66, 25.80, 25.23, 25.10, 23.98, 22.79, 22.69, 22.63, 19.26, 14.10. HRMS (DART) calcd. for $C_{41}H_{57}N_4O_6^+$ $[M+H]^+$ 701.4273, found 701.4270.

TD038: 18 mg, 38%. ^{1}H NMR (500 MHz, $CDCl_3$) δ 7.48 (d, *J* = 10.3 Hz, 1H), 7.29 – 7.17 (m, 7H), 7.10 (d, *J* = 7.1 Hz, 2H), 6.48 (dd, *J* = 33.9, 8.1 Hz, 2H), 5.35 (q, *J* = 8.4 Hz, 1H), 5.02 (d, *J* = 5.9 Hz, 1H), 4.17 (q, *J* = 8.5 Hz, 1H), 3.94 (d, *J* = 13.3 Hz, 1H), 3.75 (dt, *J* = 11.9, 6.0 Hz, 1H), 3.63 (t, *J* = 12.1 Hz, 1H), 3.31 – 3.19 (m, 2H), 3.11 – 2.93 (m, 3H), 2.39 (s, 1H), 2.24 (dt, *J* = 17.6, 7.3 Hz, 1H), 2.07 (d, *J* = 13.3 Hz, 1H), 1.97 (d, *J* = 13.8 Hz, 1H), 1.79 – 1.19 (m, 41H), 0.88 (td, *J* = 6.9, 2.2 Hz, 4H). ^{13}C NMR (126 MHz, $CDCl_3$) δ 207.79, 175.71, 173.61, 173.37, 171.45, 136.96, 129.10, 128.89, 128.58, 128.56, 126.97, 126.73, 62.66, 59.69, 58.43, 53.61, 50.94, 49.94, 43.97, 36.83, 36.60, 35.25, 31.94, 31.88, 29.71, 29.70, 29.67, 29.64, 29.53, 29.48, 29.38, 29.31, 29.01, 28.65, 25.81, 25.24, 25.11, 23.98, 22.79, 22.71, 19.26, 14.15. HRMS (DART) calcd. for $C_{49}H_{73}N_4O_6^+$ $[M+H]^+$ 813.5525, found 813.5519.

REFERENCES

(1) Son, S. I.; Cao, J.; Zhu, C.-L.; Miller, S. P.; Lin, H. Activity-Guided Design of HDAC11-Specific Inhibitors. *ACS Chemical Biology* **2019**. https://doi.org/10.1021/acschembio.9b00292.

(2) Gu, X.; Ndungu, J. M.; Qiu, W.; Ying, J.; Carducci, M. D.; Wooden, H.; Hruby, V. J. Large Scale Enantiomeric Synthesis, Purification, and Characterization of ω-Unsaturated Amino Acids via a Gly-Ni(II)-BPB-Complex. *Tetrahedron* **2004**, *60* (37), 8233–8243. https://doi.org/10.1016/j.tet.2004.06.087.

SHIMADZU LabSolutions

Analysis Report

TD036

<Sample Information>

Sample Name : TD036_purity
Sample ID :
Data Filename : TD036_purity_1.lcd
Method Filename : 0-100%_15min_purity.lcm
Batch Filename : postrun_20221220.lcb
Vial # : 1-B6
Sample Type : Unknown
Injection Volume : 20 uL
Date Acquired : 12/6/2022 1:07:28 PM
Acquired by : System Administrator
Date Processed : 12/20/2022 8:59:25 PM
Processed by : System Administrator

<Chromatogram>

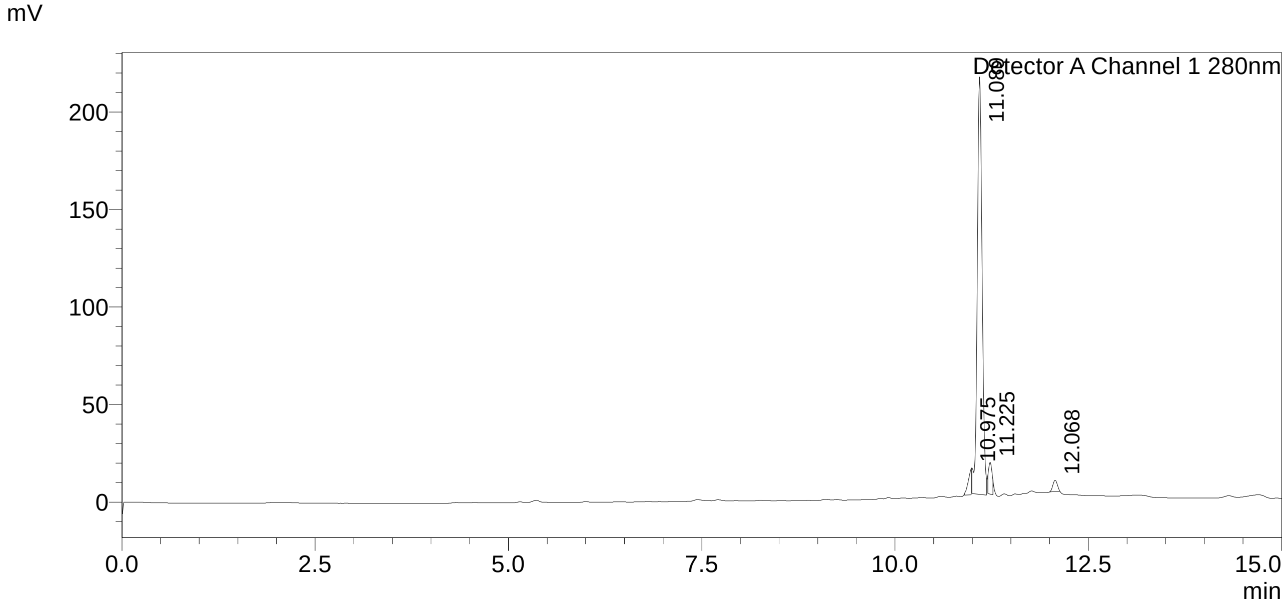

<Peak Table>

Detector A Channel 1 280nm

Peak#	Ret. Time	Area	Area%
1	10.975	36385	3.469
2	11.089	937335	89.375
3	11.225	51404	4.901
4	12.068	23642	2.254
Total		1048765	100.000

C:\LabSolutions\Data\Tu\Manuscript1_purity\TD036_purity_1.lcd

SHIMADZU LabSolutions

Analysis Report

TD034

<Sample Information>

Sample Name	: TD034_purity_1		
Sample ID	:		
Data Filename	: TD034_purity_1.lcd		
Method Filename	: 0-100%_15min_purity.lcm		
Batch Filename	: postrun_20221220.lcb		
Vial #	: 1-B7	Sample Type	: Unknown
Injection Volume	: 20 uL		
Date Acquired	: 12/6/2022 1:30:48 PM	Acquired by	: System Administrator
Date Processed	: 12/20/2022 9:02:49 PM	Processed by	: System Administrator

<Chromatogram>

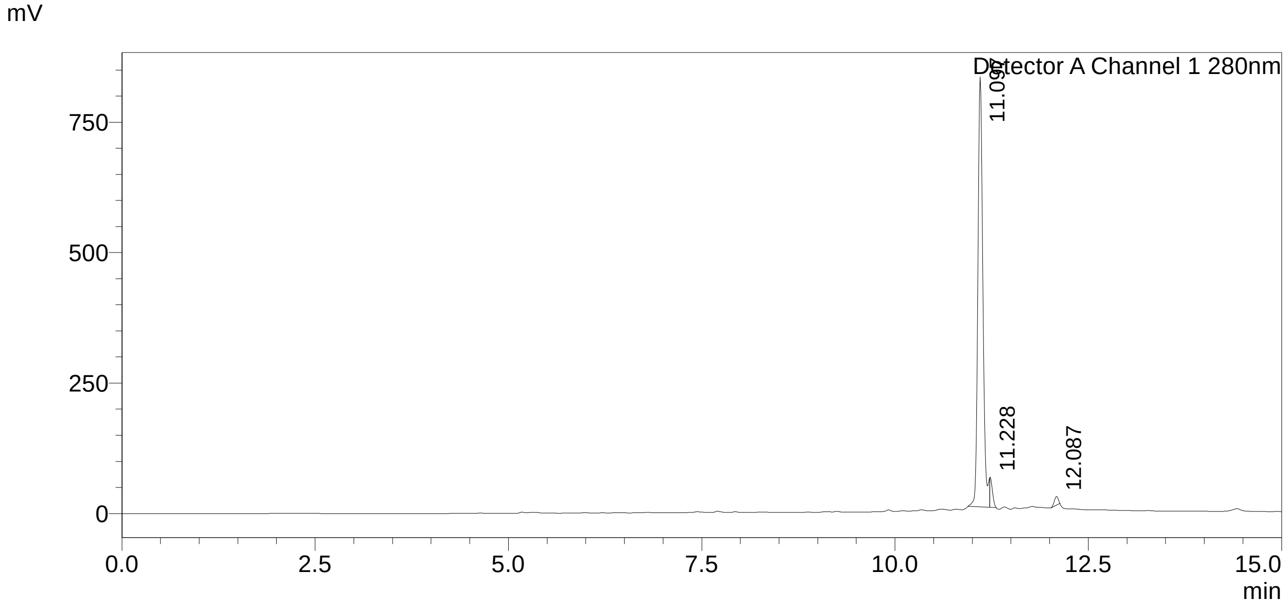

<Peak Table>

Detector A Channel 1 280nm

Peak#	Ret. Time	Area	Area%
1	11.097	3755774	95.357
2	11.228	123016	3.123
3	12.087	59876	1.520
Total		3938665	100.000

C:\LabSolutions\Data\Tu\Manuscript1_purity\TD034_purity_1.lcd

SHIMADZU LabSolutions

Analysis Report

TD034-R

<Sample Information>

Sample Name : TD034(R)_purity_1
Sample ID :
Data Filename : TD034(R)_purity_1.lcd
Method Filename : 0-100%_15min_purity.lcm
Batch Filename : postrun_20221220.lcb
Vial # : 1-C1
Sample Type : Unknown
Injection Volume : 50 uL
Date Acquired : 12/9/2022 3:19:24 PM
Acquired by : System Administrator
Date Processed : 12/20/2022 9:08:40 PM
Processed by : System Administrator

<Chromatogram>

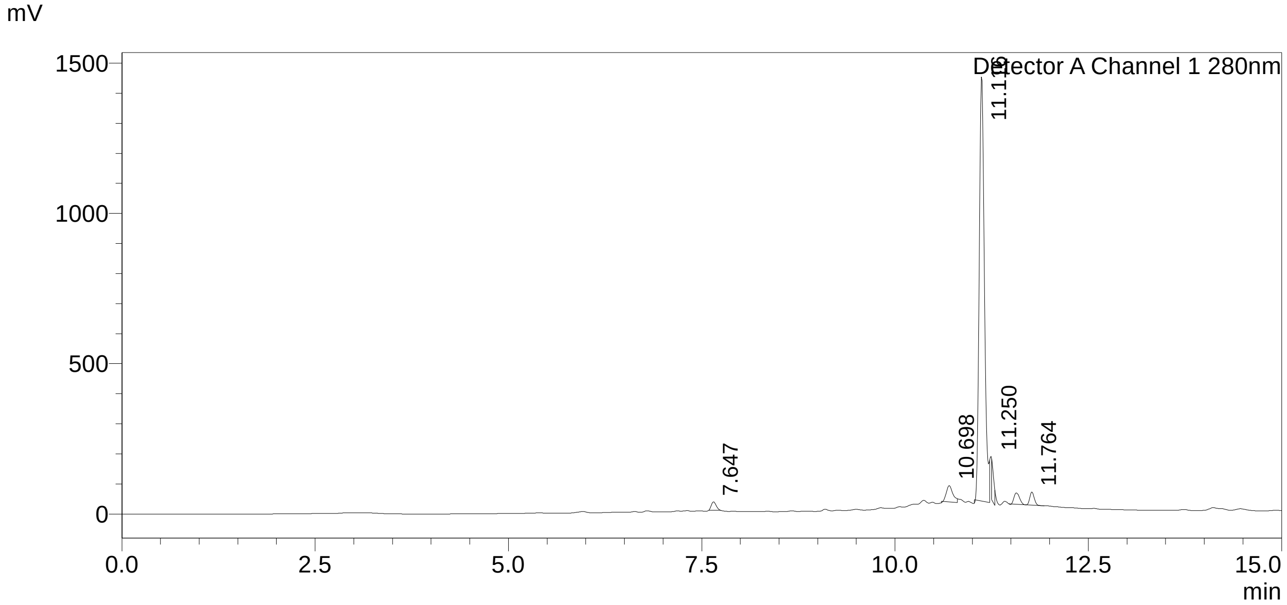

<Peak Table>

Detector A Channel 1 280nm

Peak#	Ret. Time	Area	Area%
1	7.647	121558	1.586
2	10.698	324012	4.227
3	11.116	6652898	86.803
4	11.250	209573	2.734
5	11.764	356353	4.649
Total		7664394	100.000

C:\LabSolutions\Data\Tu\Manuscript1_purity\TD034(R)_purity_1.lcd

SHIMADZU LabSolutions

Analysis Report

TD037

<Sample Information>

Sample Name : TD037_purity_1
Sample ID :
Data Filename : TD037_purity_1.lcd
Method Filename : 0-100%_15min_purity.lcm
Batch Filename : postrun_20221220.lcb
Vial # : 1-C2
Sample Type : Unknown
Injection Volume : 50 uL
Date Acquired : 12/9/2022 4:14:14 PM
Acquired by : System Administrator
Date Processed : 12/20/2022 9:07:15 PM
Processed by : System Administrator

<Chromatogram>

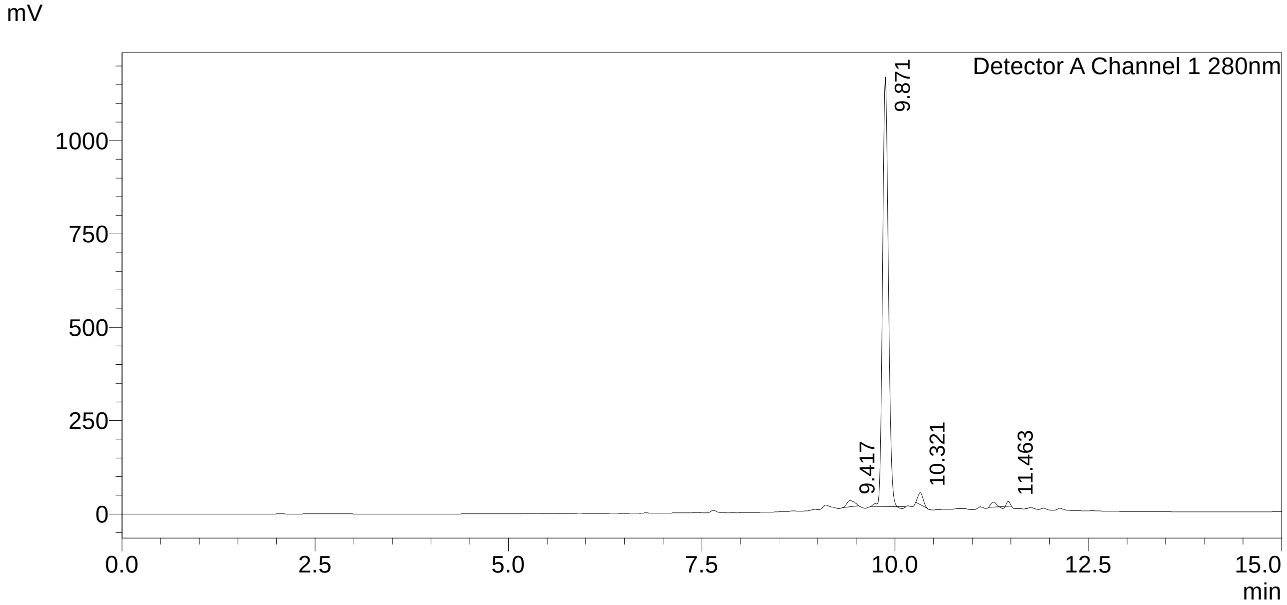

<Peak Table>

Detector A Channel 1 280nm

Peak#	Ret. Time	Area	Area%
1	9.417	122079	1.918
2	9.871	6007605	94.399
3	10.321	146245	2.298
4	11.463	88098	1.384
Total		6364027	100.000

C:\LabSolutions\Data\Tu\Manuscript1_purity\TD037_purity_1.lcd

SHIMADZU LabSolutions

Analysis Report

TD038

<Sample Information>

Sample Name	: TD038_purity_1		
Sample ID	:		
Data Filename	: TD038_purity_1.lcd		
Method Filename	: 0-100%_15min_purity.lcm		
Batch Filename	: postrun_20221220.lcb		
Vial #	: 1-C3	Sample Type	: Unknown
Injection Volume	: 50 uL		
Date Acquired	: 12/9/2022 4:31:07 PM	Acquired by	: System Administrator
Date Processed	: 12/20/2022 9:07:46 PM	Processed by	: System Administrator

<Chromatogram>

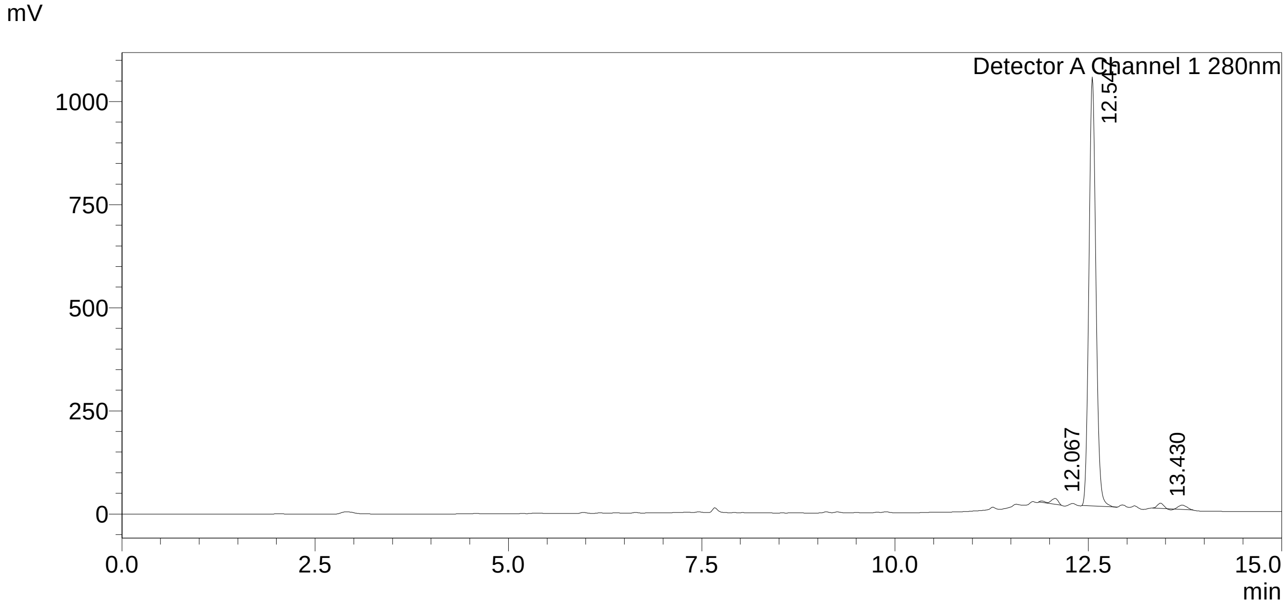

<Peak Table>

Detector A Channel 1 280nm

Peak#	Ret. Time	Area	Area%
1	12.067	98802	1.453
2	12.547	6559751	96.457
3	13.430	142177	2.091
Total		6800730	100.000

C:\LabSolutions\Data\Tu\Manuscript1_purity\TD038_purity_1.lcd

SHIMADZU LabSolutions

Analysis Report

Trapoxin A

<Sample Information>

Sample Name : TrapoxinA_purity_1
Sample ID :
Data Filename : TrapoxinA_purity_1.lcd
Method Filename : 0-100%_15min_purity.lcm
Batch Filename : postrun_20221220.lcb
Vial # : 1-A9
Sample Type : Unknown
Injection Volume : 40 uL
Date Acquired : 12/20/2022 5:23:20 PM
Acquired by : System Administrator
Date Processed : 12/20/2022 9:27:39 PM
Processed by : System Administrator

<Chromatogram>

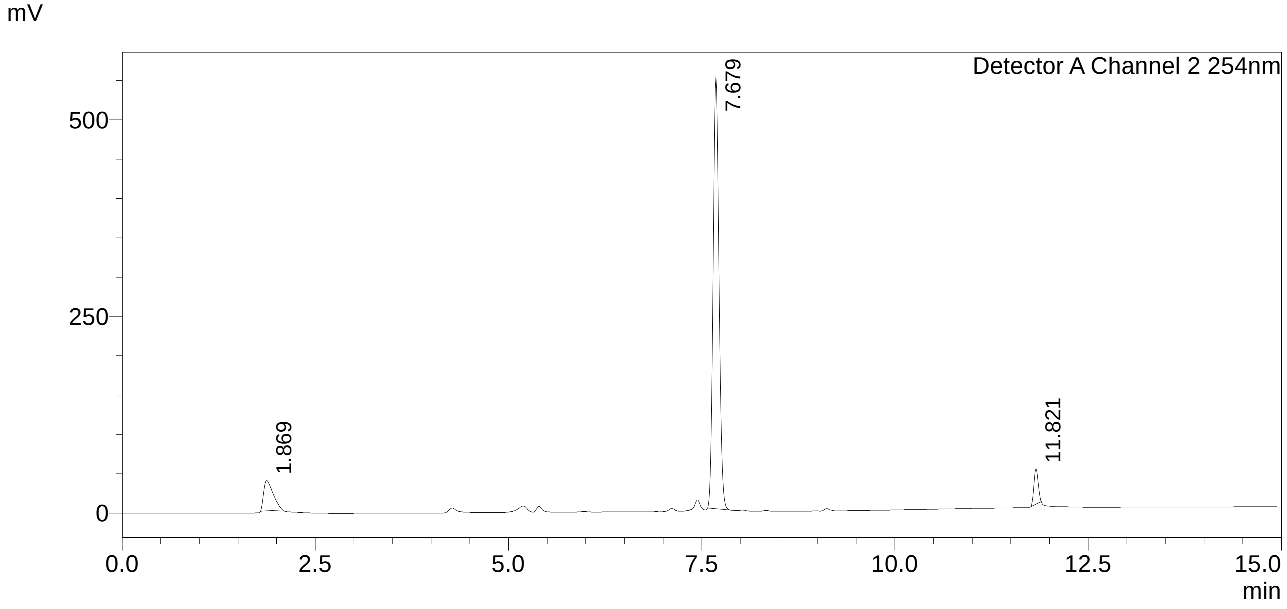

<Peak Table>

Detector A Channel 2 254nm

Peak#	Ret. Time	Area	Area%
1	1.869	328791	9.565
2	7.679	2935530	85.402
3	11.821	172980	5.032
Total		3437301	100.000

C:\LabSolutions\Data\Tu\Manuscript1_purity\TrapoxinA_purity_1.lcd

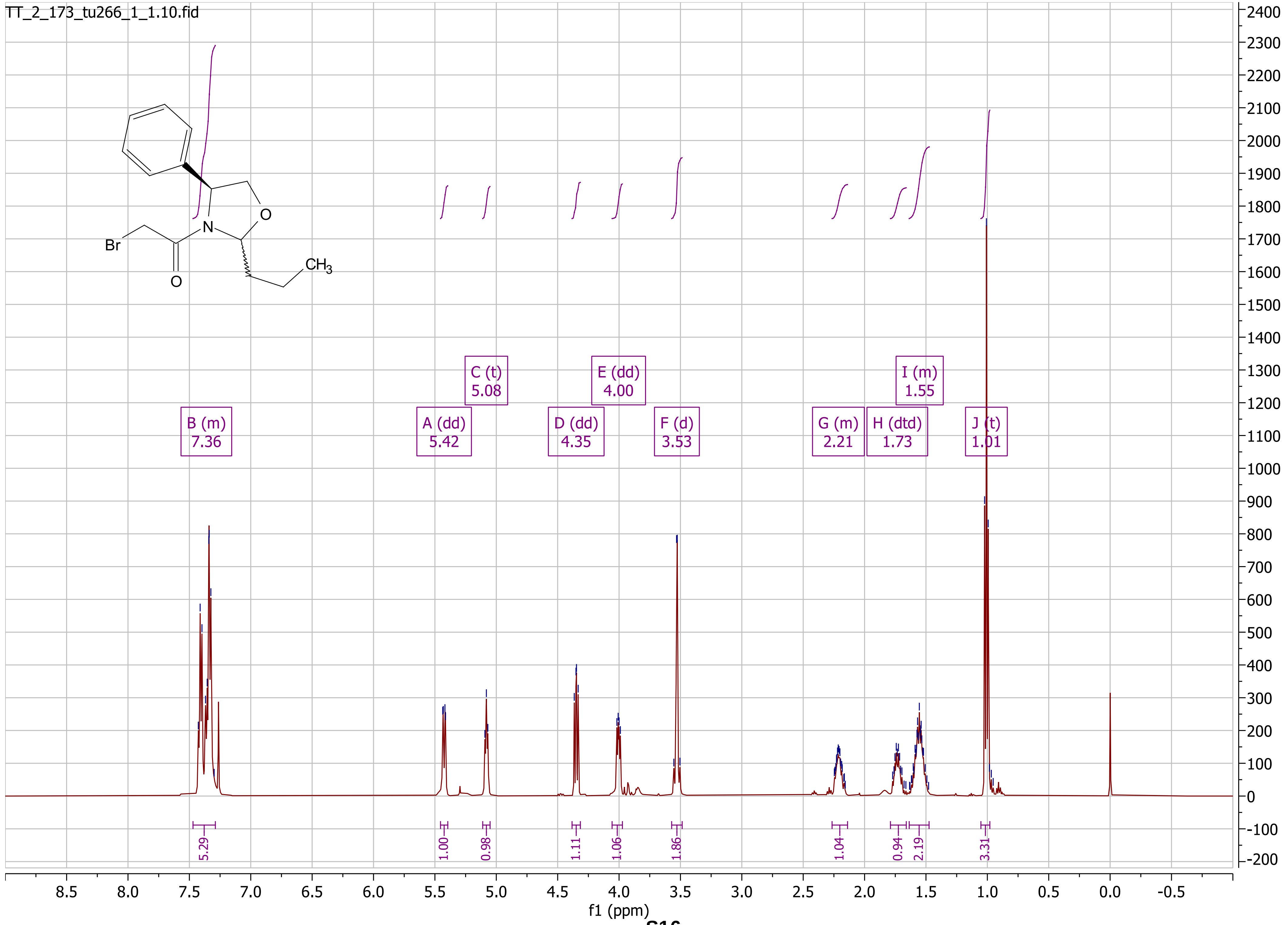

TT_2_173_tu266_1_1.10.fid
Br
N
O
O
CH3
B (m)
7.36
A (dd)
5.42
C (t)
5.08
D (dd)
4.35
E (dd)
4.00
F (d)
3.53
G (m)
2.21
H (dtd)
1.73
I (m)
1.55
J (t)
1.01
5.29
1.00
0.98
1.11
1.06
1.86
1.04
0.94
2.19
3.31
f1 (ppm)

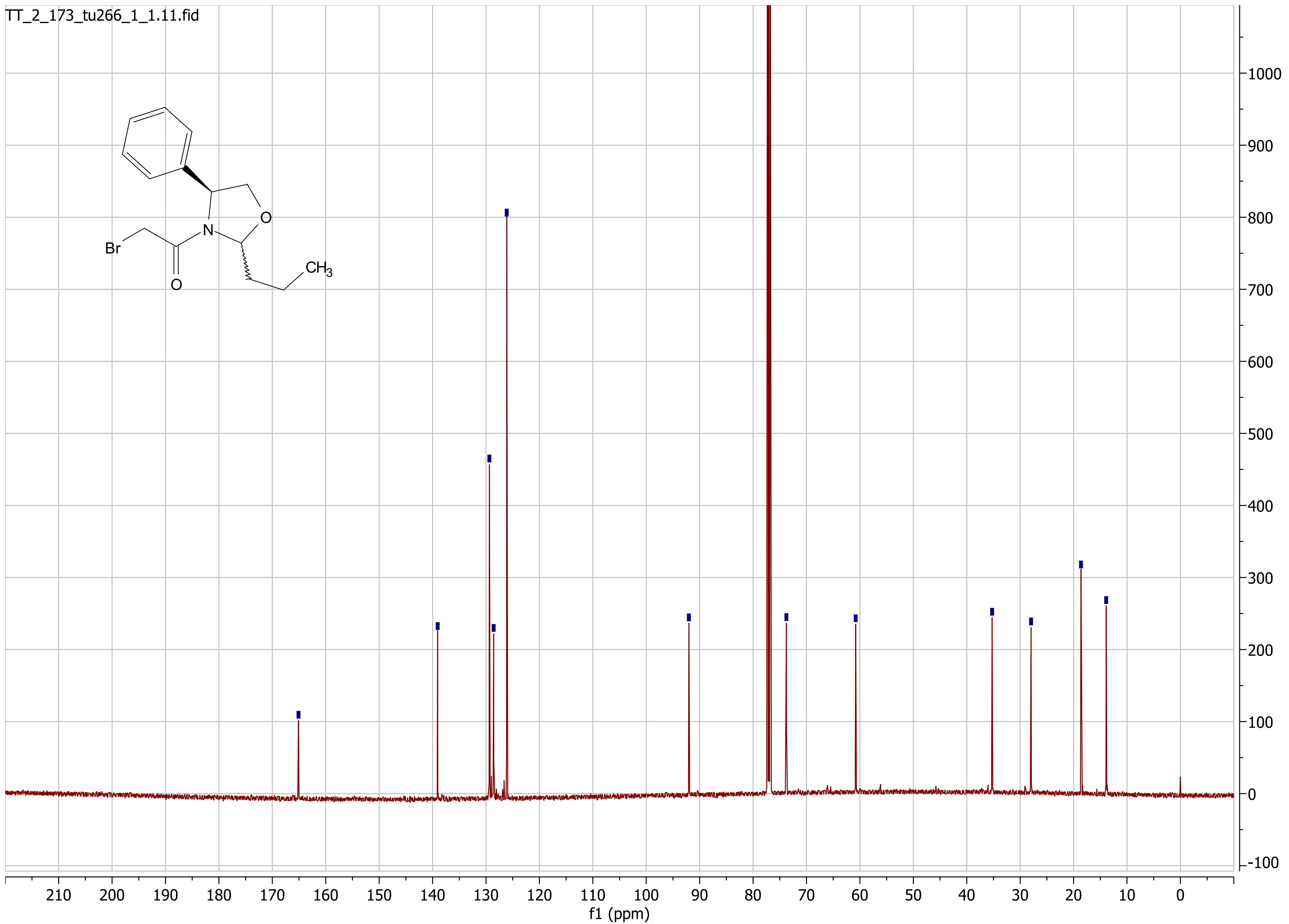
TT_2_173_tu266_1_1.11.fid
Br
N
O
O
CH3
f1 (ppm)

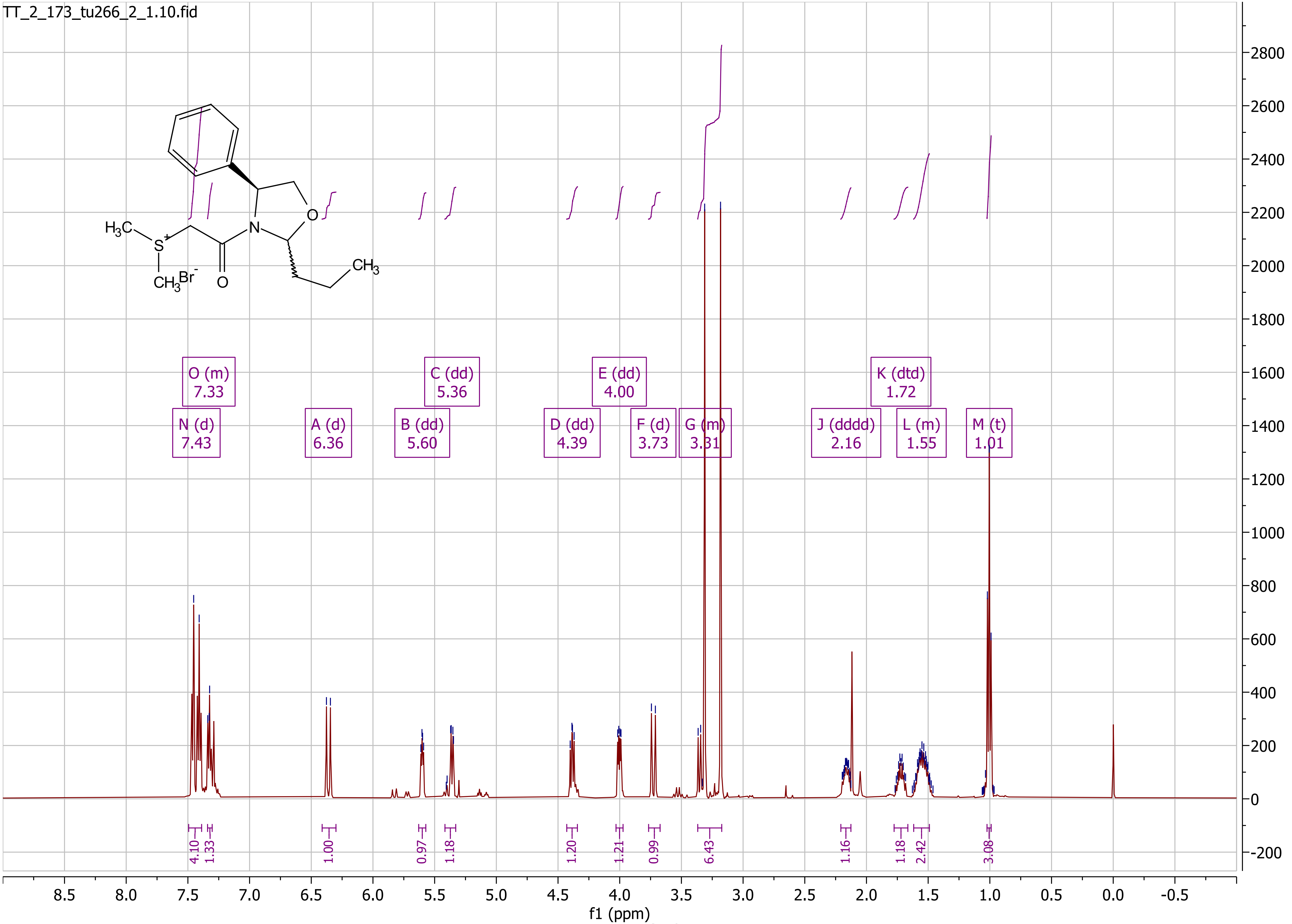
TT_2_173_tu266_2_1.10.fid
O (m) 7.33
N (d) 7.43
A (d) 6.36
B (dd) 5.60
C (dd) 5.36
D (dd) 4.39
E (dd) 4.00
F (d) 3.73
G (m) 3.31
J (dddd) 2.16
K (dtd) 1.72
L (m) 1.55
M (t) 1.01
4.10
1.33
1.00
0.97
1.18
1.20
1.21
0.99
6.43
1.16
1.18
2.42
3.08
f1 (ppm)

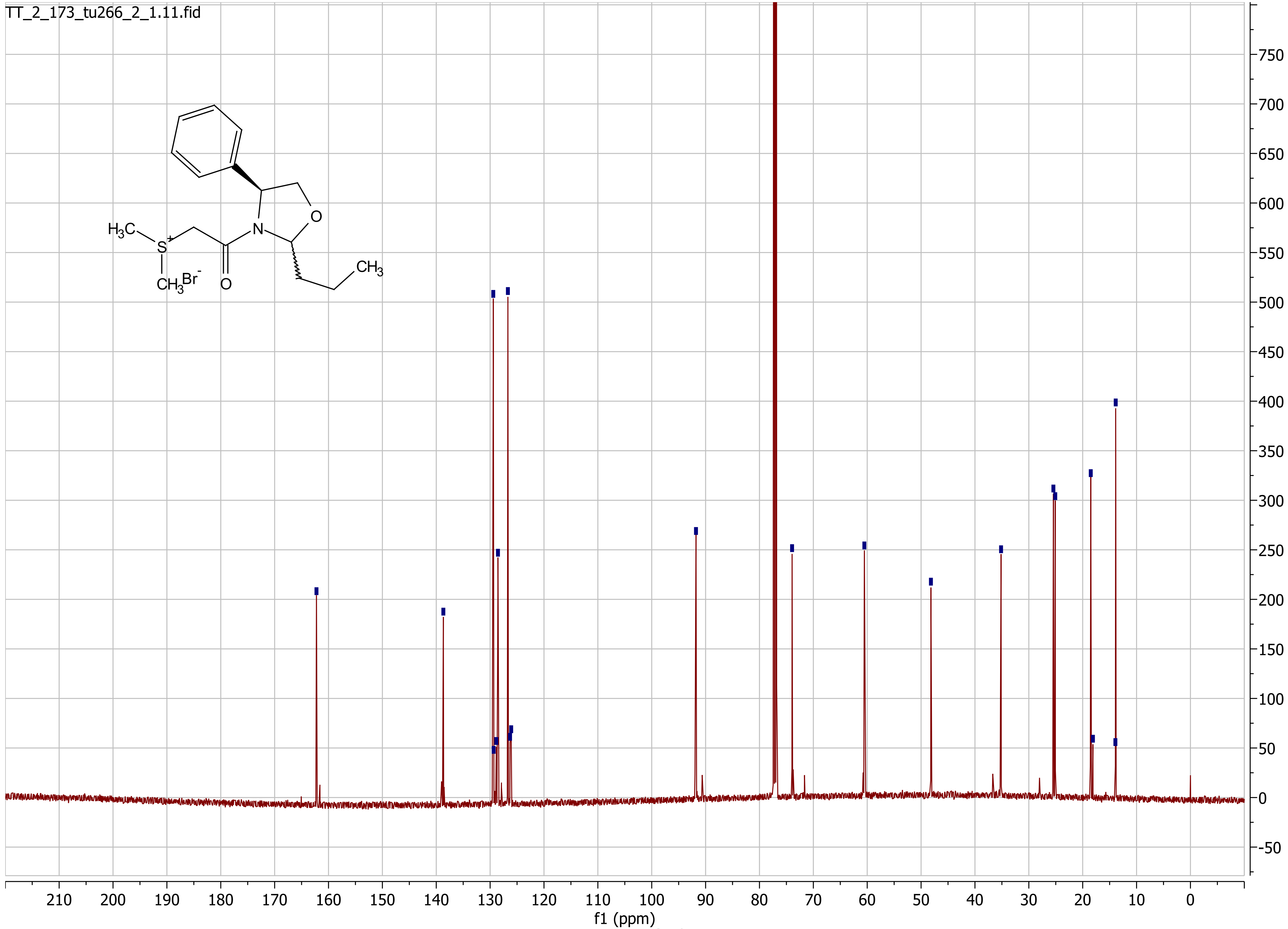
TT_2_173_tu266_2_1.11.fid
H3C
S+
CH3
Br-
O
N
O
CH3
f1 (ppm)

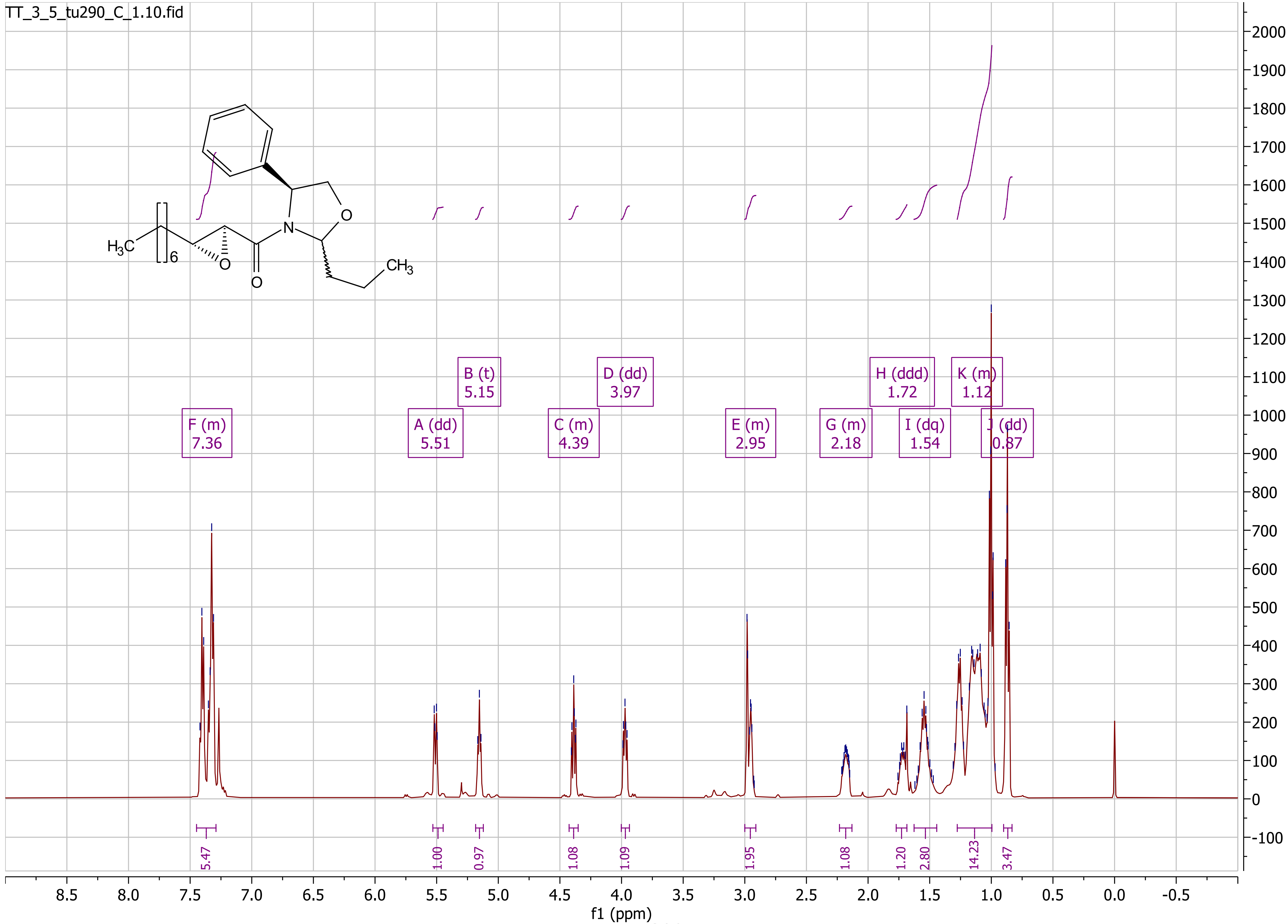
TT_3_5_tu290_C_1.10.fid
H_3C
6
O
O
N
O
CH_3
F (m) 7.36
A (dd) 5.51
B (t) 5.15
C (m) 4.39
D (dd) 3.97
E (m) 2.95
G (m) 2.18
H (ddd) 1.72
I (dq) 1.54
K (m) 1.12
J (dd) 0.87
5.47
1.00
0.97
1.08
1.09
1.95
1.08
1.20
2.80
14.23
3.47
f1 (ppm)

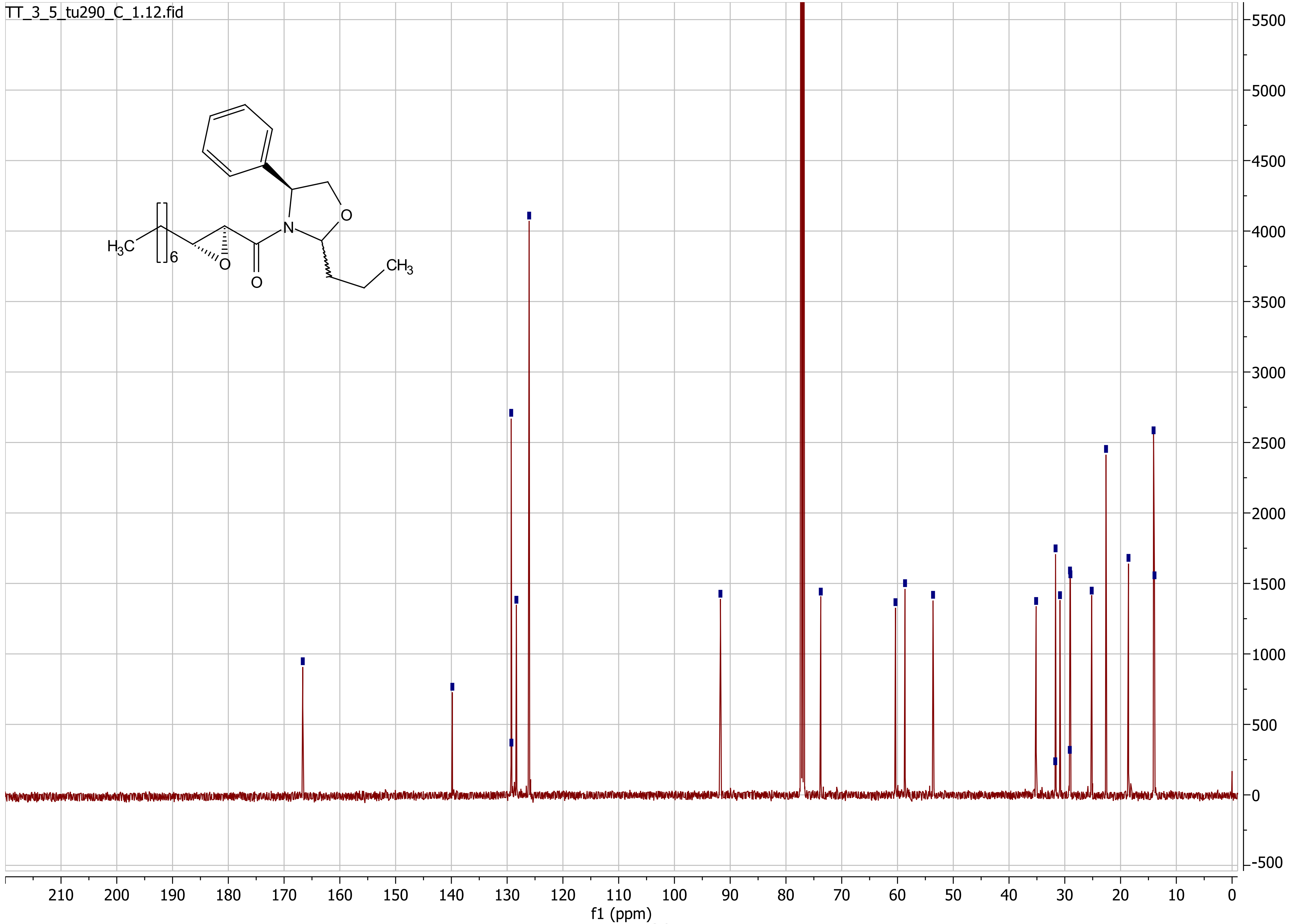
TT_3_5_tu290_C_1.12.fid
H3C
6
O
O
N
O
CH3
5500
5000
4500
4000
3500
3000
2500
2000
1500
1000
500
0
-500
210 200 190 180 170 160 150 140 130 120 110 100 90 80 70 60 50 40 30 20 10 0
f1 (ppm)

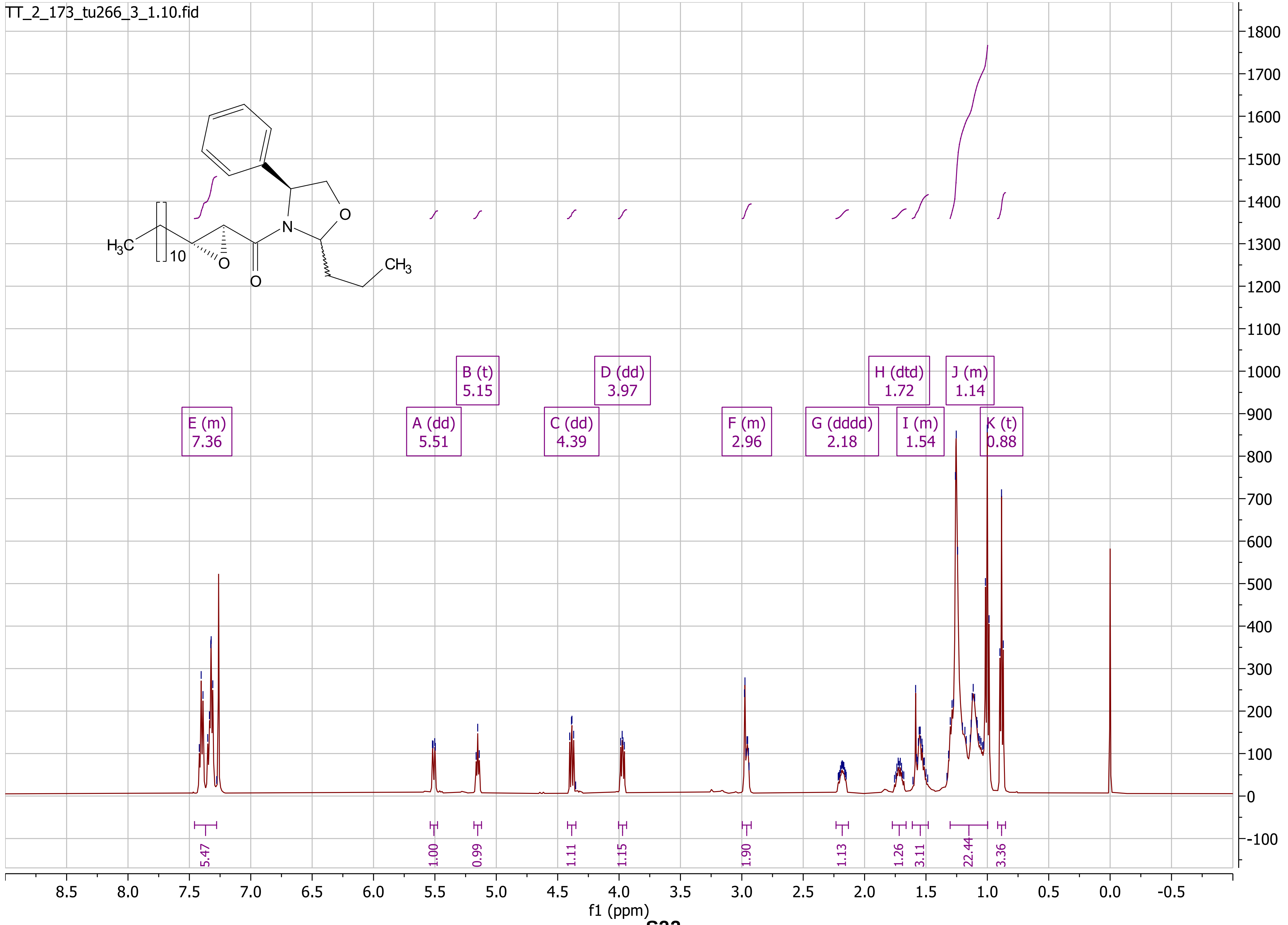
TT_2_173_tu266_3_1.10.fid
H3C
10
O
O
N
O
CH3
E (m)
7.36
A (dd)
5.51
B (t)
5.15
C (dd)
4.39
D (dd)
3.97
F (m)
2.96
G (dddd)
2.18
H (dtd)
1.72
I (m)
1.54
J (m)
1.14
K (t)
0.88
5.47
1.00
0.99
1.11
1.15
1.90
1.13
1.26
3.11
22.44
3.36
f1 (ppm)

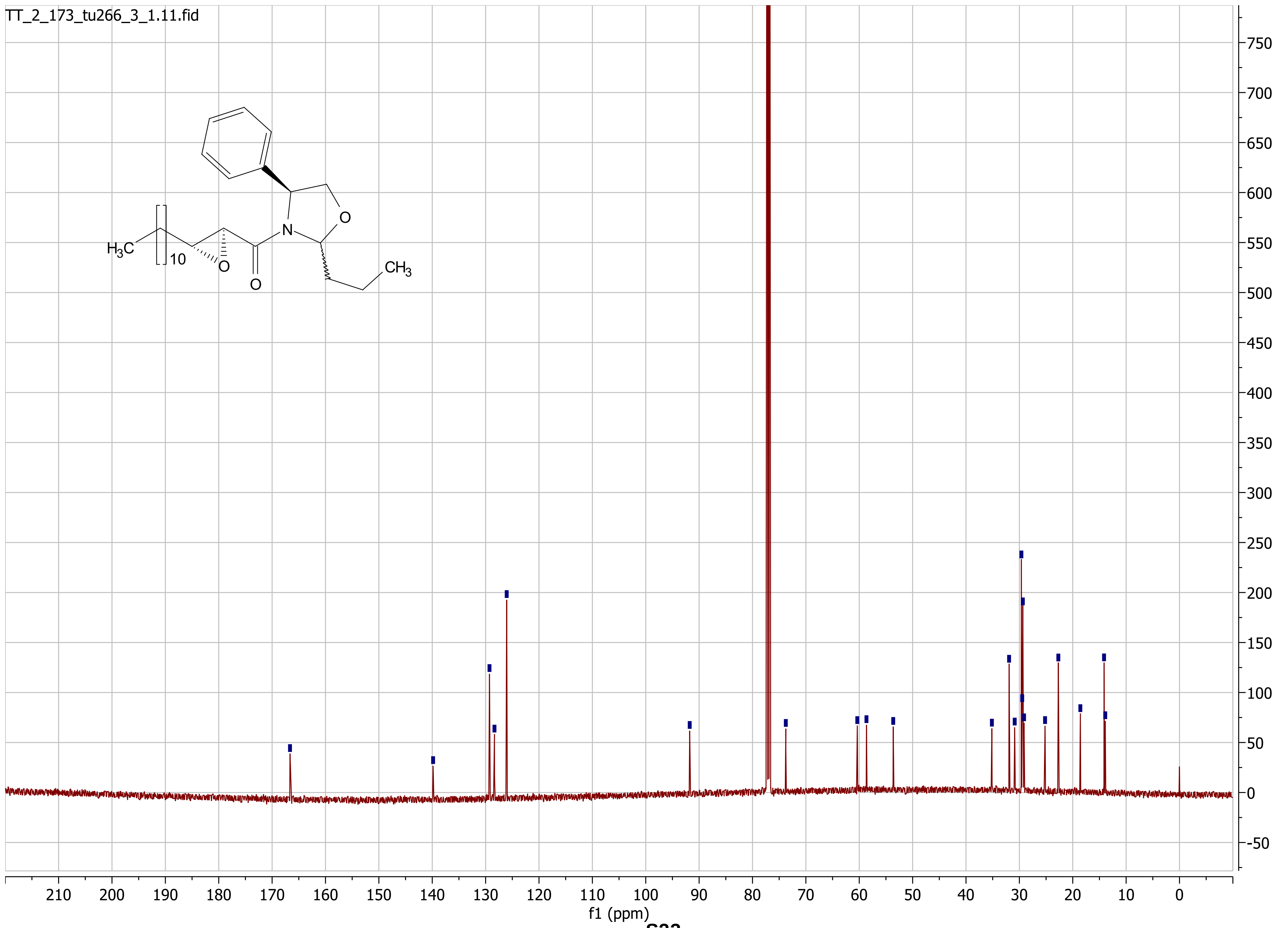
TT_2_173_tu266_3_1.11.fid
H3C
10
O
O
N
O
CH3
750
700
650
600
550
500
450
400
350
300
250
200
150
100
50
0
-50
210 200 190 180 170 160 150 140 130 120 110 100 90 80 70 60 50 40 30 20 10 0
f1 (ppm)

TT_3_5_tu290_C_2.10.fid

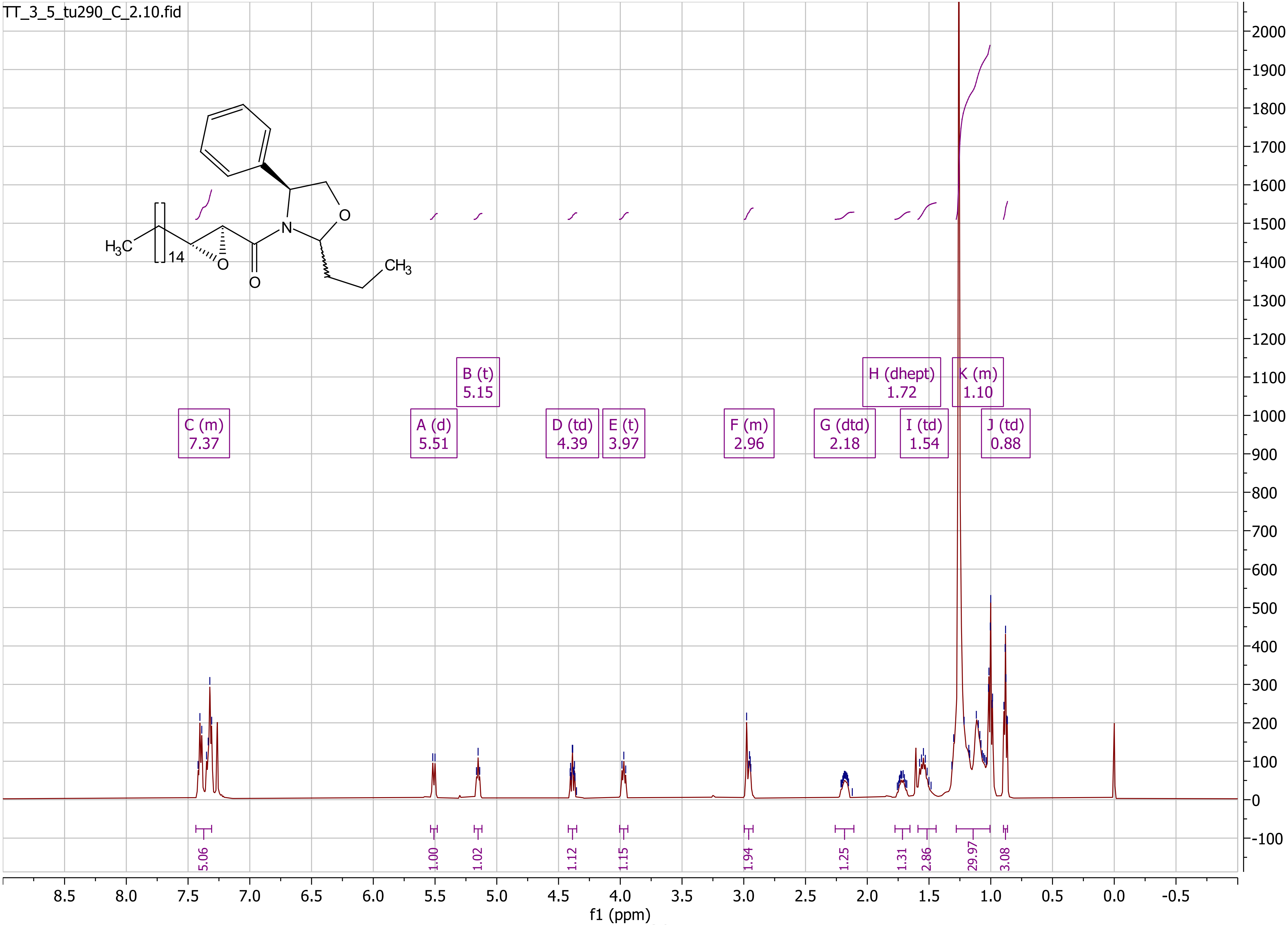

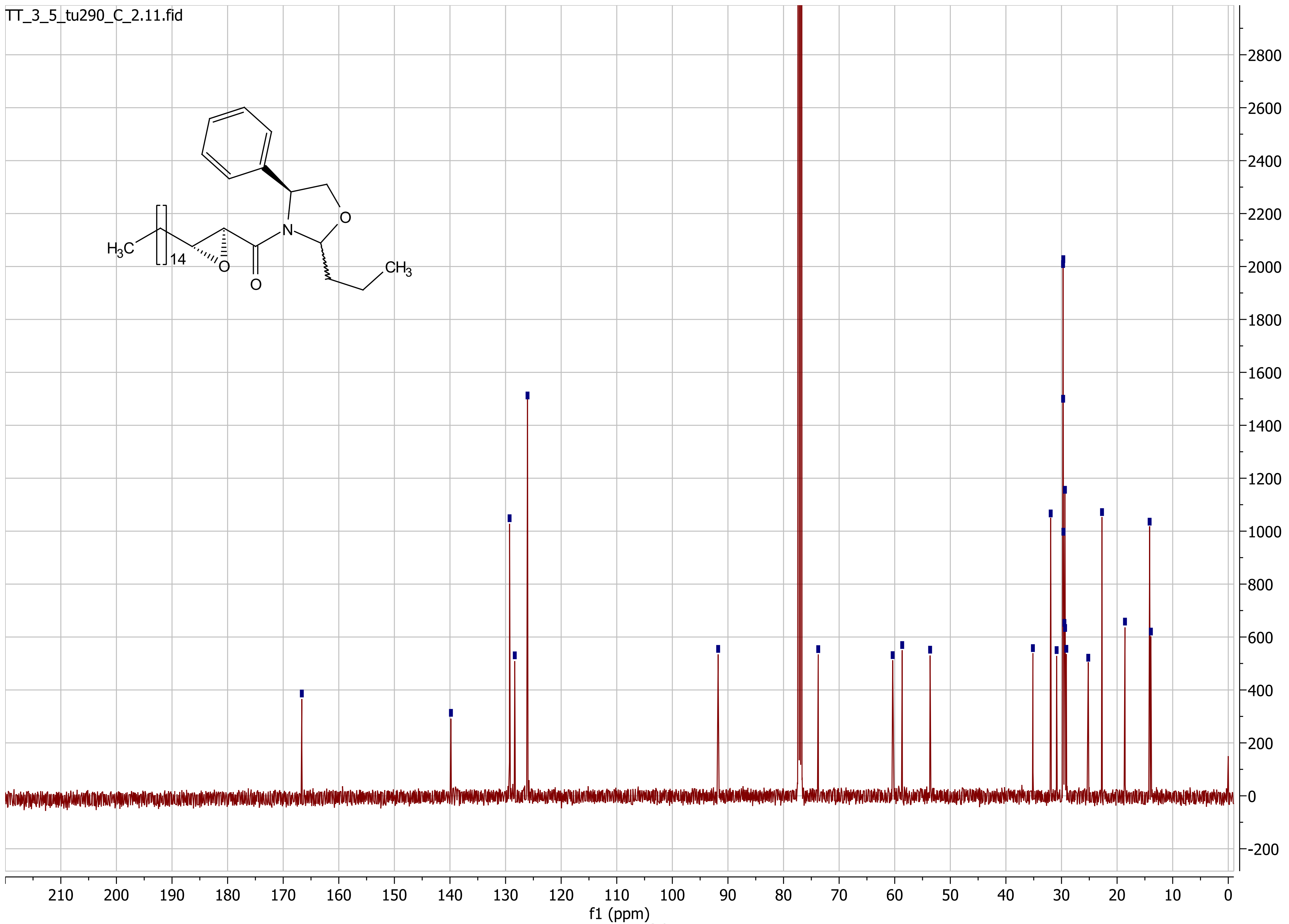
TT_3_5_tu290_C_2.11.fid
H3C
14
O
O
N
O
CH3
f1 (ppm)
210 200 190 180 170 160 150 140 130 120 110 100 90 80 70 60 50 40 30 20 10 0
2800 2600 2400 2200 2000 1800 1600 1400 1200 1000 800 600 400 200 0 -200

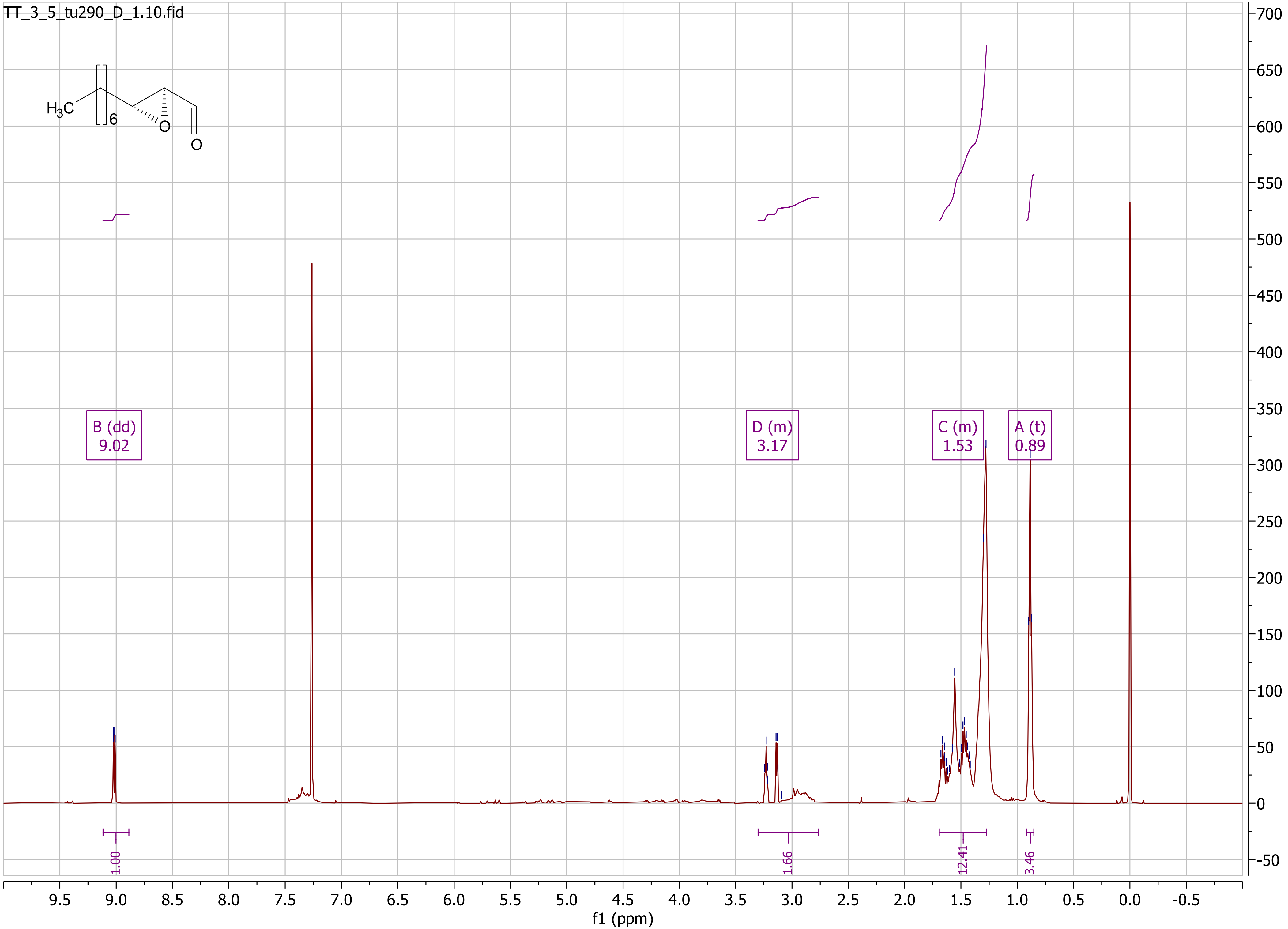

TT_3_5_tu290_D_1.10.fid
H3C
6
O
O
B (dd)
9.02
D (m)
3.17
C (m)
1.53
A (t)
0.89
1.00
1.66
12.41
3.46
f1 (ppm)

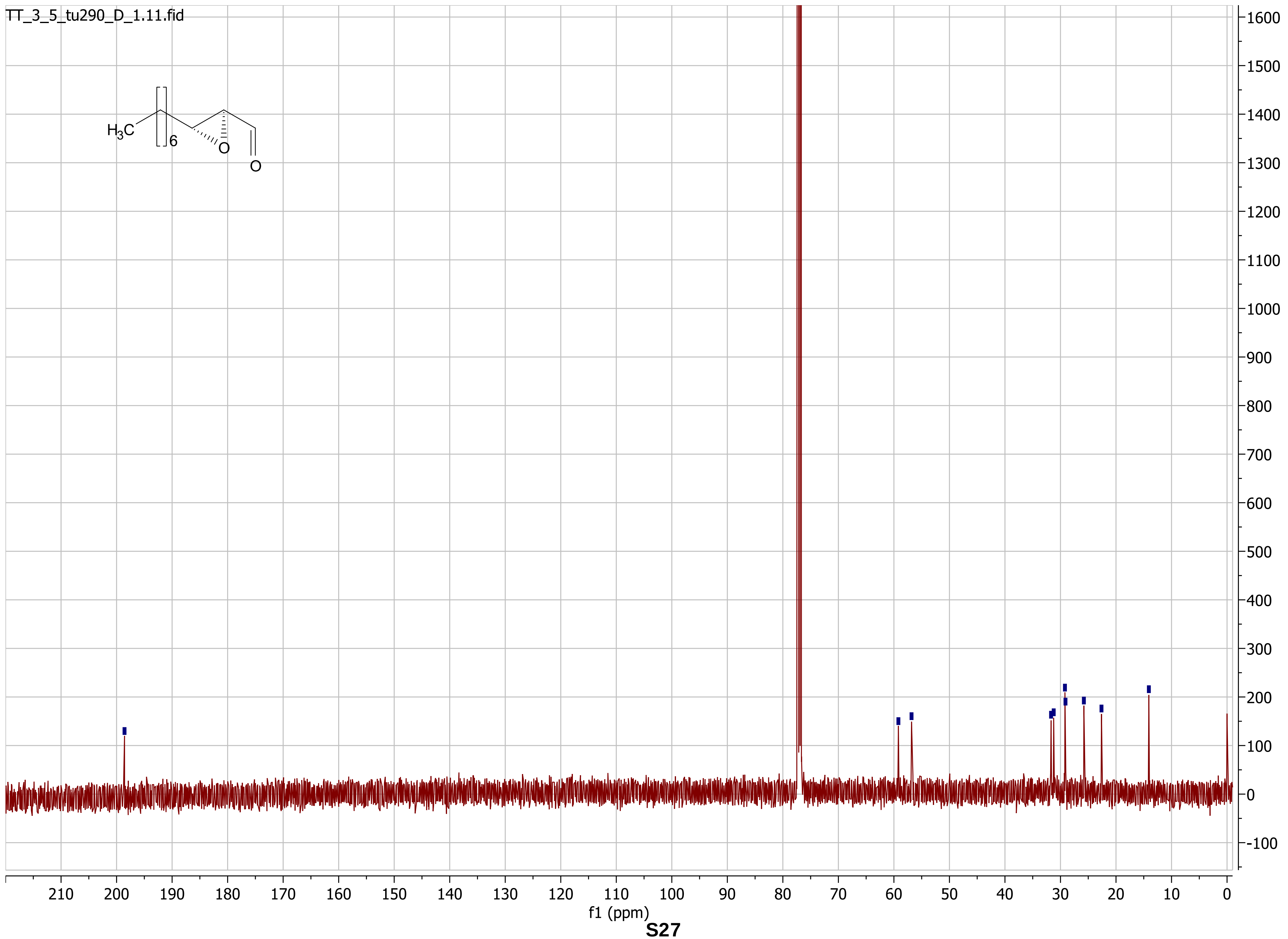
TT_3_5_tu290_D_1.11.fid
H3C
6
O
O
f1 (ppm)

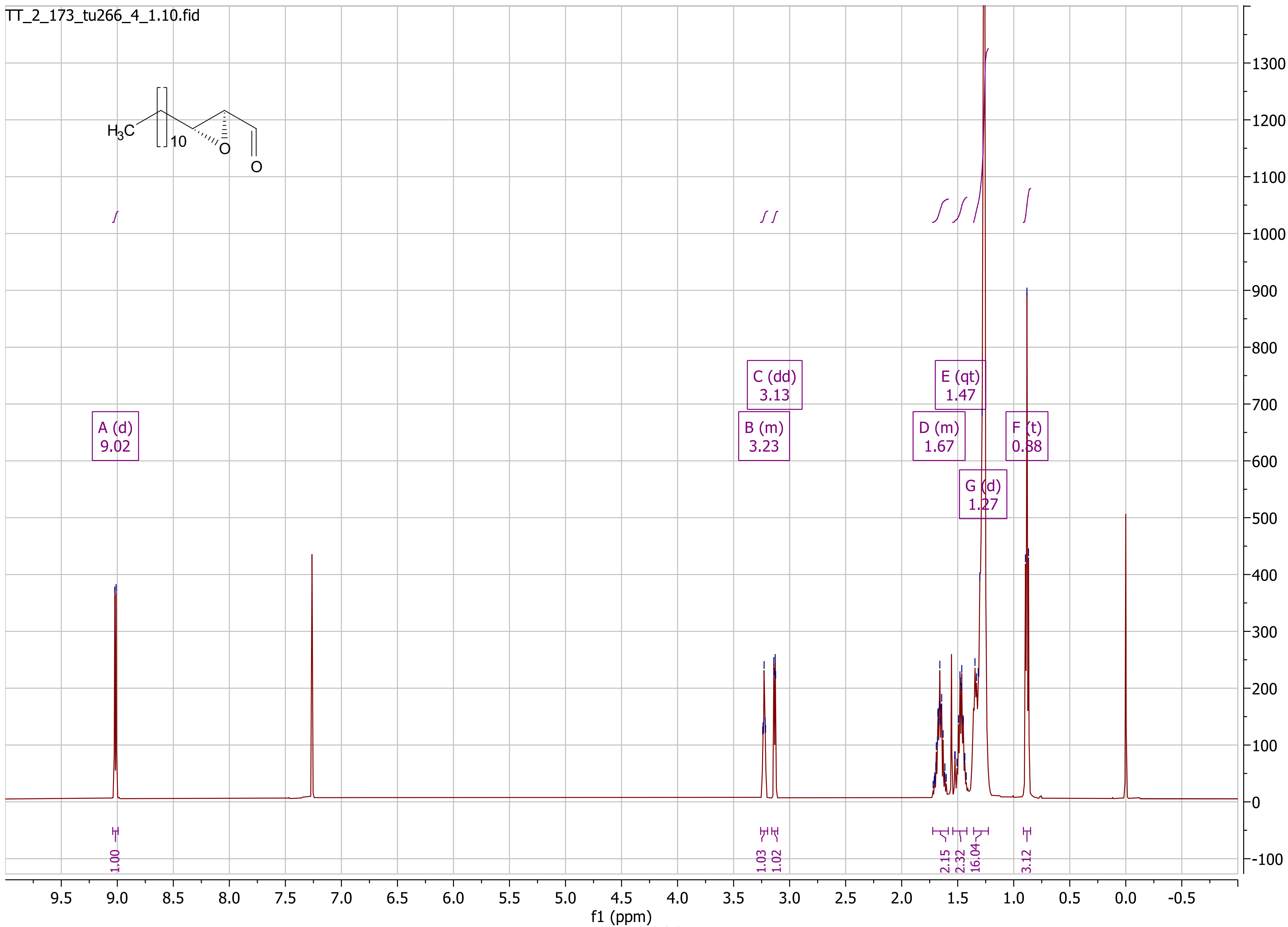

TT_2_173_tu266_4_1.10.fid
H3C
10
O
O
A (d)
9.02
B (m)
3.23
C (dd)
3.13
D (m)
1.67
E (qt)
1.47
G (d)
1.27
F (t)
0.88
1.00
1.03
1.02
2.15
2.32
16.04
3.12
f1 (ppm)

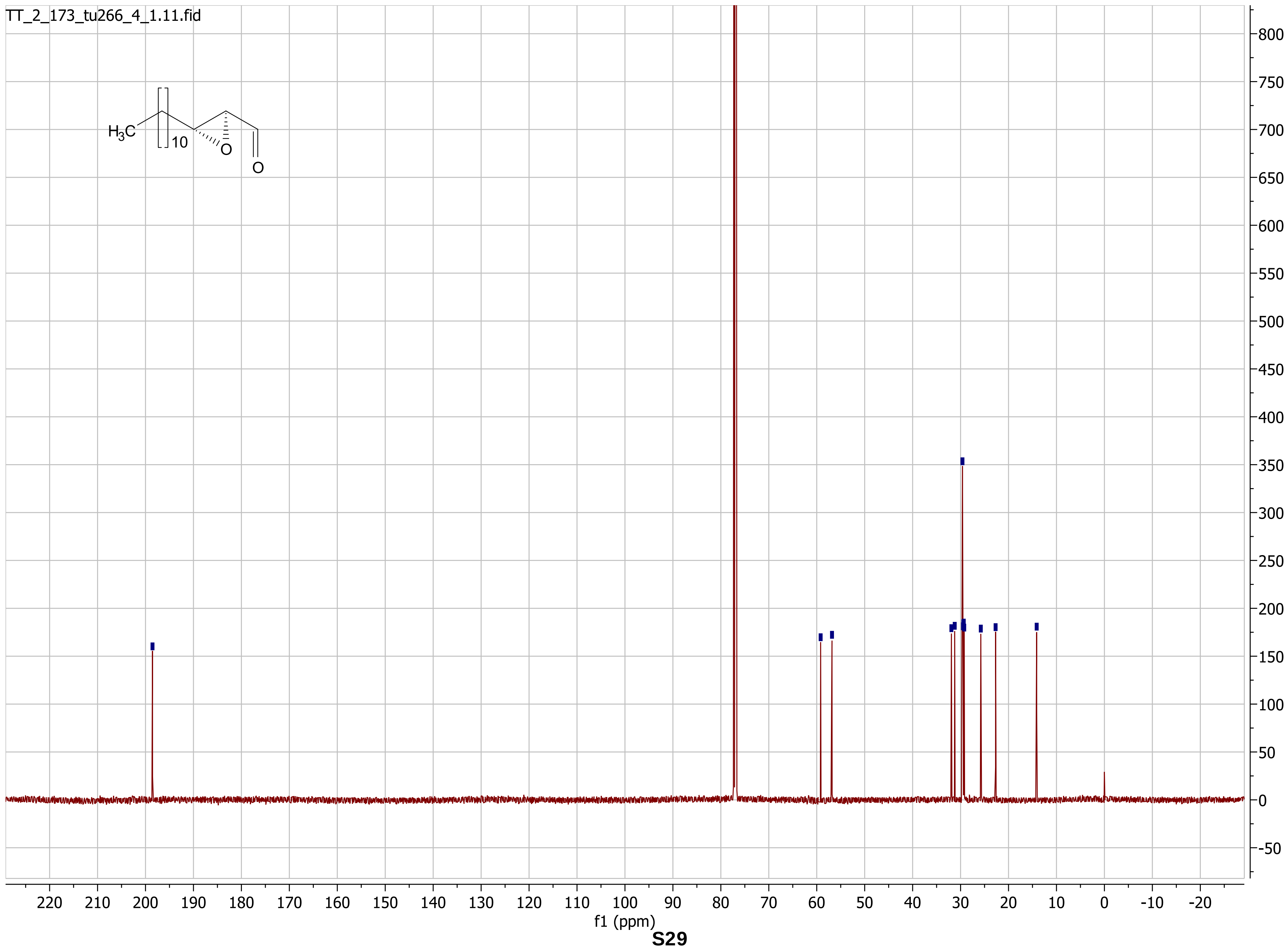

TT_2_173_tu266_4_1.11.fid
H3C
10
O
O
f1 (ppm)

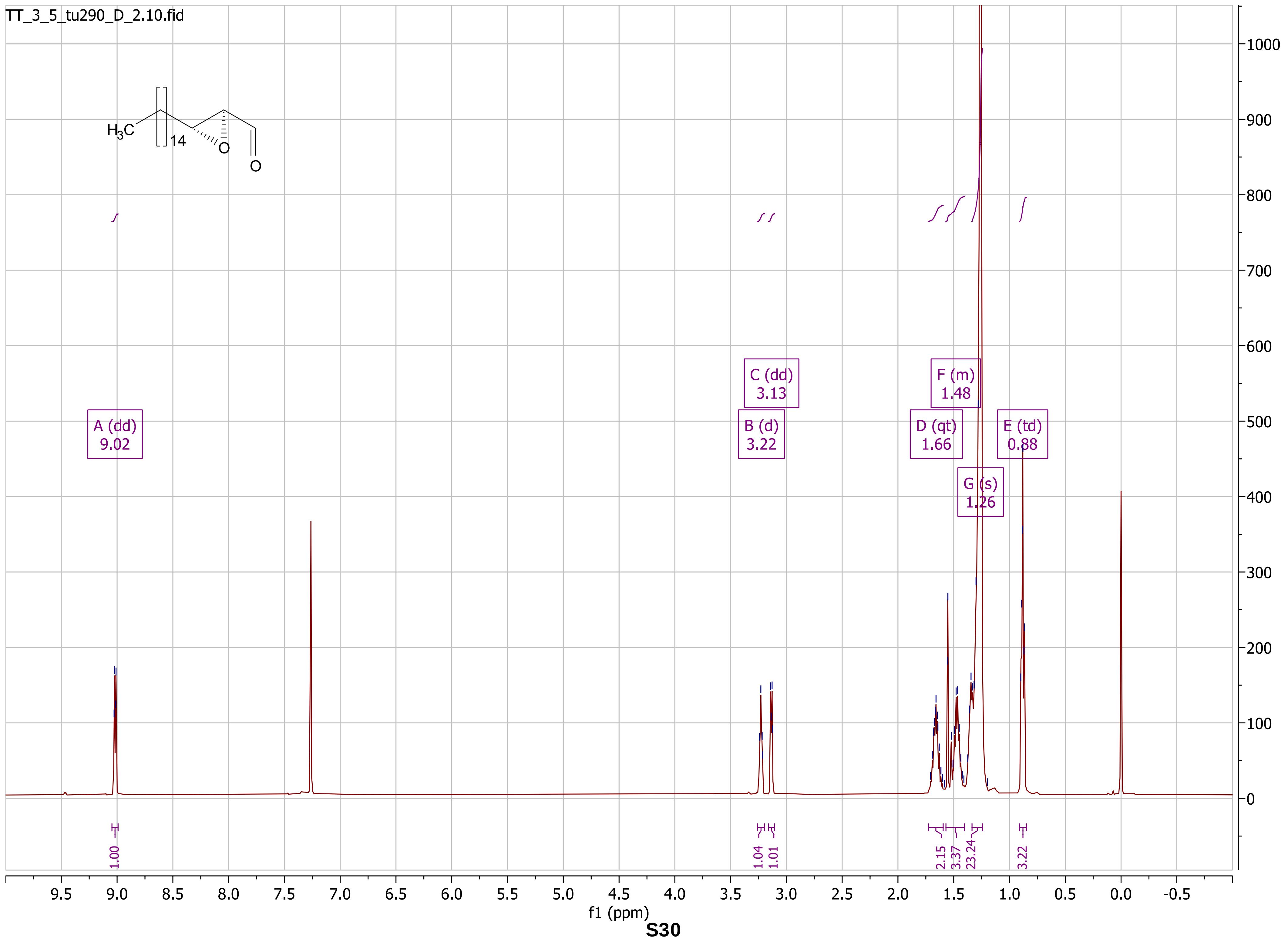
TT_3_5_tu290_D_2.10.fid
H3C
14
O
O
A (dd)
9.02
B (d)
3.22
C (dd)
3.13
D (qt)
1.66
F (m)
1.48
G (s)
1.26
E (td)
0.88
1.00
1.04
1.01
2.15
3.37
23.24
3.22
f1 (ppm)

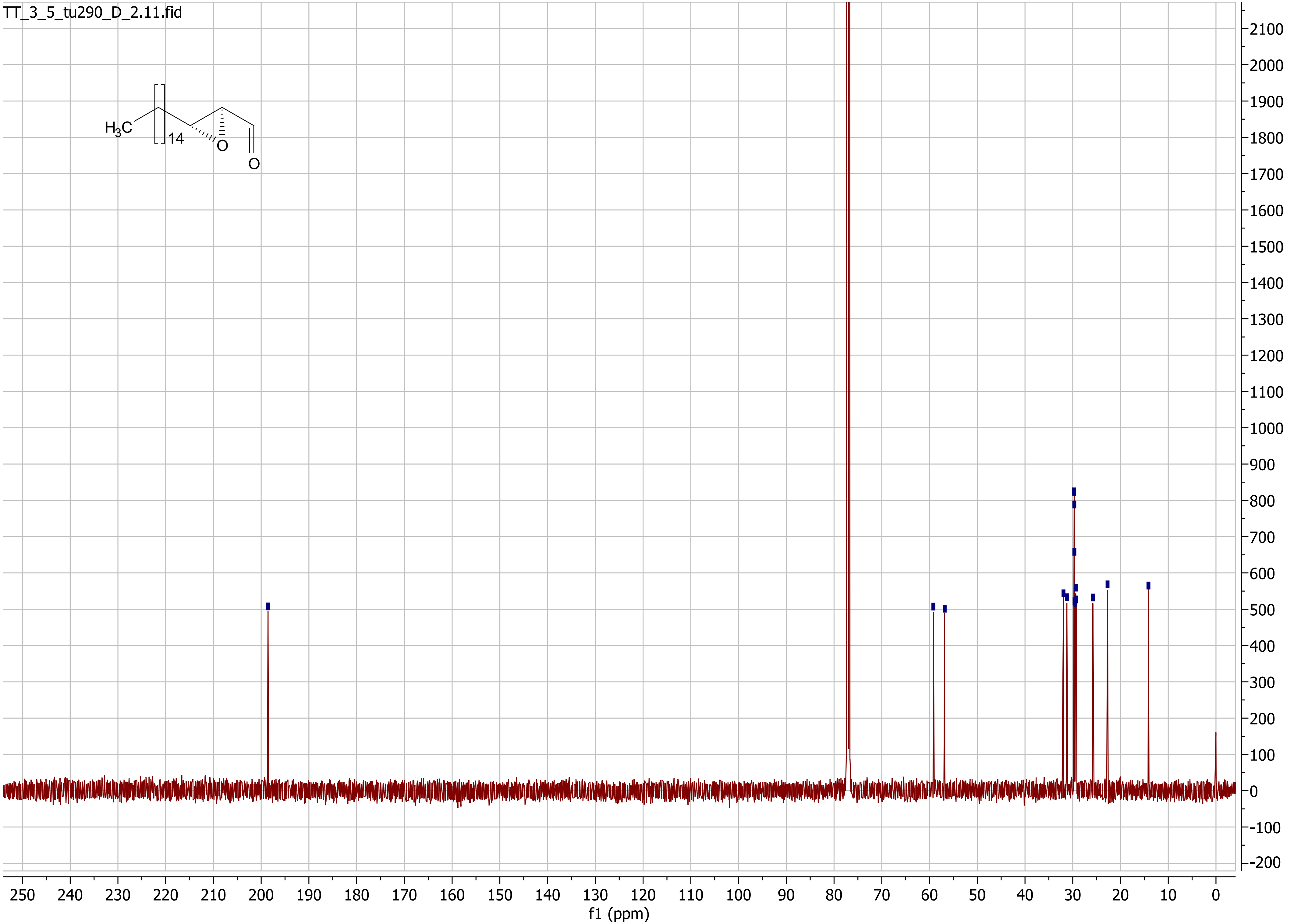
TT_3_5_tu290_D_2.11.fid
H_3C
14
O
O
f1 (ppm)

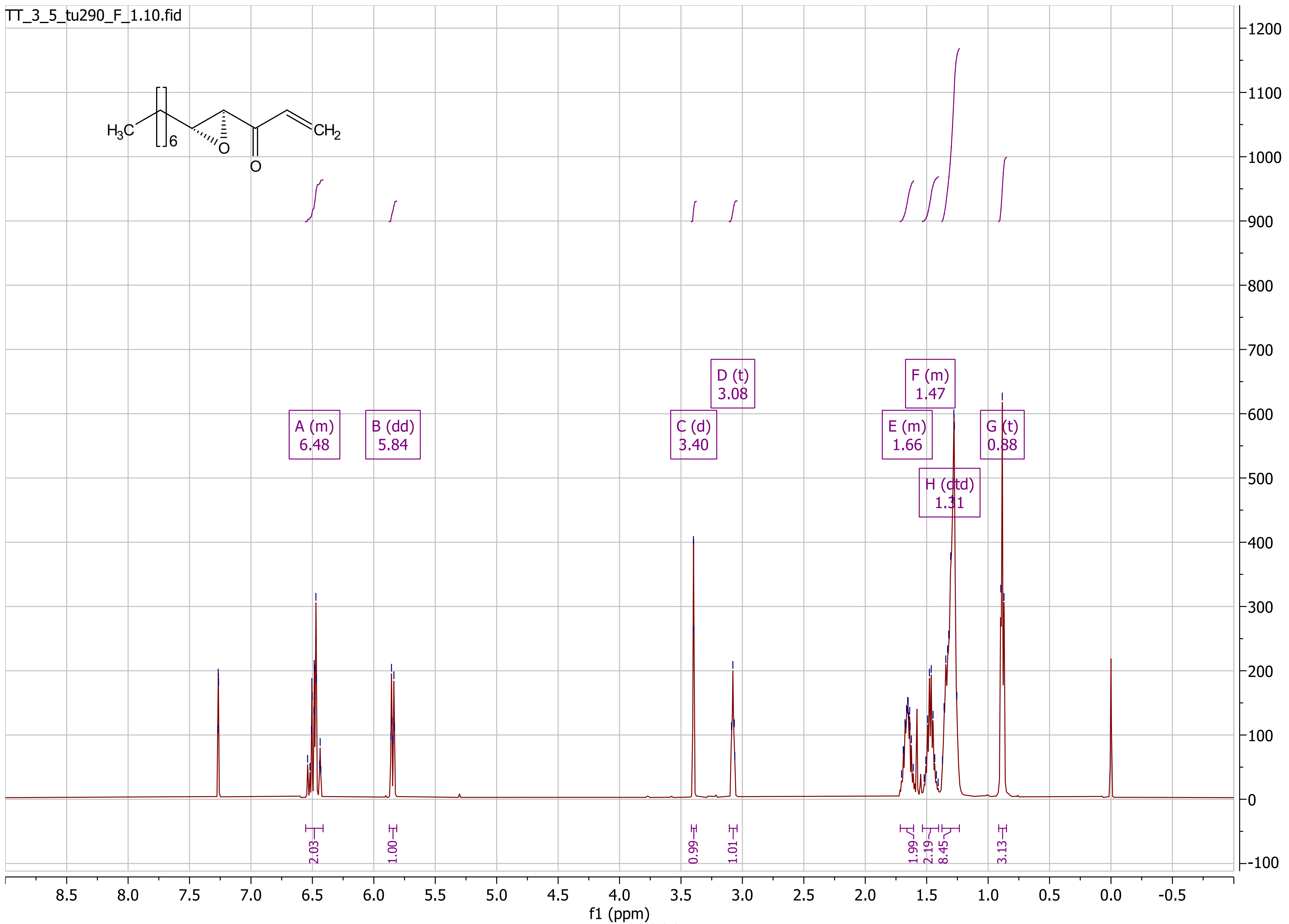
TT_3_5_tu290_F_1.10.fid
H3C
6
O
O
CH2
A (m)
6.48
B (dd)
5.84
C (d)
3.40
D (t)
3.08
E (m)
1.66
F (m)
1.47
H (qtd)
1.31
G (t)
0.88
2.03
1.00
0.99
1.01
1.99
2.19
8.45
3.13
f1 (ppm)

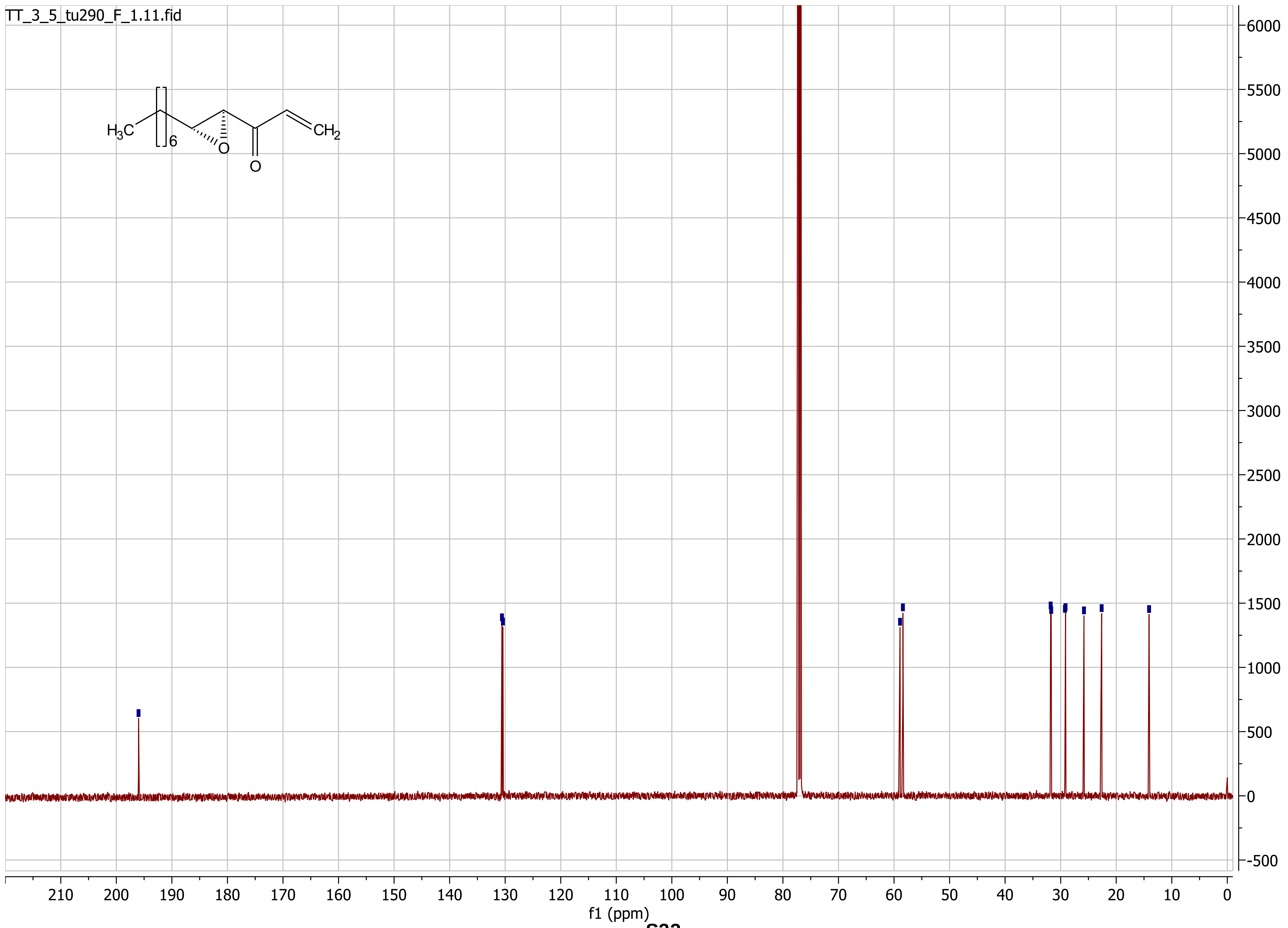

TT_3_5_tu290_F_1.11.fid
H3C
6
O
O
CH2
6000
5500
5000
4500
4000
3500
3000
2500
2000
1500
1000
500
0
-500
210 200 190 180 170 160 150 140 130 120 110 100 90 80 70 60 50 40 30 20 10 0
f1 (ppm)

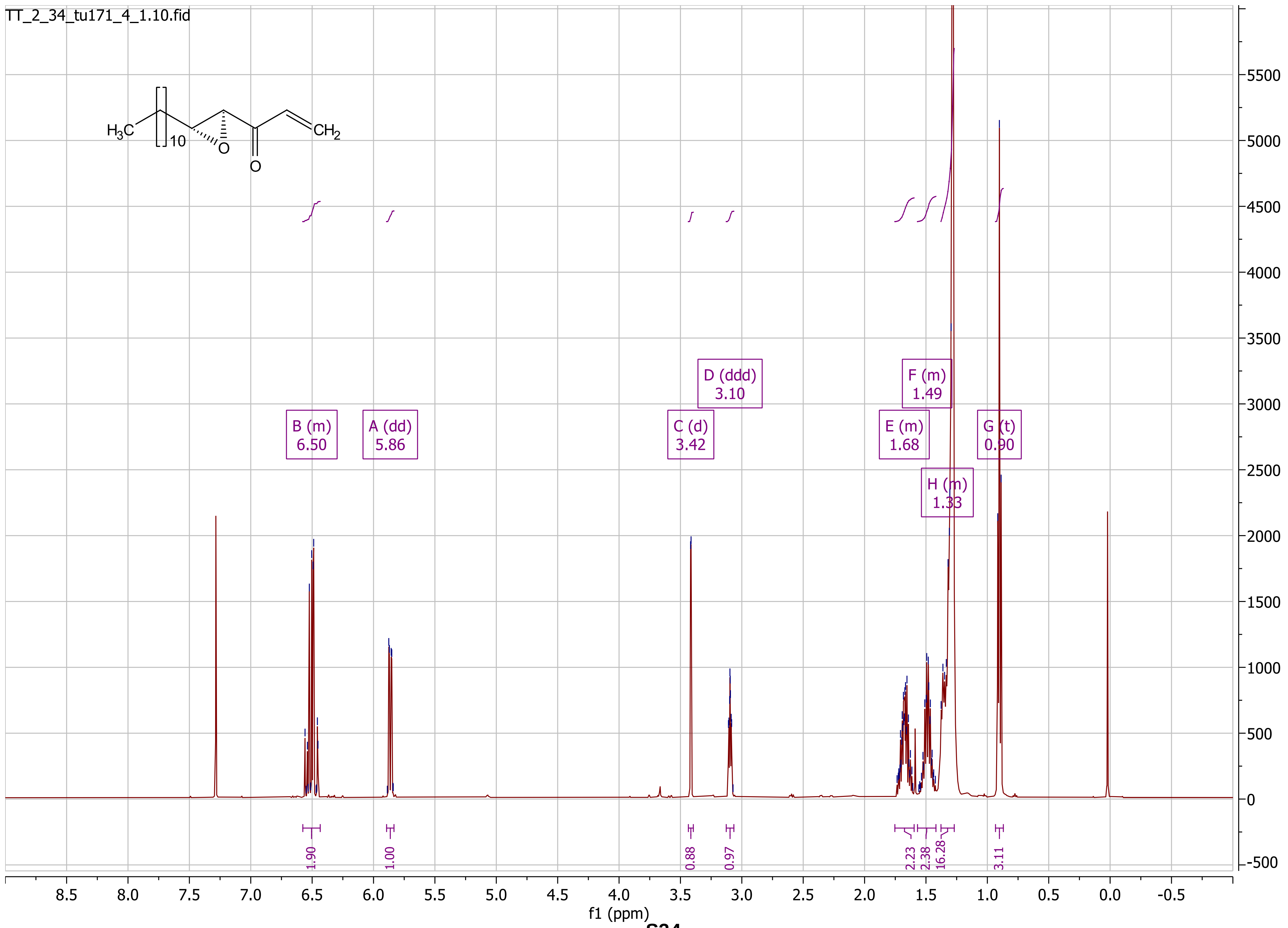

TT_2_34_tu171_4_1.10.fid
H3C
10
O
O
CH2
B (m)
6.50
A (dd)
5.86
C (d)
3.42
D (ddd)
3.10
E (m)
1.68
F (m)
1.49
H (m)
1.33
G (t)
0.90
1.90
1.00
0.88
0.97
2.23
2.38
16.28
3.11
f1 (ppm)

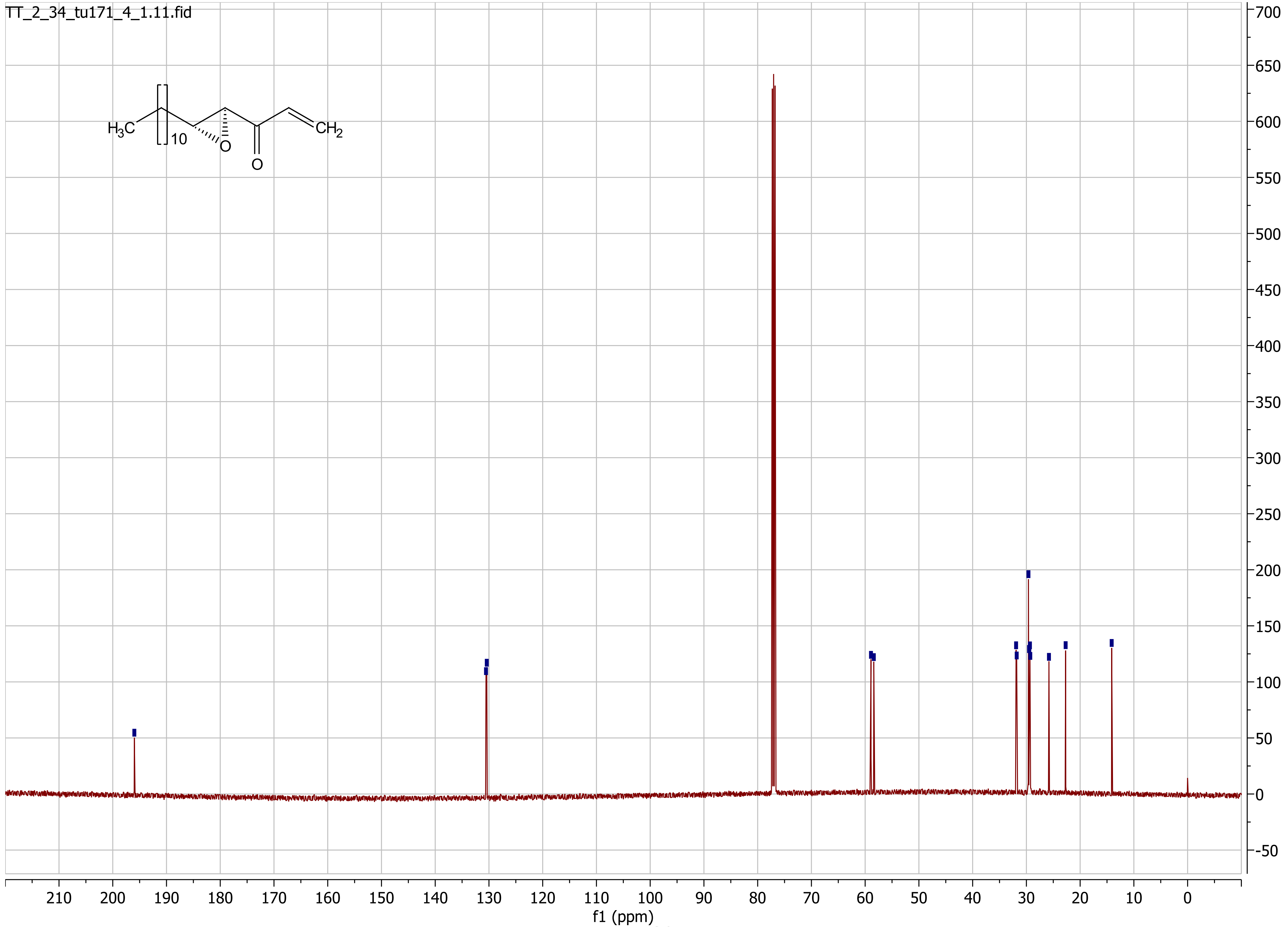

TT_2_34_tu171_4_1.11.fid
H3C
10
O
O
CH2
f1 (ppm)

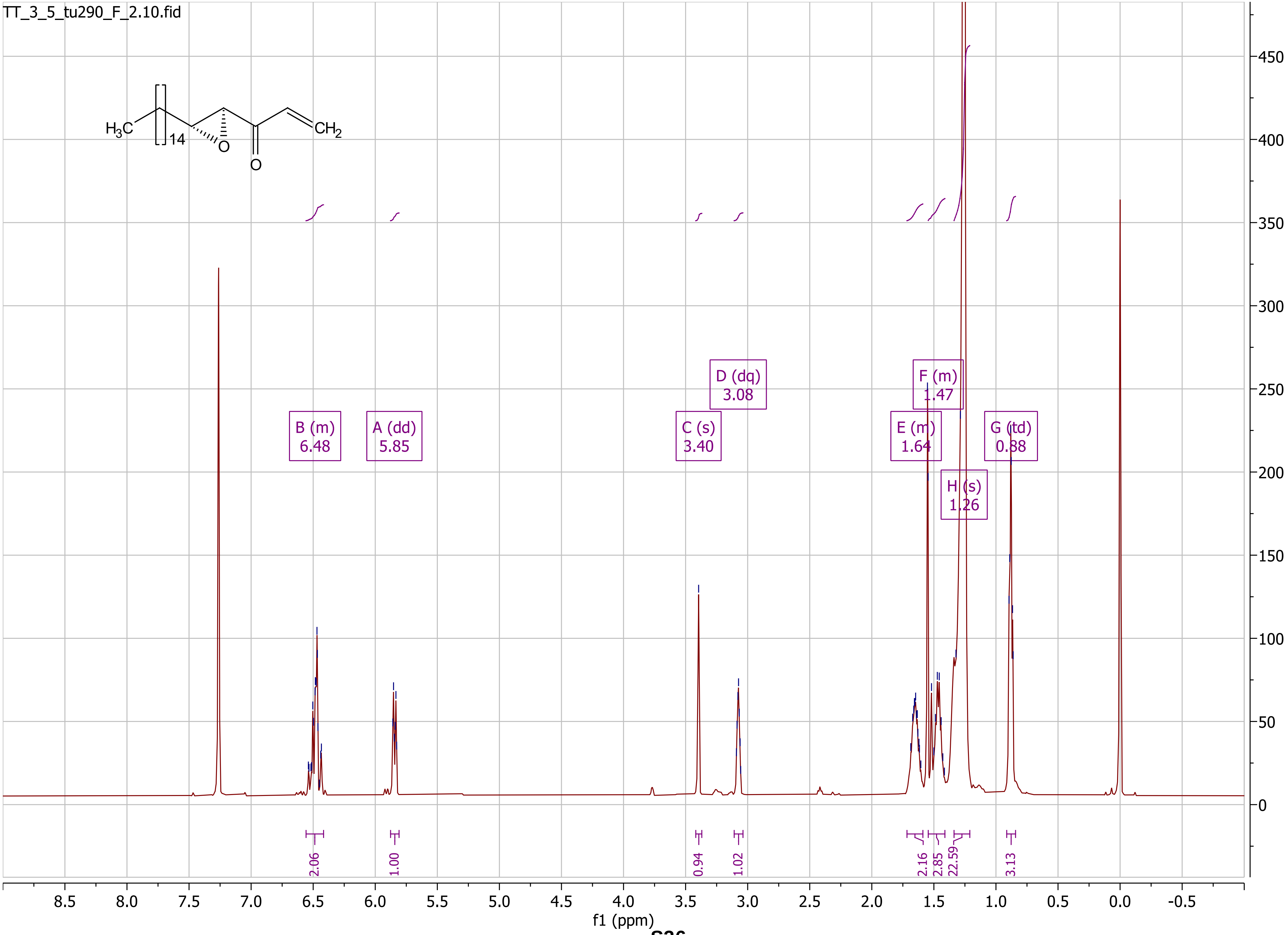
TT_3_5_tu290_F_2.10.fid
H3C
14
O
O
CH2
B (m)
6.48
A (dd)
5.85
C (s)
3.40
D (dq)
3.08
E (m)
1.64
F (m)
1.47
H (s)
1.26
G (td)
0.88
2.06
1.00
0.94
1.02
2.16
2.85
22.59
3.13
f1 (ppm)

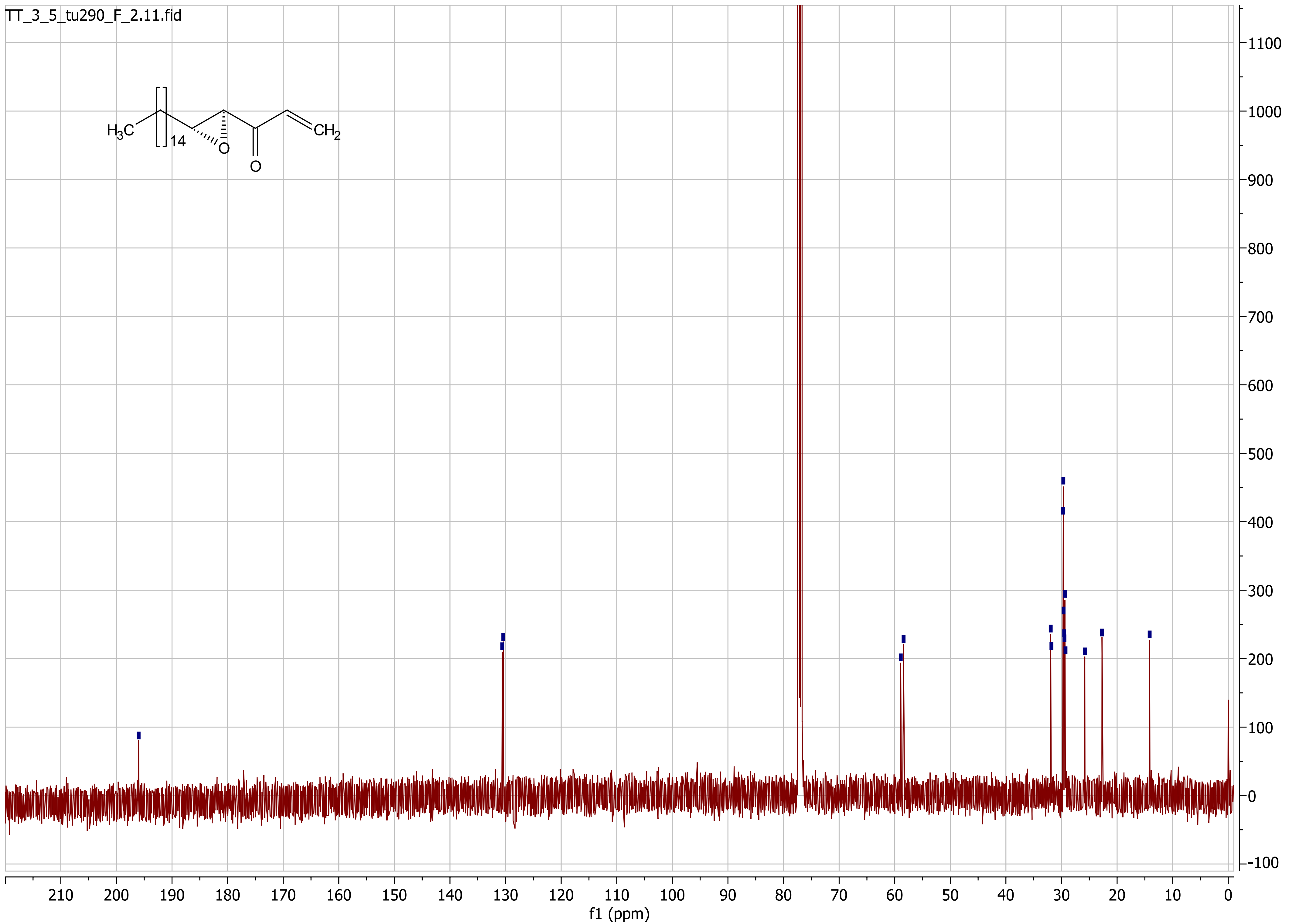
TT_3_5_tu290_F_2.11.fid
H3C
14
O
O
CH2
1100
1000
900
800
700
600
500
400
300
200
100
0
-100
210
200
190
180
170
160
150
140
130
120
110
100
90
80
70
60
50
40
30
20
10
0
f1 (ppm)

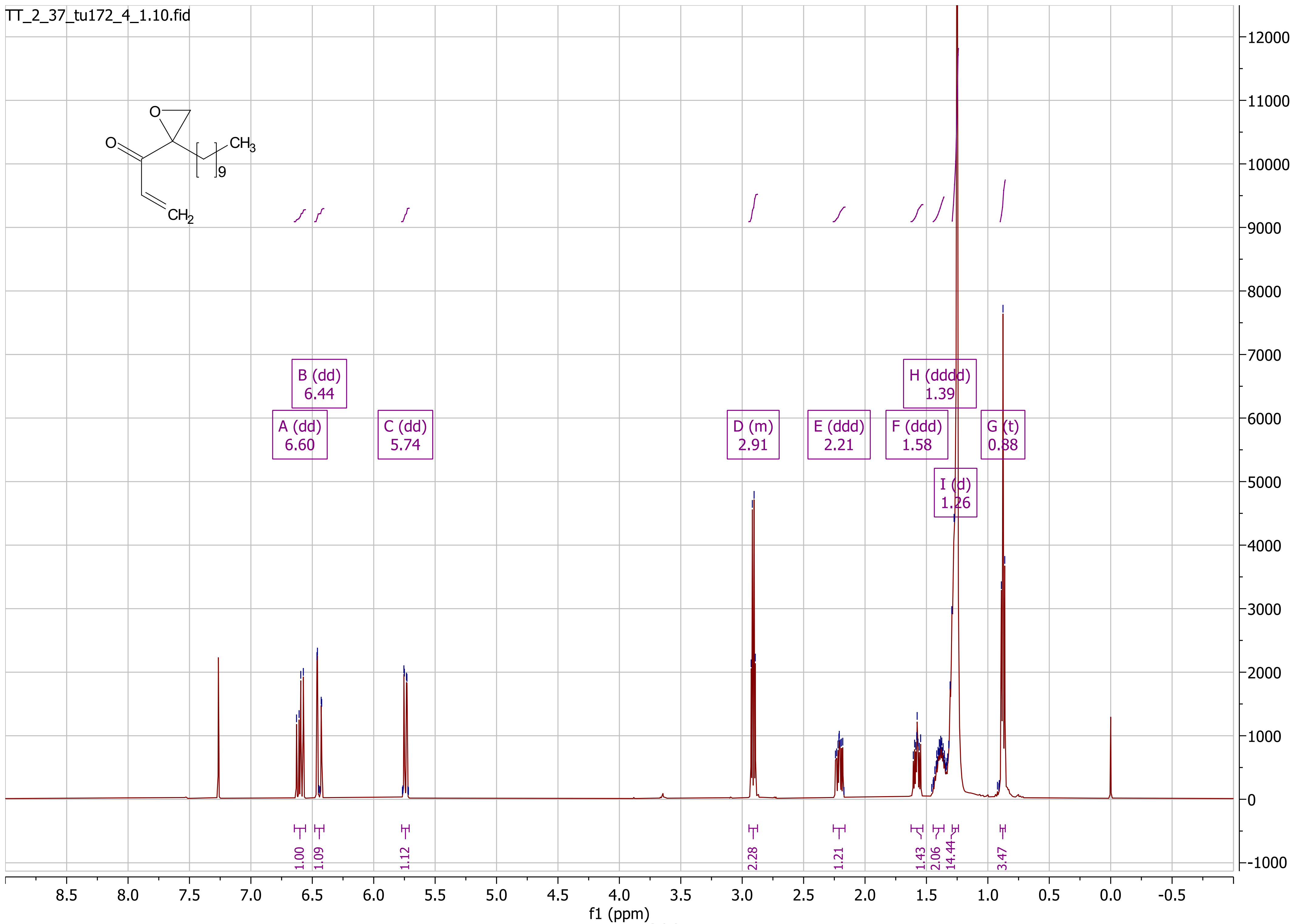
TT_2_37_tu172_4_1.10.fid
A (dd)
6.60
B (dd)
6.44
C (dd)
5.74
D (m)
2.91
E (ddd)
2.21
F (ddd)
1.58
H (dddd)
1.39
I (d)
1.26
G (t)
0.88
1.00
1.09
1.12
2.28
1.21
1.43
2.06
14.44
3.47
f1 (ppm)

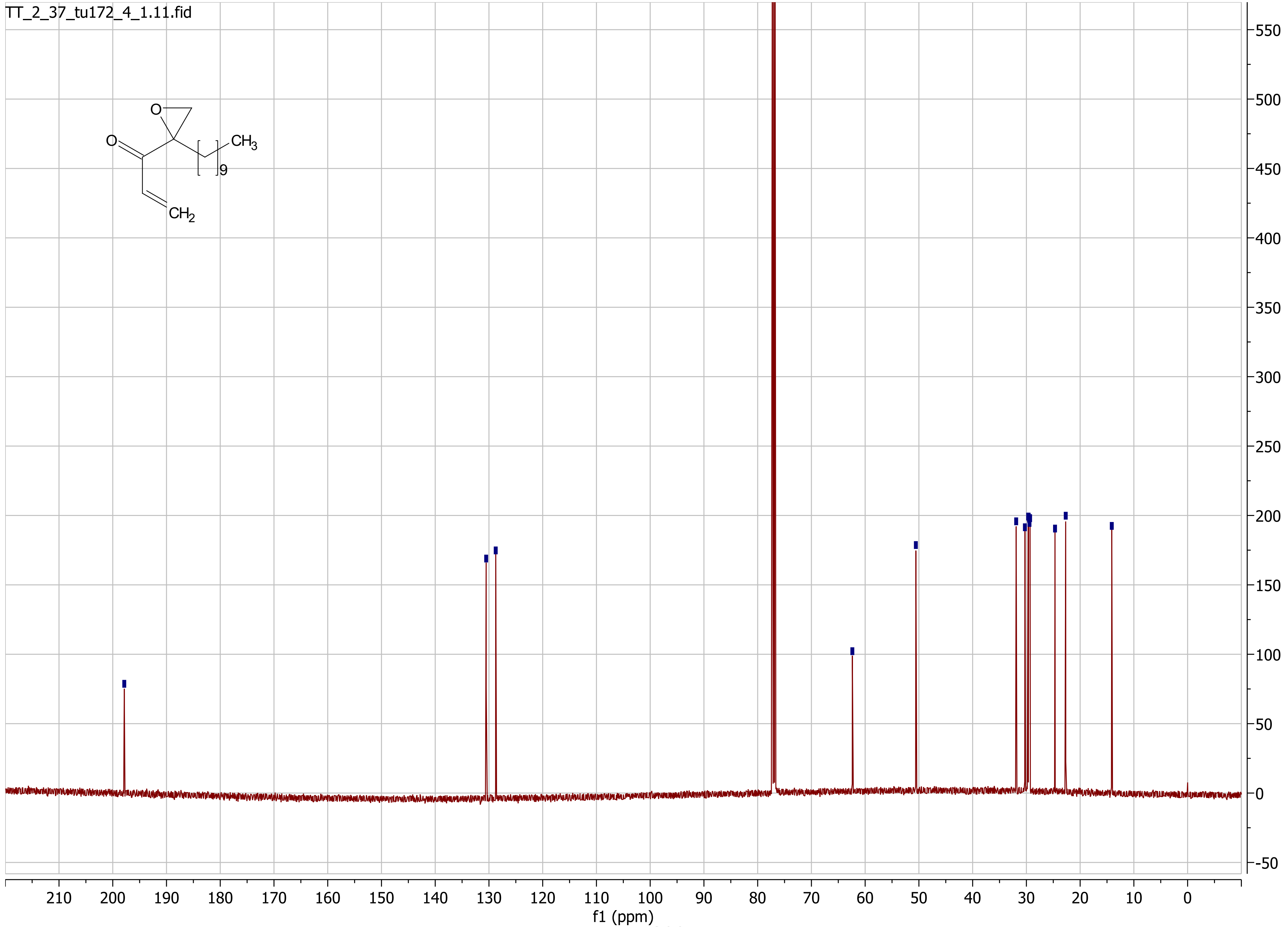
TT_2_37_tu172_4_1.11.fid
O
O
CH3
9
CH2
f1 (ppm)

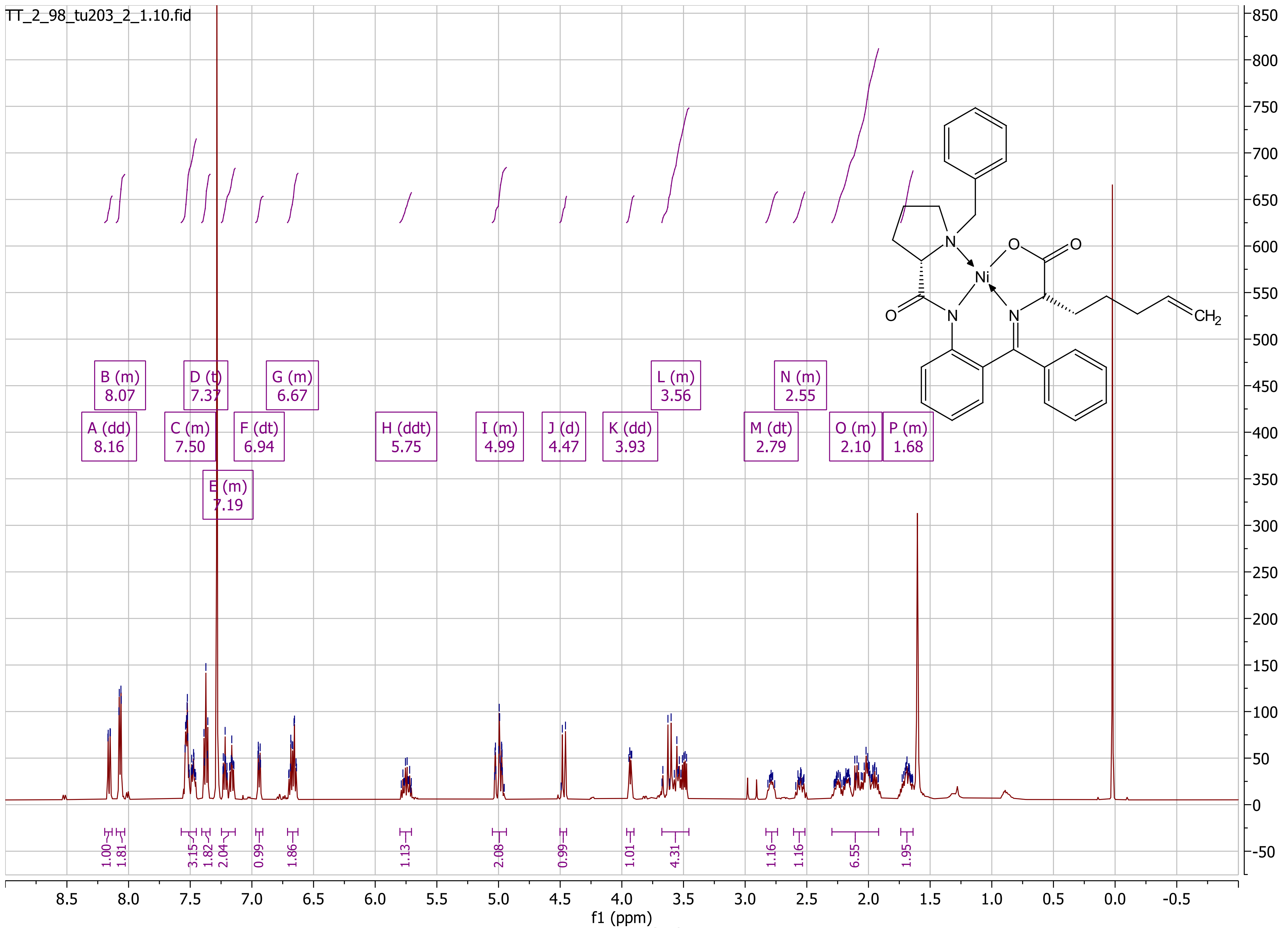
TT_2_98_tu203_2_1.10.fid
A (dd) 8.16
B (m) 8.07
C (m) 7.50
D (t) 7.37
E (m) 7.19
F (dt) 6.94
G (m) 6.67
H (ddt) 5.75
I (m) 4.99
J (d) 4.47
K (dd) 3.93
L (m) 3.56
M (dt) 2.79
N (m) 2.55
O (m) 2.10
P (m) 1.68
1.00 1.81 3.15 1.82 2.04 0.99 1.86 1.13 2.08 0.99 1.01 4.31 1.16 1.16 6.55 1.95
N O O Ni N N O CH2
f1 (ppm)

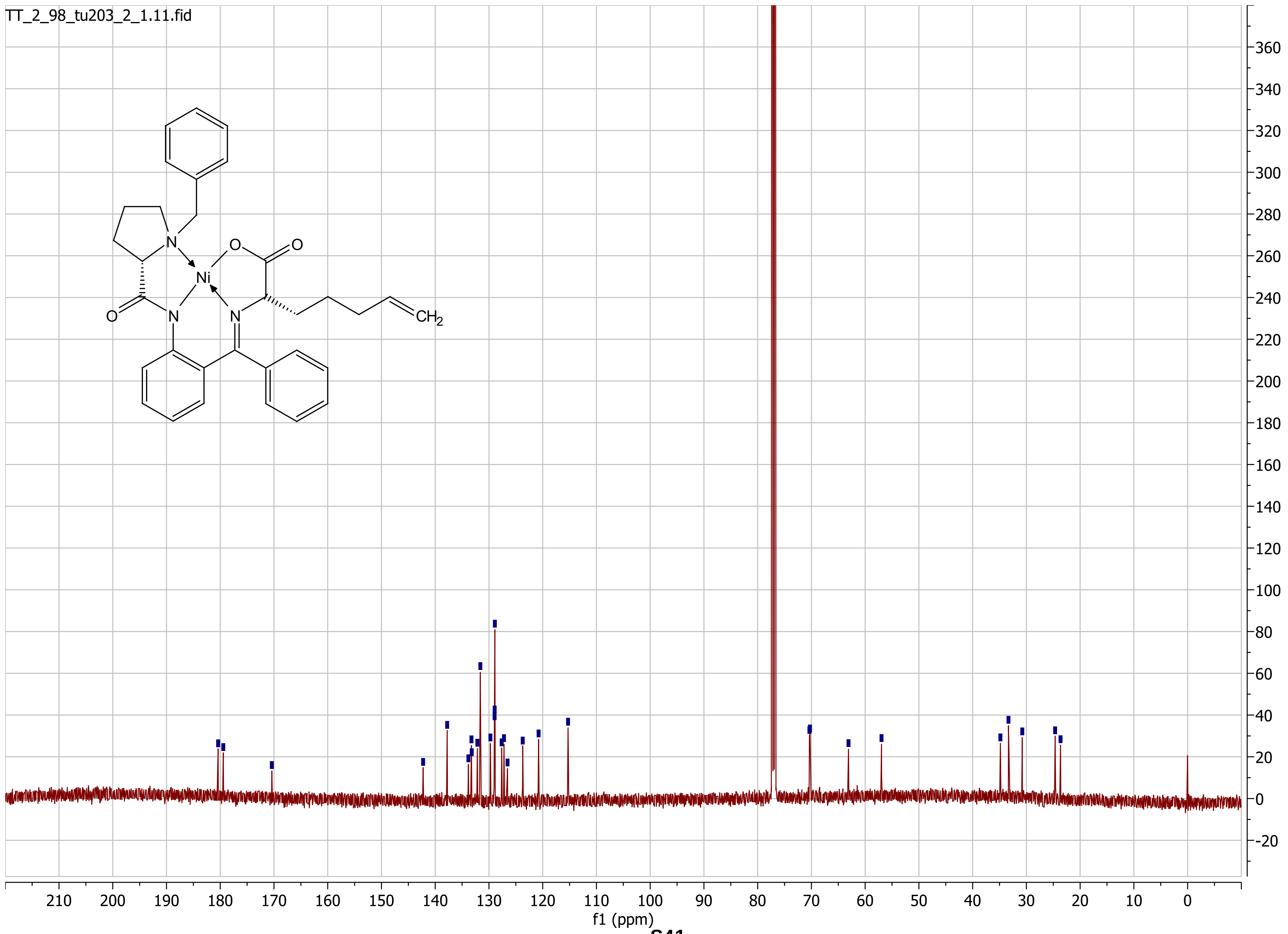
TT_2_98_tu203_2_1.11.fid
N
Ni
O
O
N
N
O
CH2
f1 (ppm)

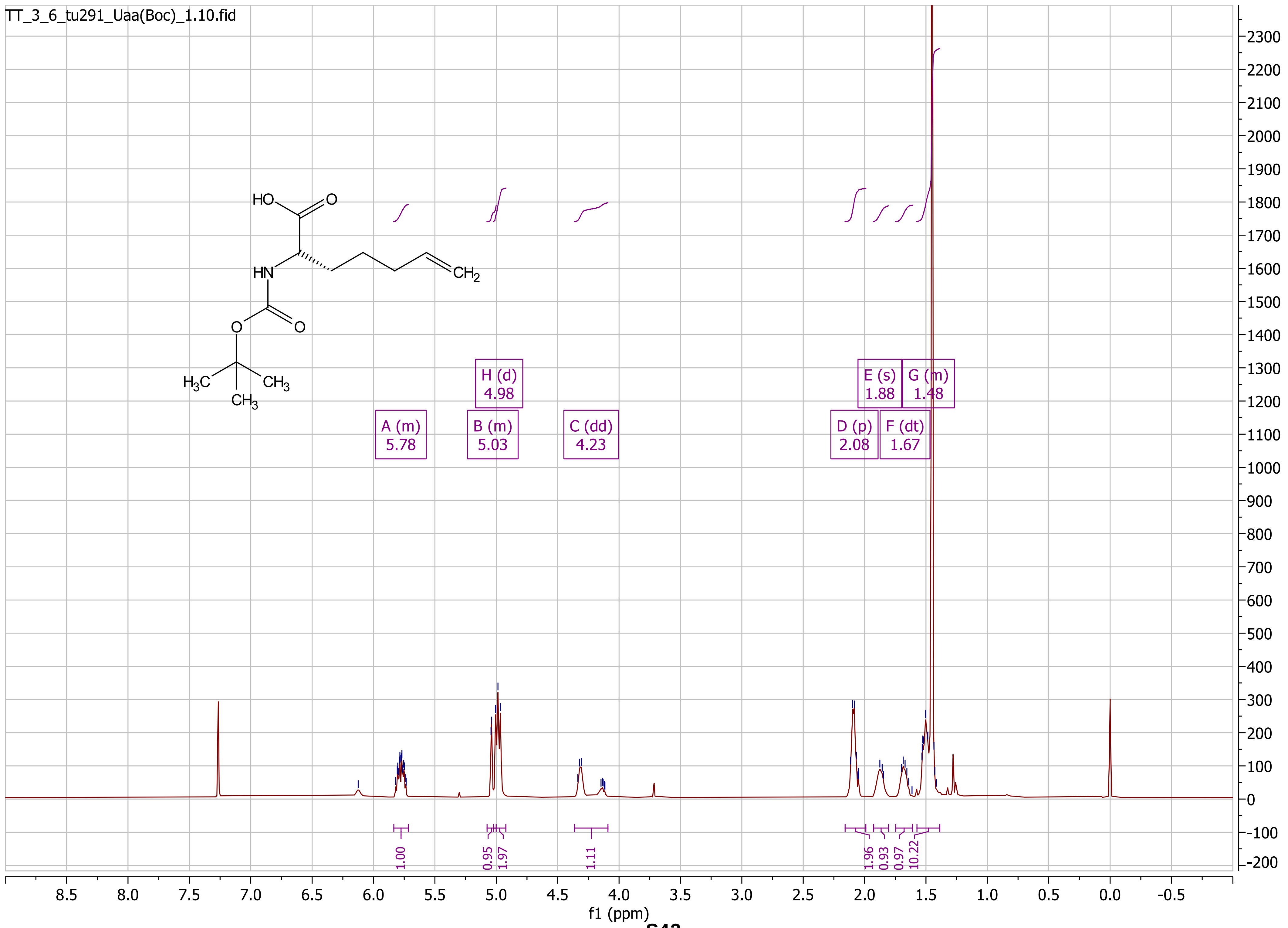
TT_3_6_tu291_Uaa(Boc)_1.10.fid
HO
O
HN
CH2
O
O
H3C
CH3
CH3
H (d)
4.98
E (s)
1.88
G (m)
1.48
A (m)
5.78
B (m)
5.03
C (dd)
4.23
D (p)
2.08
F (dt)
1.67
1.00
0.95
1.97
1.11
1.96
0.93
0.97
10.22
f1 (ppm)

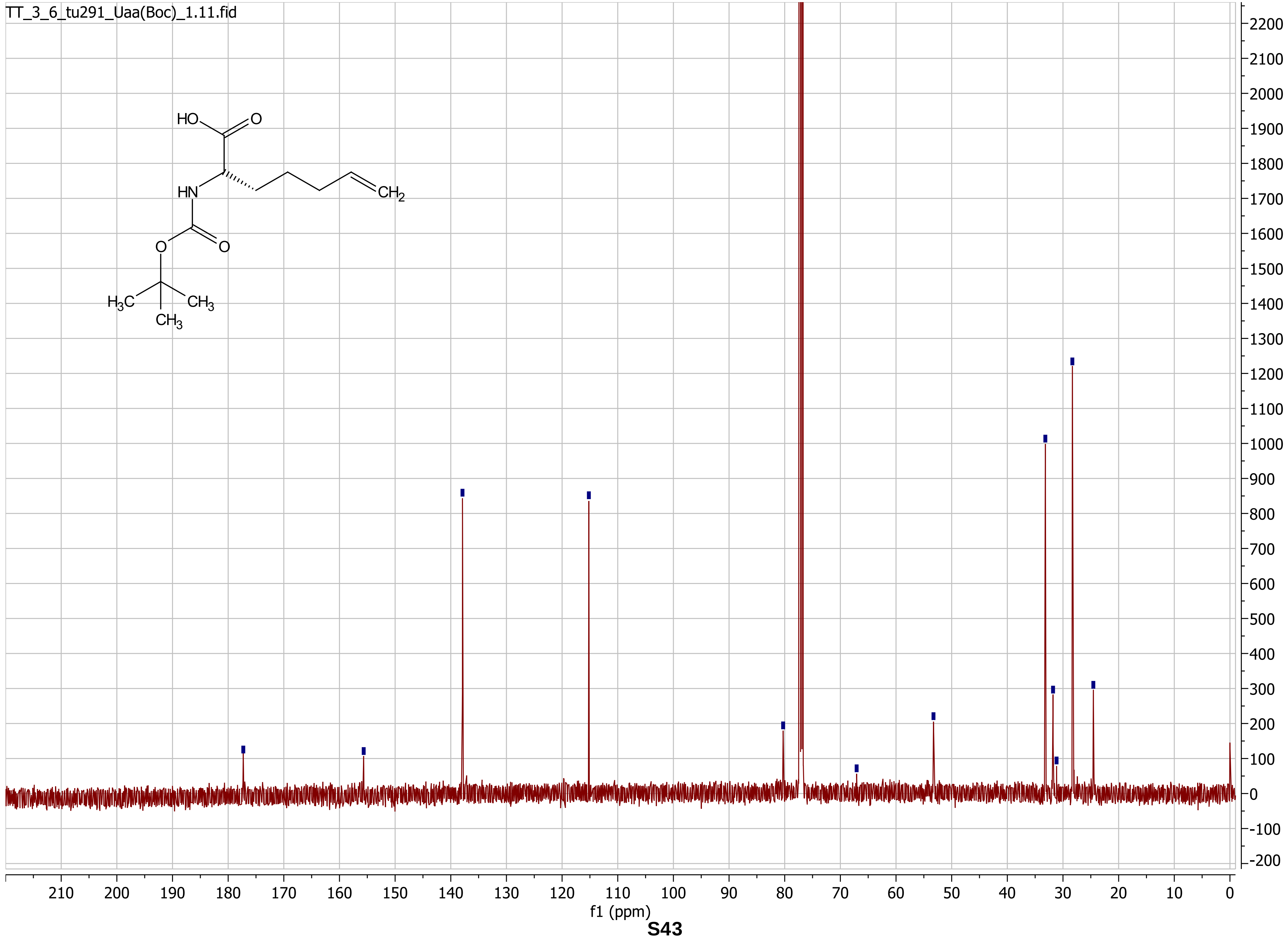
TT_3_6_tu291_Uaa(Boc)_1.11.fid
HO
O
HN
CH2
O
O
H3C
CH3
CH3
f1 (ppm)

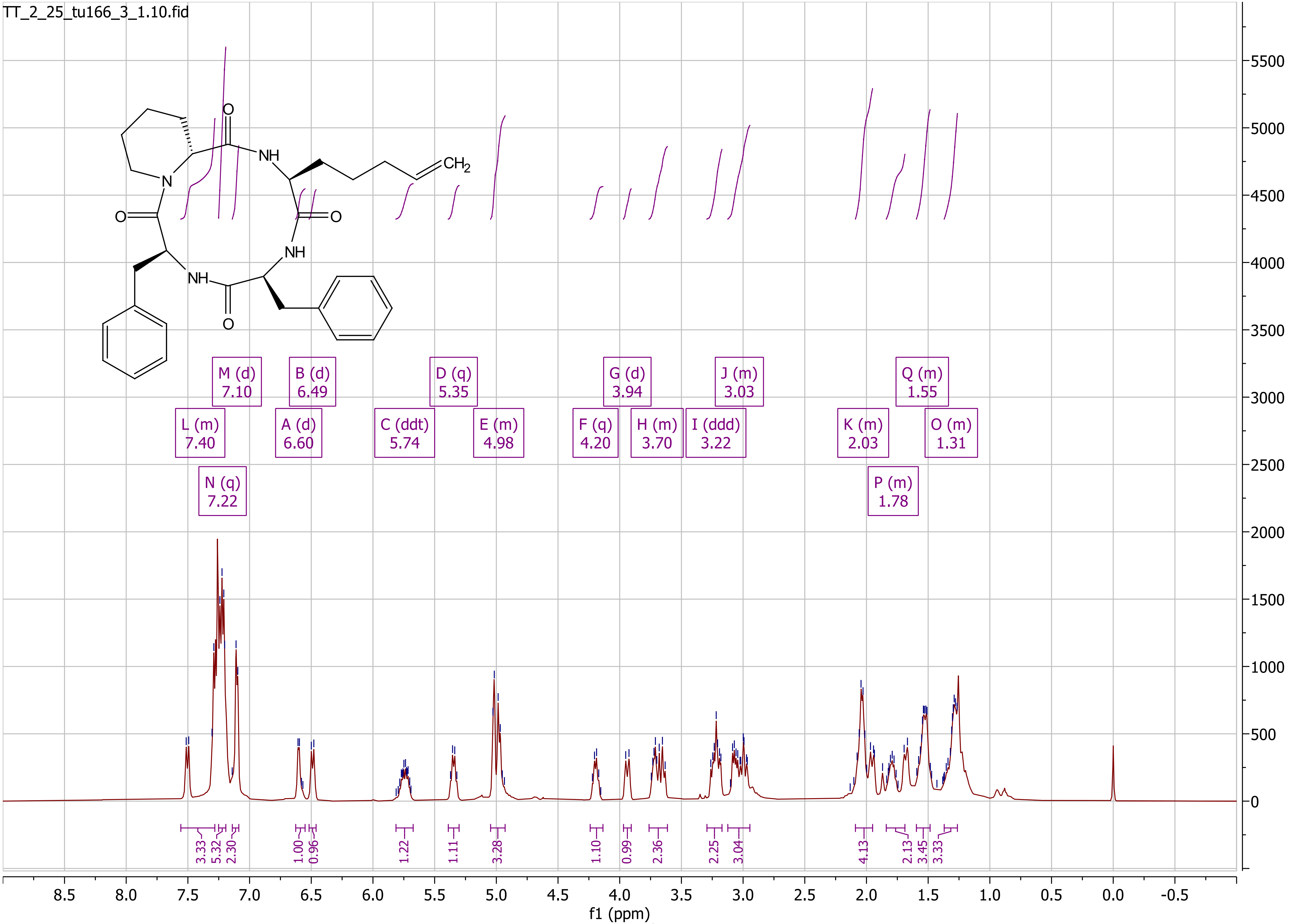
TT_2_25_tu166_3_1.10.fid
M (d) 7.10
B (d) 6.49
D (q) 5.35
G (d) 3.94
J (m) 3.03
Q (m) 1.55
L (m) 7.40
A (d) 6.60
C (ddt) 5.74
E (m) 4.98
F (q) 4.20
H (m) 3.70
I (ddd) 3.22
K (m) 2.03
O (m) 1.31
N (q) 7.22
P (m) 1.78
3.33
5.32
2.30
1.00
0.96
1.22
1.11
3.28
1.10
0.99
2.36
2.25
3.04
4.13
2.13
3.45
3.33
f1 (ppm)

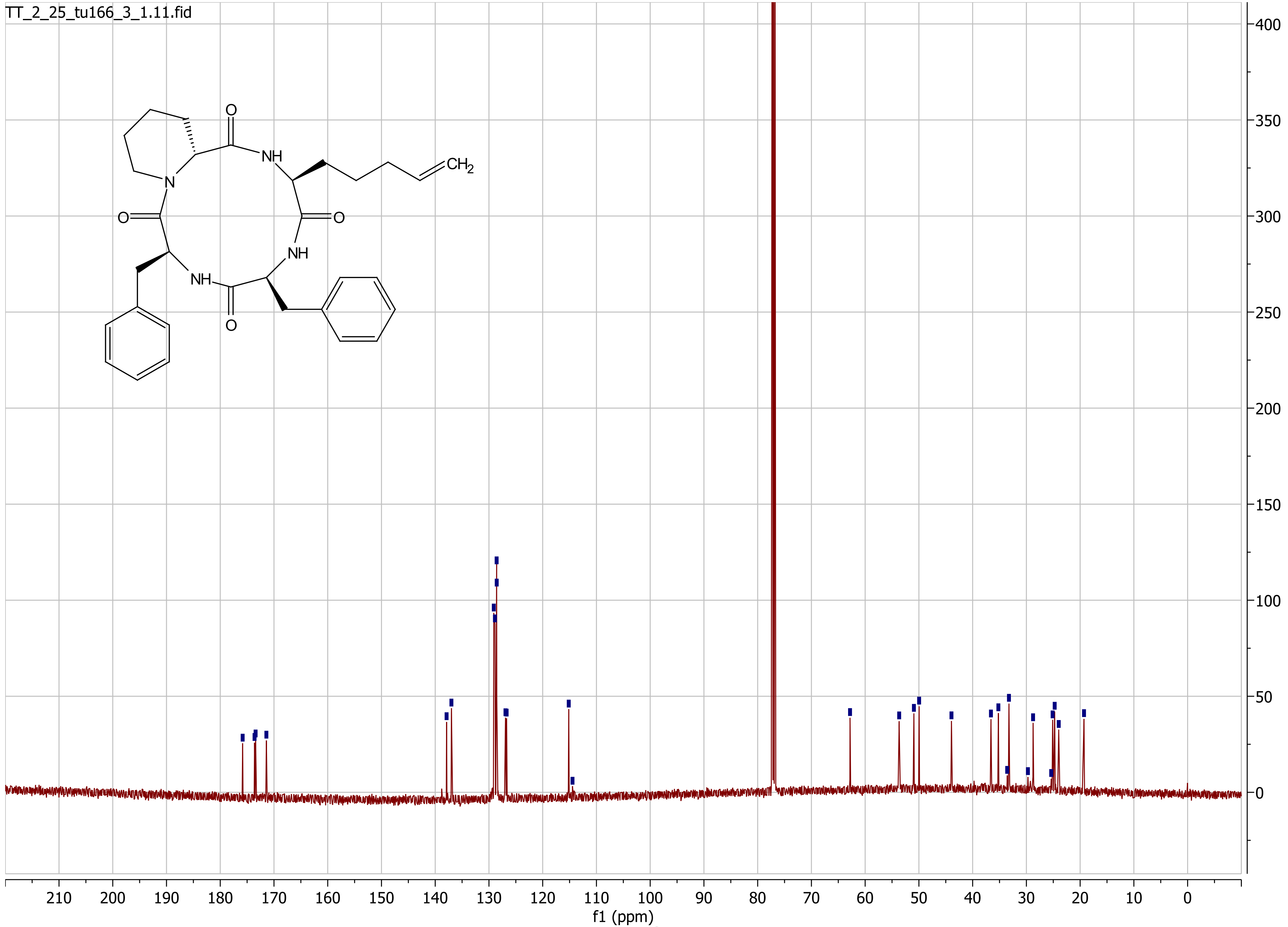
TT_2_25_tu166_3_1.11.fid
f1 (ppm)

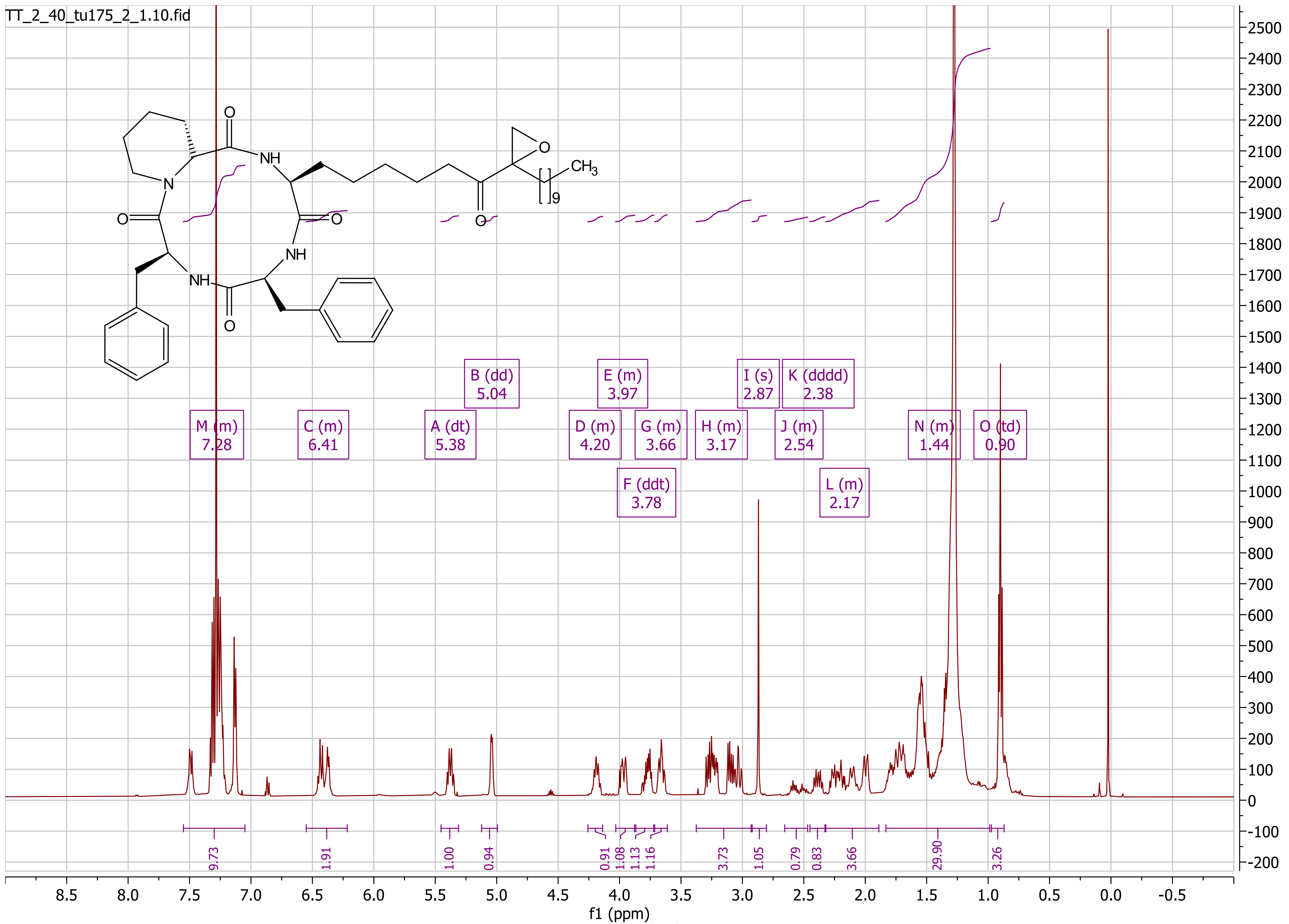
TT_2_40_tu175_2_1.10.fid
M (m) 7.28
C (m) 6.41
A (dt) 5.38
B (dd) 5.04
D (m) 4.20
E (m) 3.97
F (ddt) 3.78
G (m) 3.66
H (m) 3.17
I (s) 2.87
J (m) 2.54
K (dddd) 2.38
L (m) 2.17
N (m) 1.44
O (td) 0.90
9.73
1.91
1.00
0.94
0.91
1.08
1.13
1.16
3.73
1.05
0.79
0.83
3.66
29.90
3.26
f1 (ppm)
CH3
NH
O
N

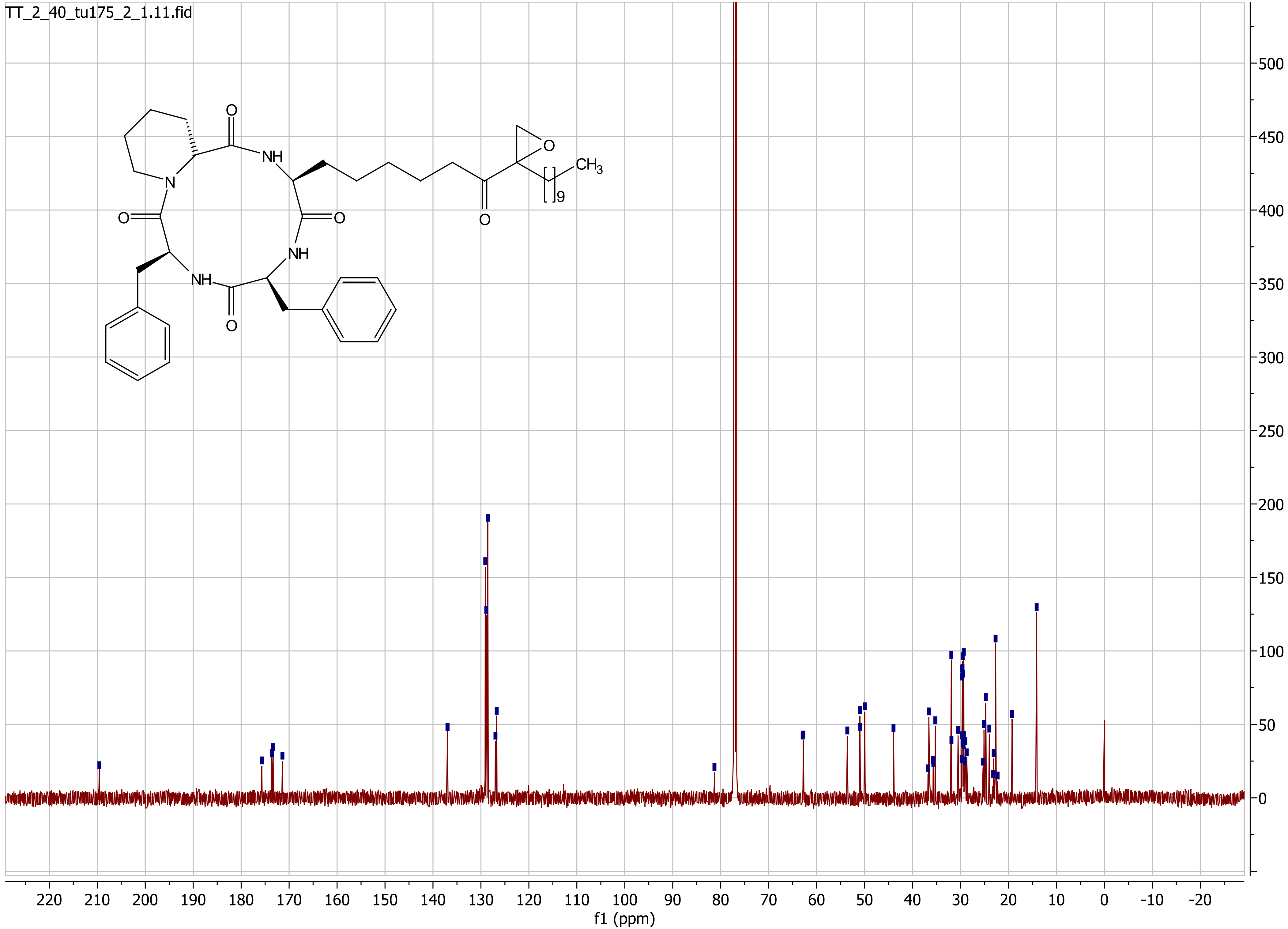
TT_2_40_tu175_2_1.11.fid
O
NH
N
O
O
NH
NH
O
O
CH3
9
O
f1 (ppm)
220 210 200 190 180 170 160 150 140 130 120 110 100 90 80 70 60 50 40 30 20 10 0 -10 -20
500 450 400 350 300 250 200 150 100 50 0

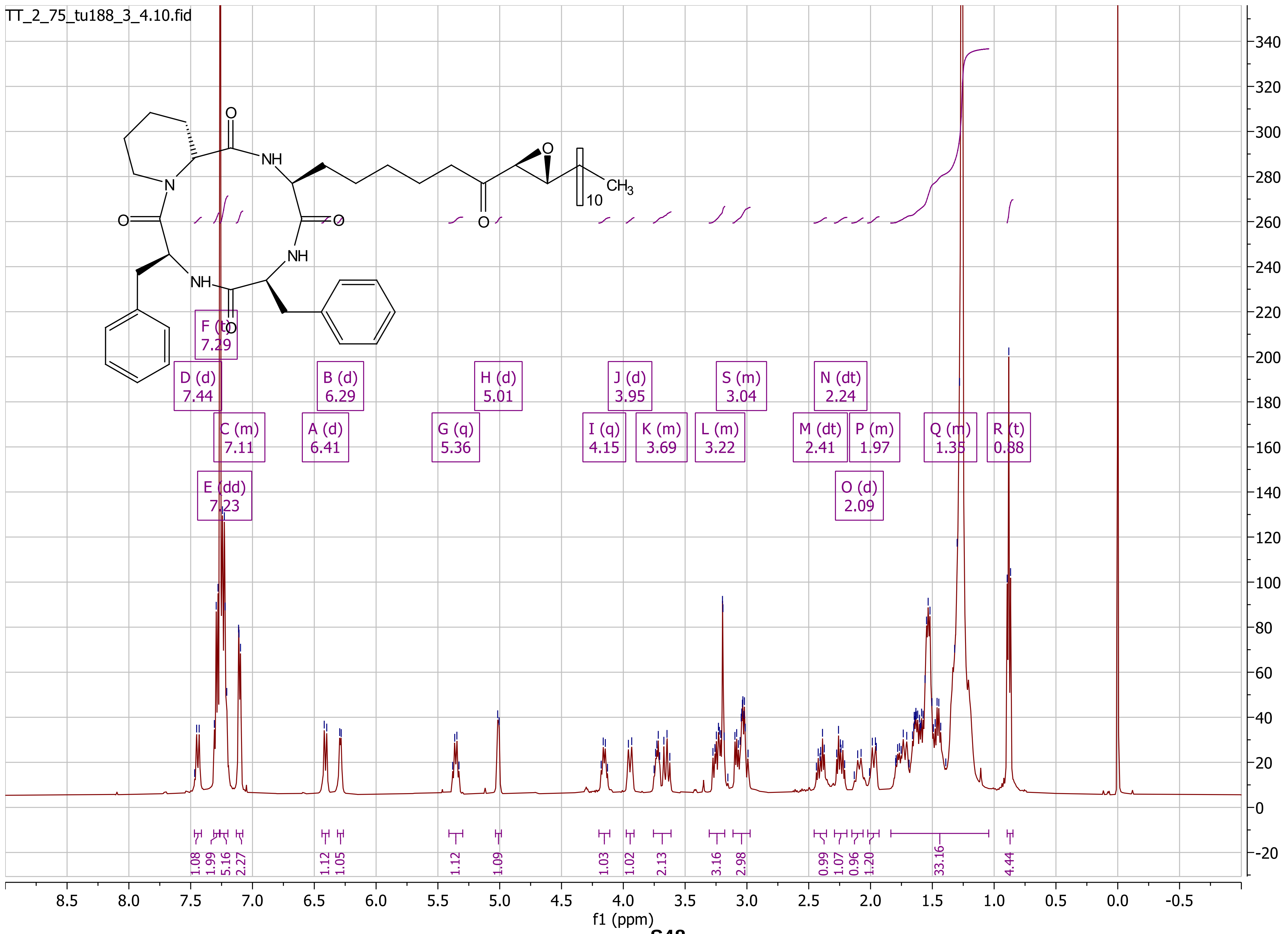
TT_2_75_tu188_3_4.10.fid
F (t) 7.29
D (d) 7.44
B (d) 6.29
H (d) 5.01
J (d) 3.95
S (m) 3.04
N (dt) 2.24
C (m) 7.11
A (d) 6.41
G (q) 5.36
I (q) 4.15
K (m) 3.69
L (m) 3.22
M (dt) 2.41
P (m) 1.97
Q (m) 1.35
R (t) 0.88
E (dd) 7.23
O (d) 2.09
1.08
1.99
5.16
2.27
1.12
1.05
1.12
1.09
1.03
1.02
2.13
3.16
2.98
0.99
1.07
0.96
1.20
33.16
4.44
f1 (ppm)

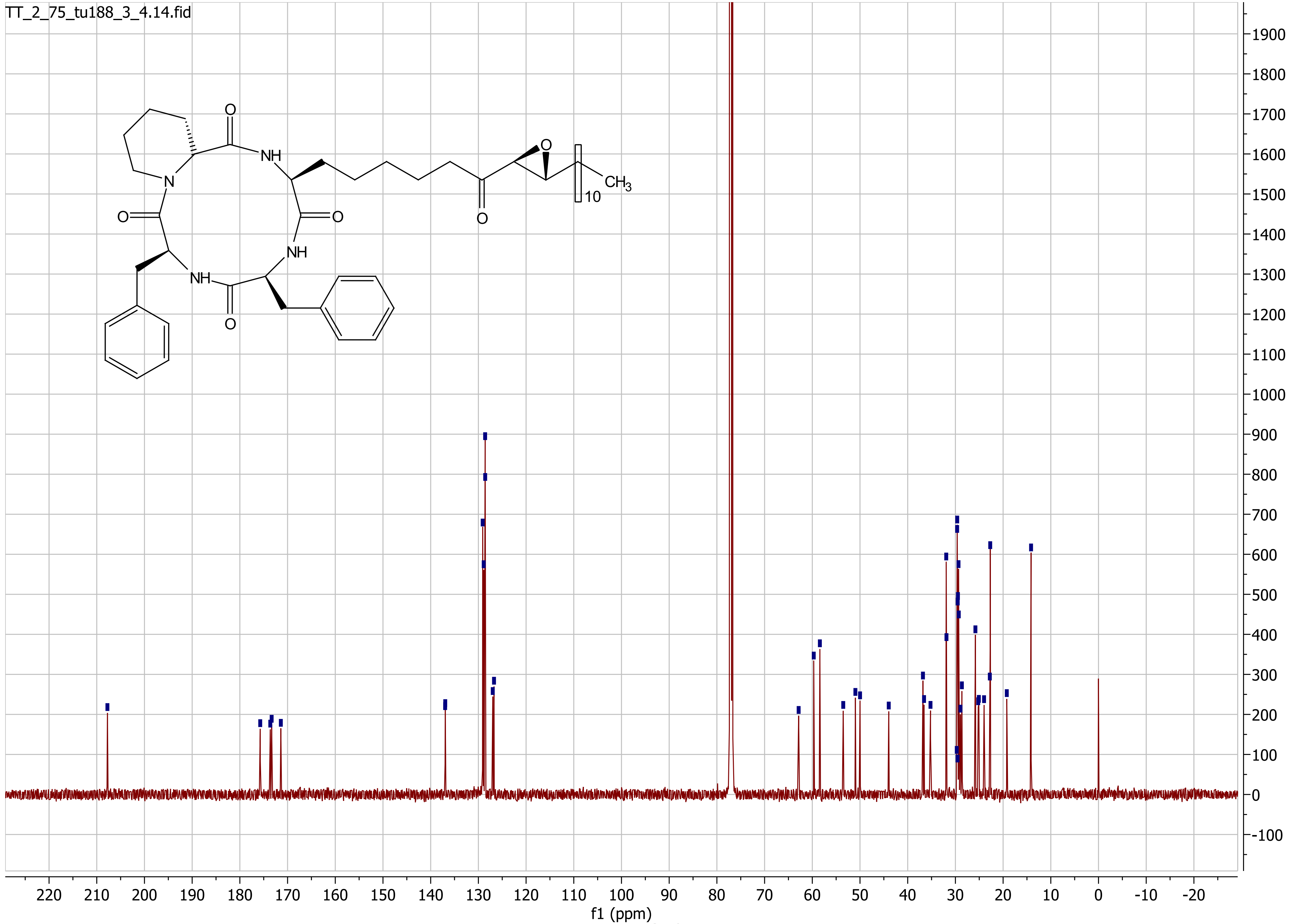
TT_2_75_tu188_3_4.14.fid
f1 (ppm)

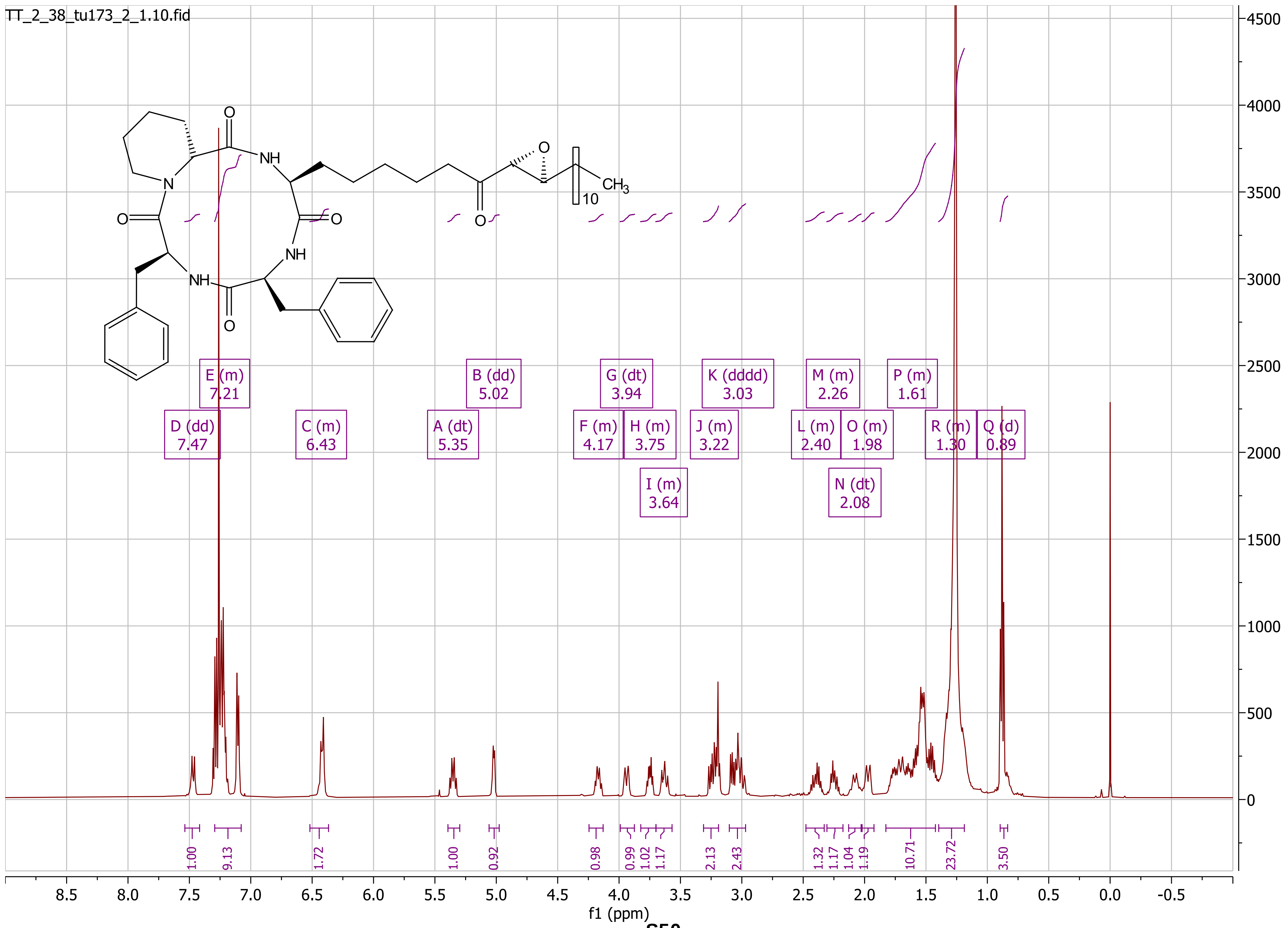
TT_2_38_tu173_2_1.10.fid
D (dd) 7.47
E (m) 7.21
C (m) 6.43
A (dt) 5.35
B (dd) 5.02
F (m) 4.17
G (dt) 3.94
H (m) 3.75
I (m) 3.64
J (m) 3.22
K (dddd) 3.03
L (m) 2.40
M (m) 2.26
N (dt) 2.08
O (m) 1.98
P (m) 1.61
R (m) 1.30
Q (d) 0.89
1.00
9.13
1.72
1.00
0.92
0.98
0.99
1.02
1.17
2.13
2.43
1.32
1.17
1.04
1.19
10.71
23.72
3.50
f1 (ppm)

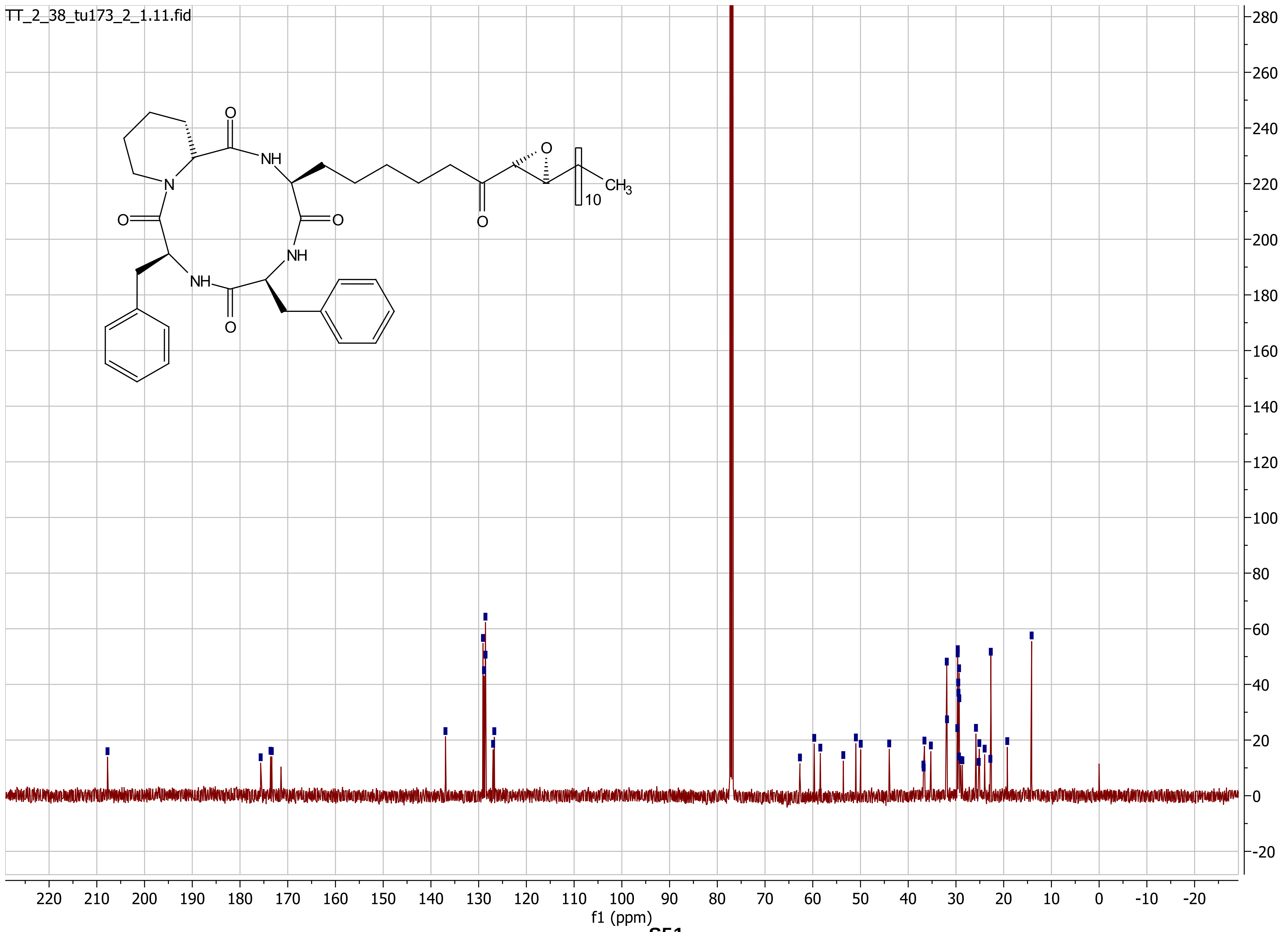
TT_2_38_tu173_2_1.11.fid
f1 (ppm)

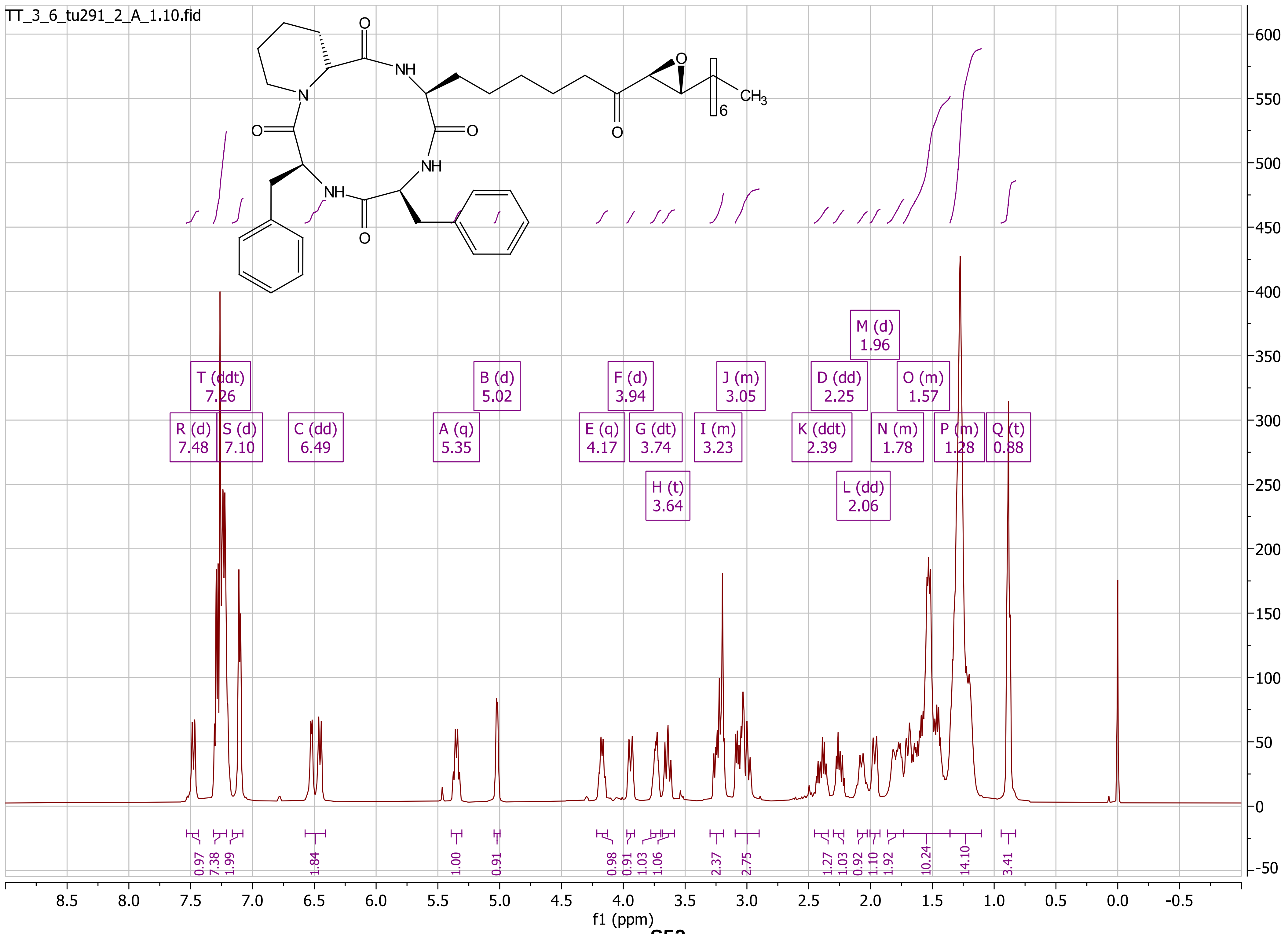

TT_3_6_tu291_2_A_1.10.fid
T (ddt) 7.26
B (d) 5.02
F (d) 3.94
J (m) 3.05
D (dd) 2.25
M (d) 1.96
O (m) 1.57
R (d) 7.48
S (d) 7.10
C (dd) 6.49
A (q) 5.35
E (q) 4.17
G (dt) 3.74
I (m) 3.23
K (ddt) 2.39
N (m) 1.78
P (m) 1.28
Q (t) 0.88
H (t) 3.64
L (dd) 2.06
0.97 7.38 1.99 1.84 1.00 0.91 0.98 0.91 1.03 1.06 2.37 2.75 1.27 1.03 0.92 1.10 1.92 10.24 14.10 3.41
f1 (ppm)

TT_3_6_tu291_2_A_1.11.fid

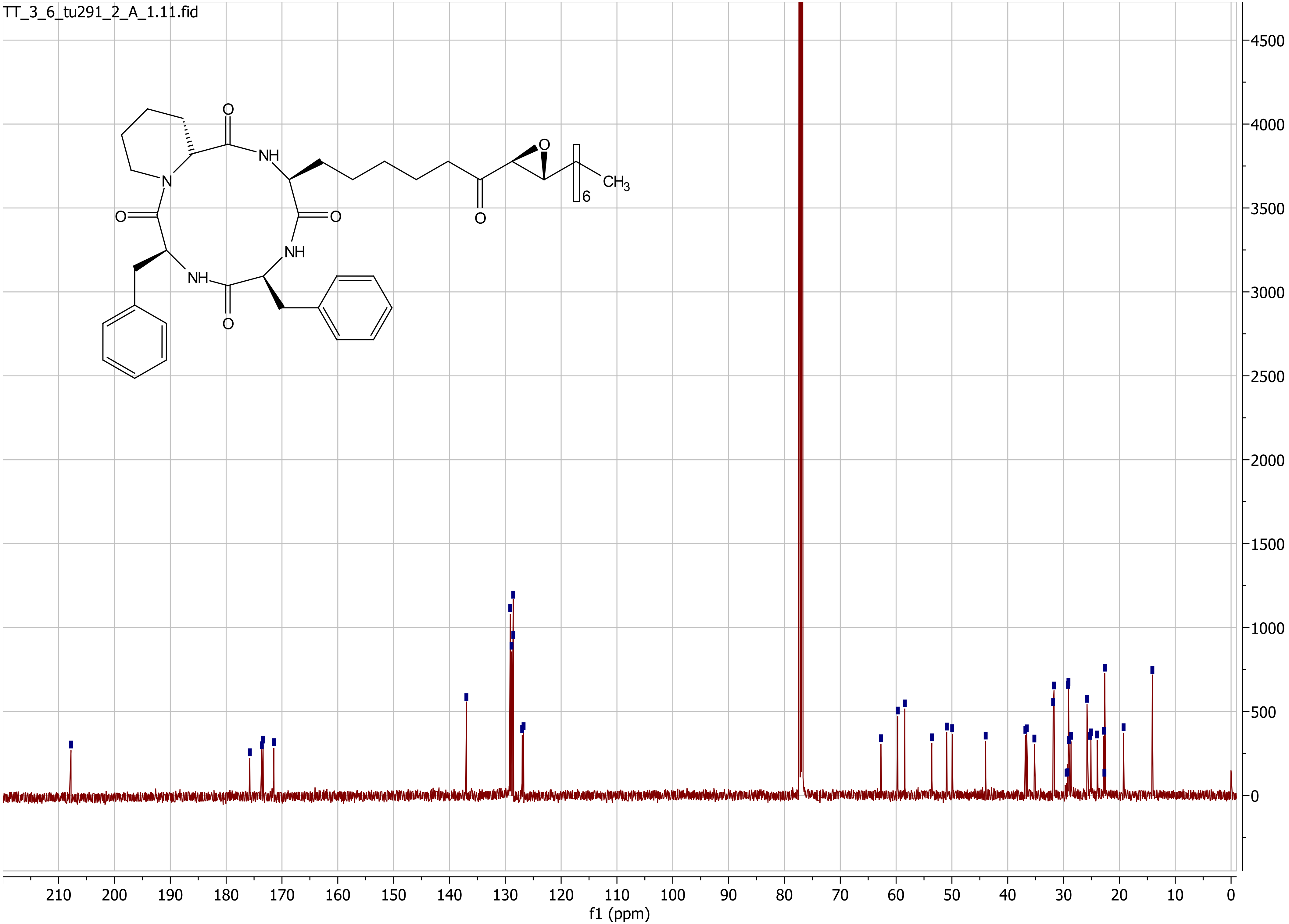

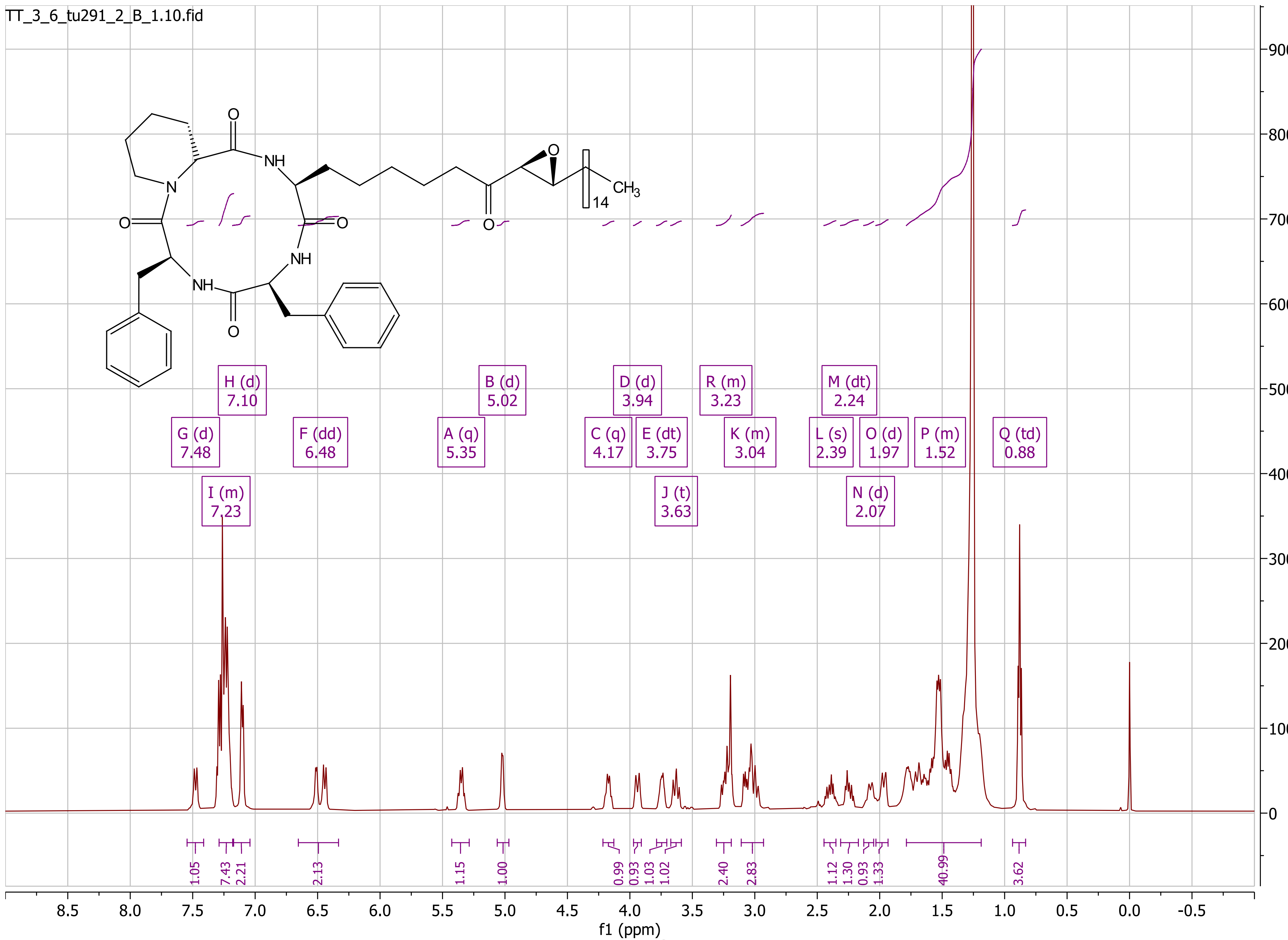
TT_3_6_tu291_2_B_1.10.fid
O
NH
N
O
O
NH
NH
O
O
O
CH3
14
G (d) 7.48
H (d) 7.10
I (m) 7.23
F (dd) 6.48
A (q) 5.35
B (d) 5.02
C (q) 4.17
D (d) 3.94
E (dt) 3.75
J (t) 3.63
R (m) 3.23
K (m) 3.04
L (s) 2.39
M (dt) 2.24
N (d) 2.07
O (d) 1.97
P (m) 1.52
Q (td) 0.88
1.05
7.43
2.21
2.13
1.15
1.00
0.99
0.93
1.03
1.02
2.40
2.83
1.12
1.30
0.93
1.33
40.99
3.62
f1 (ppm)

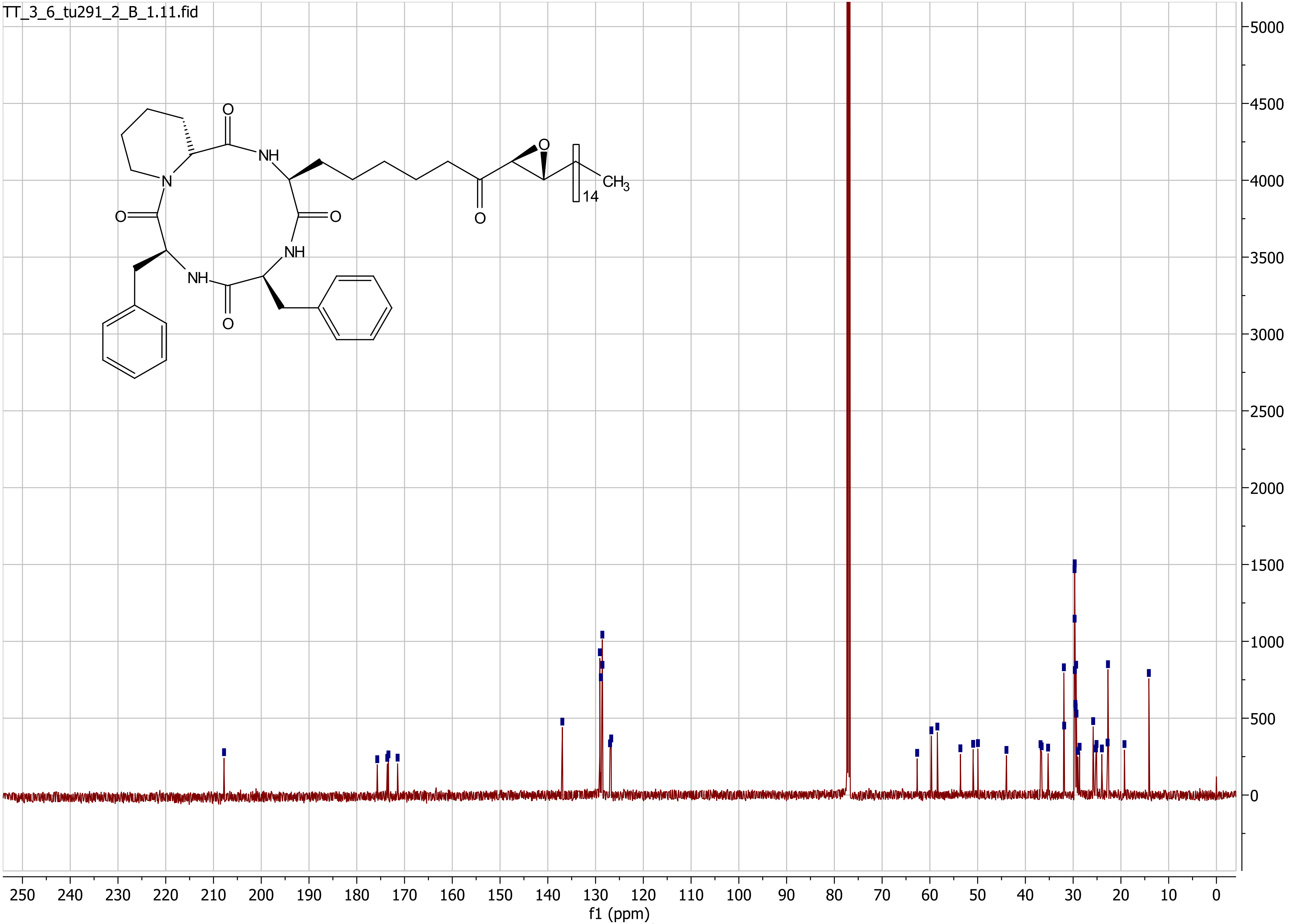
TT_3_6_tu291_2_B_1.11.fid
O
NH
N
O
O
NH
NH
O
O
O
CH3
14
5000
4500
4000
3500
3000
2500
2000
1500
1000
500
0
250 240 230 220 210 200 190 180 170 160 150 140 130 120 110 100 90 80 70 60 50 40 30 20 10 0
f1 (ppm)
